# Non-Pharmacological Interventions for Minimizing Physical Restraints Use in Intensive Care Units: An Umbrella Review

**DOI:** 10.3389/fmed.2022.806945

**Published:** 2022-04-27

**Authors:** Nianqi Cui, Xiaoli Yan, Yuping Zhang, Dandan Chen, Hui Zhang, Qiong Zheng, Jingfen Jin

**Affiliations:** ^1^Department of Nursing, The Second Affiliated Hospital Zhejiang University School of Medicine (SAHZU), Hangzhou, China; ^2^Health Management Center, The First Affiliated Hospital of Chongqing Medical University, Chongqing, China; ^3^Faculty of Nursing, Zhejiang University School of Medicine, Hangzhou, China; ^4^Key Laboratory of the Diagnosis and Treatment of Severe Trauma and Burn of Zhejiang Province, Hangzhou, China; ^5^Changxing Branch Hospital of SAHZU, Huzhou, China

**Keywords:** restraints, physical, intensive care units, critical care nursing, umbrella reviews, non-pharmacological interventions

## Abstract

**Background:**

There is a relationship between the application of physical restraints and negative physiological and psychological effects on critically ill patients. Many organizations have supported and advocated minimizing the use of physical restraints. However, it is still common practice in many countries to apply physical restraints to patients in intensive care.

**Objective:**

This study aimed to assess the effectiveness of various non-pharmacological interventions used to minimize physical restraints in intensive care units and provide a supplement to the evidence summary for physical restraints guideline adaptation.

**Methods:**

Based on the methodology of umbrella review, electronic databases, including Cochrane Database of Systematic Reviews, Joanna Briggs Institute Database of Systematic Reviews and Implementation Reports, MEDLINE, EMBASE, CINAHL, Web of Science, PsycInfo/Psyc Articles/Psychology and Behavioral Science Collection, China National Knowledge Infrastructure, SinoMed, and Wanfang Data, were searched to identify systematic reviews published from January 2016 to December 2020. Two independent reviewers undertook screening, data extraction, and quality appraisal. The methodological quality of systematic reviews was evaluated by AMSTAR 2. Evidence quality of each intervention was assessed according to GRADE. The corrected covered area was calculated as a measure of overlap.

**Results:**

A total of 47 systematic reviews were included in the umbrella review, of which six were evaluated as high quality, five were of moderate quality, and the rest were of low or critically low quality. The corrected covered area range was from 0.0 to 0.269, which indicated that there was mild overlap between systematic reviews. The included systematic reviews evaluated various types of non-pharmacological interventions for minimizing physical restraints in intensive care units, which included multicomponent interventions involving healthcare professionals' education, family engagement/support, specific consultations and communication, rehabilitation and mobilization (rehabilitation techniques, early mobilization, inspiratory muscle training), interventions related to reducing the duration of mechanical ventilation (weaning modes or protocols, ventilator bundle or cough augmentation techniques, early tracheostomy, high-flow nasal cannula), and management of specific symptoms (delirium, agitation, pain, and sleep disturbances).

**Conclusion:**

The number of systematic reviews related to physical restraints was limited. Multicomponent interventions involving healthcare professionals' education may be the most direct non-pharmacological intervention for minimizing physical restraints use in intensive care units. However, the quality of evidence was very low, and conclusions should be taken with caution. Policymakers should consider incorporating non-pharmacological interventions related to family engagement/support, specific consultations and communication, rehabilitation and mobilization, interventions related to reducing the duration of mechanical ventilation, and management of specific symptoms as part of the physical restraints minimization bundle. All the evidence contained in the umbrella review provides a supplement to the evidence summary for physical restraints guideline adaptation.

**Systematic Review Registration:**

https://www.crd.york.ac.uk/prospero/display_record.php?RecordID=242586, identifier: CRD42021242586.

## Introduction

The definition of physical restraints (PRs) is any action or procedure that prevents a person's free body movement to a position of choice and/or normal access to his/her body by the use of any method attached or adjacent to a person's body that he/she cannot control or remove easily (1). In view of numerous short- and long-term harms of PRs use in intensive care units (ICUs) (2), reduction of PRs seems to become a trend and be widely suggested. Since 2003, many organizations—including the British Association of Critical Care Nurses, American College of Critical Care Medicine, and Chinese Nursing Association—have supported and advocated minimizing the use of PRs (3–5). The development of clinical practice guidelines regarding PRs is necessary for minimizing the use of PRs (6, 7). At present, there is no guidelines regarding PRs in China. Therefore, our research team is conducting a guideline adaptation on PRs in ICUs (8). Based on the methodology of CAN-IMPLEMENT© (9), systematic review (SR) is an important supplement to the evidence summary for the adapted guideline. What's more, according to the Institute of Medicine definition of guidelines, clinical practice guidelines are statements that include recommendations intended to optimize patient care that are informed by an SR of evidence and an assessment of the benefits and harms of alternative care options (10). In other words, evidence should be informed by SRs.

Factors influencing critical care clinicians' decision on the use of PRs are multifactorial. The presence of delirium in critically ill patients was associated with the use of PRs (11). Patients who underwent mechanical ventilation (MV) were more likely to be restrained than patients who were not (12). Patients with agitation and “dangerous behaviors,” such as pulling endotracheal tube and medical devices, were also more likely to be restrained (13). Family presence may decrease PRs use or may increase their use for critically ill patients (14, 15). In our previous qualitative studies (16, 17), an inextricable link was also found between PRs and unplanned extubation, MV, pain, agitation/sedation, delirium, and family engagement. Therefore, we proposed a hypothesis that interventions that could decrease the incidence of unplanned extubation, delirium, agitation, and the duration of MV or were conducive to family-centered care would probably decrease PRs use. This also implies that SRs related to the hypothesis are helpful for the guideline adaptation of PRs. Due to PRs guideline users mainly targeted critical care nurses, this study is just focused on non-pharmacological interventions.

An umbrella review (UR) is a narrative compilation of evidence for several related clinical questions from multiple SRs and meta-analyses (MAs) into one usable document with text, tables, and graphics (18). URs often address research questions that are broader in scope than those examined in individual SRs (19). The wide picture obtainable from the conduct of an umbrella review is ideal to highlight whether the evidence base around PRs is consistent or contradictory and to explore the reasons for the findings (20). Given the decision-making of nurses dealing with the use of PRs is a complex trajectory, URs may be a more suitable approach since URs provide the best evidence on the effectiveness of the interventions evaluated in various SRs, to provide a “snapshot” to guide treating clinicians for evidence-based decisions on appropriate management approaches to achieve optimal patient outcomes.

Therefore, as part of the guideline adaptation project (8), this study aims to assess the effectiveness of various non-pharmacological interventions used to minimize PRs in ICUs and provide a supplement to the evidence summary for PRs guideline adaptation, which is helpful for critical care clinicians to make decisions on PRs. The research questions that specify PIPOH (P-Population, I-Intervention, P-Professionals, O-outcomes, H-Healthcare Setting) were as follows:

- P: adult critically ill patients;
- I: any potential non-pharmacological interventions that could decrease PRs use;
- P: mainly targeted for critical care nurses;
- O: primary outcomes: the proportion of patients physically restrained;
- H: adult ICUs.

## Method

### Protocol and Registration

The UR followed preferred reporting items for overviews of SRs, including harms checklist (21), and the protocol was registered in PROSPERO (registration No CRD42021242586) and published elsewhere (22).

### Search Strategy

The following electronic databases were searched: Cochrane Database of Systematic Reviews (CDSR), Joanna Briggs Institute Database of Systematic Reviews and Implementation Reports (JBI), Medical Literature Analysis and Retrieval System Online (MEDLINE), Excerpta Medica dataBASE (EMBASE), Cumulative Index of Nursing and Allied Health Literature (CINAHL), Web of Science (WOS), PsycInfo/Psyc Articles/Psychology and Behavioral Science Collection (Psyc), China National Knowledge Infrastructure (CNKI, for Chinese literature), SinoMed (for Chinese literature), and Wanfang Data (for Chinese literature). SRs were searched from January 2016 to January 2021. Search strategies were developed with the guidance of an expert librarian. Supplementary Table 1 provides a detailed search strategy for the EMBASE database. References lists of eligible SRs and MAs were manually searched.

### Study Selection, Eligibility Criteria, and Data Extraction

Search results were imported into a reference management software (NoteExpress, version 3.5.0). After excluding duplicates, two reviewers (NC and XY) independently completed the title and abstract screening, followed by reviewing the full-text SRs. SRs were included if they met either inclusion criterion and did not meet any of the exclusion criteria (Table 1). Data were extracted by two reviewers (NC and XY) using a data extraction form that included author, year of publication, objective, number of participants, number of studies, methodological quality, outcomes assessed, combined effect size, heterogeneity, and conclusions. Any disagreement between them was resolved by the third reviewer (YZ).

**Table 1:** Inclusion and exclusion criteria of systematic reviews and meta-analyses.

**Inclusion criteria**	**Exclusion criteria**
• Adults patients. • Non-pharmacological interventions related to the management of physical restraints, unplanned extubation, mechanical ventilation, procedural pain, agitation/sedation, delirium, and visiting/family engagement in ICUs. • Primary outcomes: the proportion of patients physically restrained; potential secondary outcomes: including but not limited to the incidence of unplanned extubation, delirium, agitation, weaning failure or successful extubation; the duration of mechanical ventilation; ICU LOS; hospital LOS; ICU mortality; mental outcomes and satisfaction. • Published in English or Chinese and after January 2016.	• Reported on children and young patients only; patients receiving end-of-life care; patients with major neurocognitive disorder/dementia; patients with intoxication and/or withdrawing from drugs or alcohol. • Reported pharmacological interventions only. • SRs without relevant outcome indicators. • Reported on patients in non-ICUs settings only. • Non-SRs. • Poor methodological quality. • No access to obtain full text.

*ICUs, intensive care units; SRs, systematic reviews; MAs, meta-analyses; LOS, length of stay*.

### Quality Assessment

Two reviewers (NC and XY) rated the methodological quality of SRs and MAs with the A MeaSurement Tool to Assess systematic Reviews (AMSTAR 2, Chinese version) (23). AMSTAR 2 consists of 16 items, and items 2, 4, 7, 9, 11, 13, and 15 are critical domains. No or one non-critical item is rated as “No,” and the methodological quality of the SR is rated as “High.” More than one non-critical items are rated as “No” without critical weaknesses, and the methodological quality of the SR is rated as “Moderate.” Only one critical item is rated as “No” with or without non-critical weaknesses, and the methodological quality of the SR is rated as “Low.” More than one critical items are rated as “No” with or without non-critical weaknesses, and the methodological quality of the SR is rated as “Critically low”, which means the SR cannot provide an accurate and comprehensive summary of the available studies (24). To increase the methodological quality of included studies, minimal requirements for the methodological quality of SRs in this study were at most one critical item was rated as “No” for SRs without MAs and at most two critical items were rated as “No” for SRs with MAs.

If authors of SRs and MAs assessed the quality of the evidence for outcomes related to this study according to Grades of Recommendation, Assessment, Development and Evaluation (GRADE) (25), it would not be repeated to assess. Otherwise, the evidence quality of each outcome was assessed GRADE. This tool allows evidence to be graded as High (high quality of evidence), Moderate (moderate quality of evidence), Low (low quality of evidence), or Very low (very low quality of evidence). Evidence can then be downgraded or upgraded on the basis of pre-specified criteria. The criteria used to downgrade evidence include study limitations, inconsistency, indirectness, imprecision, and publication bias. The criteria used to upgrade the quality of evidence are restricted to prospective cohort studies. These criteria include a large magnitude of association, a dose-response gradient, and attenuation by plausible confounding.

### Overlapping

Primary studies are often included in more than one review. Pooling the results of all of the reviews would give disproportionate statistical power to multiple primary studies and could also introduce significant overlap, which would lead to biased results (26). Corrected covered area (CCA) was calculated as a measure of overlap (26). CCA was performed only among SRs and MAs, which evaluated the same interventions and outcomes or in the same subtheme, such as family presence. The equation is described below where *N* stands for the sum of all enrolled publications (including double counting), *r* stands for the number of publications (excluding double counting), and *c* stands for the number of SRs and MAs:


(1) $$\begin{array}{l} CCA=\frac{N-r}{rc-r}\; \end{array}$$

A CCA score <5 indicates mild overlap, scores between 5–15 suggest moderate overlap, and a CCA score ≥15 suggests high overlap.

### Data Synthesis

Systematic reviews and meta-analyses that met the inclusion criteria formed the unit of analysis. Only data available from reviews were presented. Results from reviews were synthesized with narrative synthesis. Summary tables describing review characteristics and conclusions were also presented.

## Results

### Literature Search

**Figure 1:**
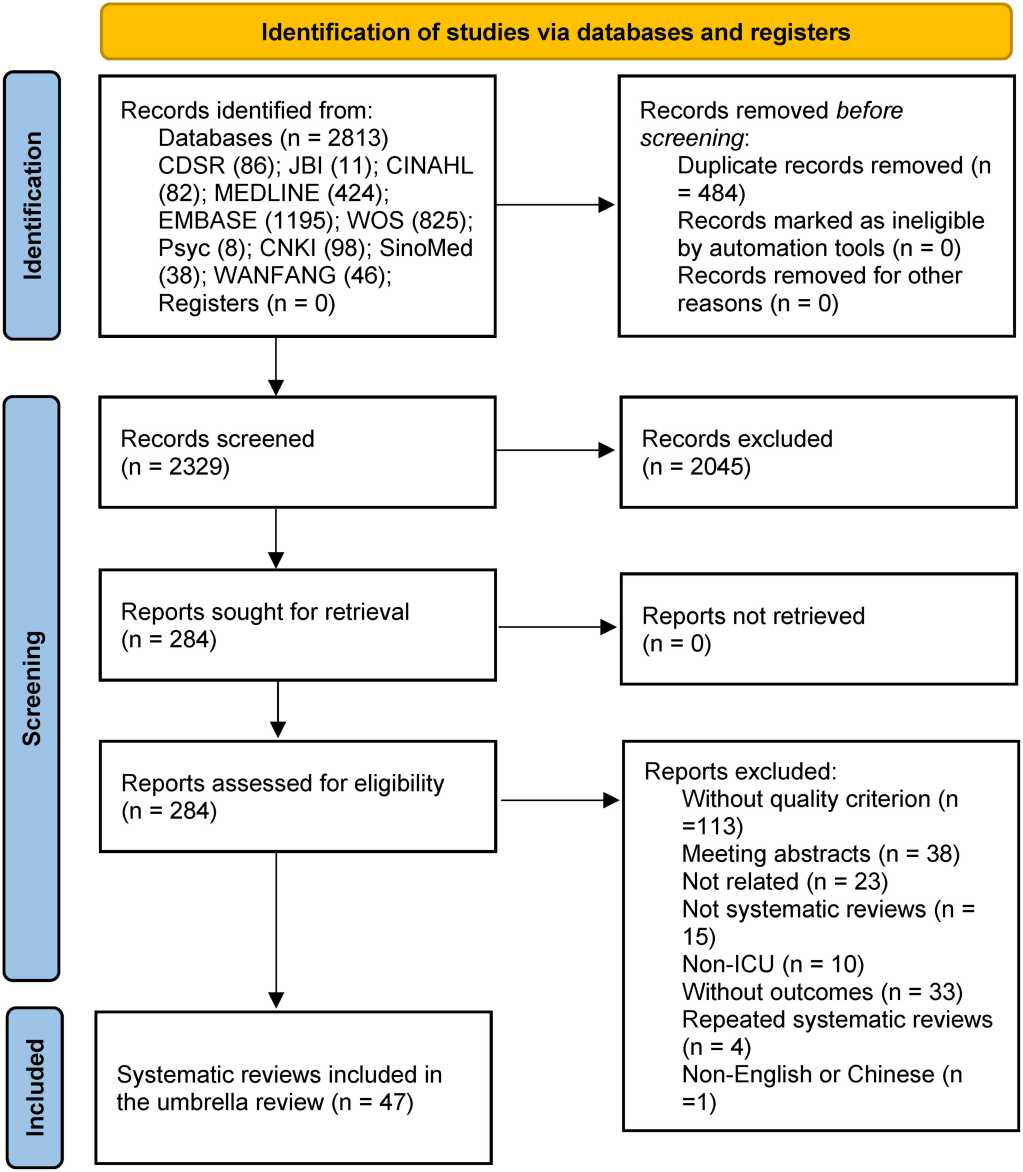
PRISMA 2020 flow diagram for new systematic reviews which included searches of databases and registers only.

A total of 2,813 records were retrieved. After excluding duplicates, 2,329 records remained. Two reviewers read each title and abstract and then identified 284 studies for full-text review. A total of 47 SRs and MAs were included in the umbrella review based on the inclusion and exclusion criteria. The flow diagram of the study selection process is shown in Figure 1. Supplementary Table 2 provides the list of excluded studies. Supplementary Table 5 provides a summary characteristics of SRs and MAs included in the UR.

### Methodological Quality

The methodological quality of 47 SRs and MAs was evaluated by AMSTAR 2, of which six were evaluated as high quality, five were evaluated as moderate quality, 19 were evaluated as low quality, and 20 were evaluated as critically low quality (Supplementary Table 3).

### Overlapping

When more than one SR was included in a subtheme, CCA was calculated. The CCA was 0.019 for family engagement/support SRs, 0.040 for specific consultations and communication SRs, 0.046 for rehabilitation techniques SRs, 0.061 for early mobilization SRs, 0.269 for inspiratory muscle training SRs, 0.044 for weaning modes or protocols SRs, 0.0 for ventilator bundle or cough augmentation techniques SRs, 0.167 for early tracheostomy SRs, 0.127 for high-flow nasal cannula SRs, 0.016 for delirium SRs, and 0.050 for agitation SRs. Supplementary Table 4 provides the citation matrix for CCA.

### Summary Findings

The included SRs evaluated various types of non-pharmacological interventions for minimizing PRs in ICUs, which included multicomponent interventions involving healthcare professionals' education, family engagement/support, specific consultations and communication, rehabilitation and mobilization (rehabilitation techniques, early mobilization, inspiratory muscle training), interventions related to reducing the duration of mechanical ventilation (weaning modes or protocols, ventilator bundle or cough augmentation techniques, early tracheostomy, high-flow nasal cannula), and management of specific symptoms (delirium, agitation, pain, and sleep disturbances). Supplementary Table 5 provides summary characteristics and quality of evidence of SRs and MAs included in the UR.

#### Multicomponent Interventions Involving Healthcare Professionals' Education

Our search identified one SR (27), which included six observational studies. Most of the interventions delivered for PRs reduction were multicomponent and included PRs use audit, use of a decision support tool, use of mitts, guideline implementation, early mobilization protocol, and healthcare professionals' education. Healthcare professionals' education was present in all multicomponent interventions. Very low quality of evidence showed that multicomponent interventions involving healthcare professionals' education could reduce the proportion of patients physically restrained (OR 0.48; 95%CI, 0.32 to 0.73).

#### Family Engagement/Support

Our literature search identified five SRs, which included 86 observational and randomized studies. Family engagement interventions included flexible visitation, witnessed resuscitation, and family involvement in basic patient care. Flexible visitation was supported by low to very low quality evidence in the reduction of delirium (OR 0.39; 95%CI: 0.22 to 0.69), anxiety symptoms of critically ill patients (MD −2.20; 95%CI: −3.80 to −0.61), compared with restrictive visitation (28). However, for ICU-acquired infection, ICU mortality, ICU LOS, depressive symptoms of patients, and anxiety of family members, flexible visitation showed no difference (very low to low) (28, 29). There was inconsistent evidence for the effect of flexible visitation on family members' satisfaction and burnout of ICU professionals (28, 30). Low-quality evidence showed that family involvement in basic patient care could reduce anxiety among relatives of critically ill patients, whereas witnessed resuscitation could not (29). In parallel, family involvement in basic patient care might decrease ICU professionals' burnout (very low) (29). Family support interventions contained medical information support, emotional support, family support coordinators, and diaries or family-maintained progress journal. The evidence suggested that medical information support could decrease ICU LOS (MD −0.89; 95%CI: −1.50 to −0.27) and hospital LOS (MD −3.78; 95%CI: −5.26 to −2.29) (high), depression (MD 0.30; 95%CI: 0.08 to 0.52) and anxiety symptoms (MD 0.40, 95%CI: 0.14 to 0.66) of ICU survivors (low), the anxiety of family members (very low) without impacting mortality of patients, and stress of family members (low) (30–32). There were differences in the impact of the timing of interventions and therapeutic goal on ICU LOS. Medical information support started within 72 h could shorten ICU LOS (MD −1.07, 95%CI: −2.12 to −0.02), while the intervention began after 72 h could not (MD −0.69, 95%CI: −1.53 to 0.14) (moderate to high) (32). Subgroup analyses showed that the therapeutic goal of comfort care setting could decrease ICU LOS (MD −1.26, 95%CI: −2.21 to −0.31), and the therapeutic goal of curative care setting could not (MD −0.61, 95%CI, −1.42 to 0.19) (moderate to high) (32). Family support coordinators might improve the satisfaction and comfort of family members (very low) (30). Low to very low quality of evidence has found that a diary written by both the family members and the ICU professionals might not decrease anxiety among relatives at ICU discharge or at 3 months (29, 30), whereas, after 12 months post-discharge, symptoms of PTSD among family members might be declined because of the use of diary (30). The use of diaries was associated with a significant reduction in depression and anxiety at 3 months among ICU survivors (very low), and no significant trend toward reduction in PTSD was found (moderate) (31).

#### Specific Consultations and Communication

We identified four SRs that included 65 observational and randomized studies. Specific consultations included interprofessional shared decision-making and ethics consultation. There was a positive correlation between interprofessional shared decision-making and patient satisfaction, and job satisfaction of ICU professionals (very low). A negative association was observed between interprofessional shared decision-making and frequency and severity of moral distress (very low) (33). Moderate to high quality of evidence proved that ethics consultation could decrease ICU LOS (MD −1.21, 95%CI: −2.25 to −0.16), while mortality could not be affected by it (34). Interventions aimed at improving surrogate decision-making, such as healthcare professional-led interventions or ethics consultation, might reduce ICU LOS among patients who die in the ICU (MD −2.11, 95%CI: −4.16 to −0.07) without impacting ICU LOS among all patients, mortality, and mental health status of family members (low to moderate) (35). Very low to low quality of evidence supported that both high-technology and low-technology augmentative and alternative communication (AAC) increased patient satisfaction and communication success without obvious difficulties in communication (36). AAC does not affect the PRs use, pain levels, heavy sedation, ICU LOS, and hospital LOS (moderate) (36).

#### Rehabilitation and Mobilization

##### Rehabilitation Techniques

Two SRs met the criteria for our assessment of the data, which included 91 observational and randomized studies. Rehabilitation techniques in critically ill patients, such as supine cycling, neuromuscular electrical stimulation, and protocolized physical therapy, did not influence mortality (low to moderate), and in the meanwhile, very low quality of evidence showed that supine cycling and neuromuscular electrical stimulation did not reduce ICU LOS, but protocolized physical rehabilitation significantly shortened ICU LOS (low) (MD −2.0, 95%CI: −3.6 to −0.3) (37). In terms of the safety of rehabilitation interventions, low quality of evidence indicated that the incidence of potential safety events, such as fall, endotracheal tube removal, intravascular catheter event, cardiac arrest, hemodynamic changes, and desaturation, was low (38).

##### Early Mobilization

Our literature search identified five SRs, which included 66 RCTs. There were differences in early mobilization definition between studies. Mobilization within 24 h or after 96 h might belong to early mobilization. Early mobilization was effective in preventing the occurrence of ICU-acquired weakness (OR 0.42, 95%CI: 0.22 to 0.82; RR 0.49, 95%CI: 0.26 to 0.91) (low) (39, 40), shortening ICU LOS (MD −1.82, 95%CI: −2.88 to −0.76) (low) (40), and improving ventilator-free days (SMD 0.17, 95%CI: 0.02 to 0.31) (high) (41). However, it had no effect on the mortality rate (e.g., 28-day mortality, ICU mortality, and hospital mortality) (moderate) (40, 41), duration of MV (low) (42), delirium-free days, and cognitive or mental health status (very low to low) (39). The evidence of the effect of early mobilization on adverse events was conflicting. High-quality evidence proved that early mobilization did not increase the incidence of adverse events (41), but another Cochrane review indicated that there was insufficient evidence on the effect of early mobilization on adverse events (low) (43). In terms of different types of physical therapy interventions, early mobilization was the most effective treatment to reduce the duration of MV compared with conventional physical therapy and inspiratory muscle training (low) (44).

##### Inspiratory Muscle Training

We identified three SRs that included 60 observational and randomized studies. Inspiratory muscle training could shorten the duration of MV (MD −2.24, 95%CI: −4.33 to −0.15; MD −4.07, 95%CI: −7.35 to −0.80) (very low to low) (45, 46) and decrease weaning failure (RR 0.66, 95%CI: 0.48 to 0.92) (moderate) (45). However, inspiratory muscle training had no effect on the reintubation and mortality (very low to moderate) (45, 46) and might or might not reduce ICU LOS (very low to moderate) (45, 46). The evidence of the effect of inspiratory muscle training on the duration of weaning was conflicting. Moderate quality of evidence proved that inspiratory muscle training could not reduce the duration of weaning compared with conventional physical therapy; however, the combination of inspiratory muscle training and routine conventional physical therapy could (very low) (44). Very low to low quality of evidence suggested that inspiratory muscle training had an effect on the duration of weaning compared with conventional physical therapy (45, 46).

#### Interventions Related to Reducing the Duration of Mechanical Ventilation

##### Weaning Modes or Protocols

Four SRs met the criteria for our assessment of the data, which included 91 observational and randomized studies. Automated weaning modes, such as proportional assist ventilation, adaptative support ventilation and Smartcare, and nurse-led weaning protocols, may have a positive impact on weaning outcomes. Low to moderate quality of evidence suggested that proportional assist ventilation was superior to pressure support ventilation in terms of weaning success (RR 1.16, 95%CI: 1.07 to 1.26) and reduced reintubation rate (RR 0.49, 95%CI: 0.28 to 0.87; RR 0.39, 95%CI: 0.17 to 0.90) (47, 48). Low quality of evidence indicated that the duration of MV was reduced in patients with proportional assist ventilation (MD −40.26, 95%CI: −66.67 to −13.48) (47, 48). In addition, ICU LOS was decreased because of the use of proportional assist ventilation (MD −1.58, 95%CI: −2.68 to −0.47), but had no effect on mortality (moderate) (47). There were conflicts in the evidence of proportional assist ventilation on the duration of weaning (47, 49). Another SR demonstrated that the use of proportional assist ventilation and neutrally adjusted ventilatory assist was associated with a reduction in the incidence with the duration of MV and weaning, ICU LOS, hospital LOS, non-invasive ventilation after extubation, and asynchrony index >10% (very low to low) (48). High quality of evidence showed that Smartcare significantly reduced the duration of weaning, and moderate quality of evidence indicated that adaptative support ventilation similarly had efficacy in the reduction of weaning (MD −0.19, 95%CI: −0.35 to −0.03) (49). Except for automated modes, weaning protocols led by nurses had a decreasing effect on the duration of MV (MD −1.69, 95%CI: −3.23 to −0.16), ICU LOS (MD −2.04, 95%CI: −2.57 to −1.52), and hospital LOS (MD −2.9, 95%CI: −4.24 to −1.56) compared with the usual physician-led care (very low to low) (50).

##### Ventilator Bundle or Cough Augmentation Techniques

We identified two SRs, which included 16 observational and randomized studies. Very low quality of evidence suggested that the ventilator bundle consisting of deep venous thrombosis prophylaxis, the elevation of the head of the bed, daily assessment of readiness to extubate, daily oral care with chlorhexidine, peptic ulcer disease prophylaxis, and daily “sedation vacations” were effective in reducing mortality (OR 0.90, 95%CI: 0.84 to 0.97), ICU LOS (SMD −0.16, 95%CI: −0.21 to −0.12), hospital LOS (SMD −0.12, 95%CI: −0.18 to −0.06), and duration of MV (SMD −0.18, 95%CI: −0.23 to −0.13) (51). Cough augmentation techniques comprised lung volume recruitment (also termed airstacking or breathstacking), manually assisted cough, mechanically assisted cough using a mechanical insufflation-exsufflation device to improve extubation success (RR 1.58, 95%CI, 1.13 to 2.20), and decreased duration of MV (MD −6.1, 95CI: −8.4 to −3.8) (very low) (52).

##### Early Tracheostomy

Our literature search identified three SRs, which included 36 observational and randomized studies. There were differences in early tracheostomy definition between studies. Tracheotomy within 2 days or 10 days might belong to early tracheostomy. Late tracheotomy generally referred to tracheotomy performed within 7–14 days among studies. Low quality of evidence proved that early tracheotomy seemed to be associated with a shorter duration of MV (SMD −0.91, 95%CI: −1.45 to −0.38), sedation (SMD −1.41, 95CI: −2.09 to −0.73), and shorter ICU stay (SMD −1.08, 95%CI: −1.61 to −0.56) without impacting ICU mortality in critically ill ventilated patients (53). For acutely brain-injured patients, early tracheostomy also might reduce long-term mortality (RR 0.57, 95%CI: 0.36 to 0.90), ICU mortality (RR 0.46, 95%CI: 0.24 to 0.89), duration of MV (MD −2.72, 95%CI: −4.15 to −1.29; MD −4.15, 95%CI: −6.30 to −1.99), ICU LOS (MD −2.55, 95%CI: −4.15 to −1.29; MD −5.87, 95%CI: −8.74 to −3.00), and hospital LOS (MD −6.68, 95%CI: −8.03 to −5.32), without impacting laryngotracheal complications, hospital mortality, and mortality (very low to low) (54, 55). However, the very low quality of evidence suggested that early tracheostomy might increase the number of tracheostomy procedures performed (54).

##### High-Flow Nasal Cannula

Six SRs met the criteria for our assessment of the data, which included 77 observational and randomized studies. Compared with conventional oxygen therapy, high-flow nasal cannula after extubation reduced post-extubation respiratory failure (RR 0.52, 95%CI: 0.30 to 0.91; RR 0.62, 95%CI: 0.42 to 0.92) (very low to high) (56, 57), MV rate (OR 0.56, 95%CI: 0.33 to 0.97) (low) (58), respiratory rates (MD −0.70, 95%CI: −1.16 to −0.25) (high) (57), support treatment failures (low to moderate) (58, 59), such as escalation of respiratory therapy to non-invasive ventilation, non-invasive positive pressure ventilation or invasive ventilation, and increased PaO_2_, but had no effect on mortality, ICU LOS, hospital LOS, respiratory infection, comfort (short-term or long-term), need for non-invasive ventilation, nasal mucosa or skin trauma, and respiratory effects (very low to high) (56, 58, 59). The evidence of the effect of the high-flow nasal cannula after extubation on reintubation was conflicting. Moderate quality of evidence suggested that high-flow nasal cannula decreased reintubation (56, 58) another high quality and low quality of evidence does not support this viewpoint (57, 60). In the peri-intubation period, low to moderate quality of evidence suggested that the use of high-flow nasal cannula likely has no effect on severe desaturation, serious complications, apneic time, oxygenation, ICU LOS, or overall mortality (61). Compared with non-invasive ventilation or non-invasive positive pressure ventilation, high-flow nasal cannula improved patient comfort (MD −1.60, 95%CI: −2.88 to −0.32) (very low to moderate) (56, 59), had no effect on respiratory infection, barotrauma, respiratory effects, the rate of escalation of respiratory, reintubation, intubation rate, post-extubation respiratory failure, treatment failure, peri-intubation complication, hospital LOS, and mortality (very-low to high) (56, 59, 60). The evidence of the effect of high-flow nasal cannula on ICU LOS was conflicting. In low quality of evidence, high-flow nasal cannula could not decrease ICU LOS (59), whereas another moderate quality of evidence proved that high-flow nasal cannula reduced ICU LOS (56).

#### Management of Specific Symptoms (Delirium, Agitation, Pain, and Sleep Disturbances)

Our literature search identified 14 SRs, which included 188 observational and randomized studies. Low quality of evidence suggested that non-pharmacological delirium-prevention interventions, such as early mobilization, family participation, patient education, music, changes to the physical environment, and multicomponent interventions (the combination of two or more of the single interventions listed), were effective in the reduction of delirium incidence (OR 0.43, 95%CI: 0.33 to 0.55), delirium duration (MD −1.43, 95%CI: −1.94 to −0.92), and ICU LOS (MD −1.24, 95%CI: −2.05 to −0.43), but the mortality, quality of life, and adverse events were not (62). Multicomponent interventions could reduce delirium incidence (RR 0.30, 95%CI: 0.10 to 0.88), delirium duration (MD −1.33, 95%CI: −1.83 to −0.83), the severity of delirium (SMD −1.39, 95%CI: −2.20 to −0.58), and ICU LOS (low to moderate) (62, 63). However, multicomponent intensive occupational therapy or physical therapy, multicomponent orientation and cognitive stimulation, and multicomponent (risk factor targeting) interventions might not decrease the incidence of delirium, delirium duration, and hospital mortality (very low to low) (62, 63). Meanwhile, single-component interventions, such as physical rehabilitation and cognitive therapy, range of motion exercises, changes to the physical environment, protocolized sedation, awakening and breathing, and structured mirrors, were not effective in the incidence of delirium, delirium duration, delirium- and coma-free days, cognitive functioning, ventilator-free days, ICU LOS, adverse events, and mortality (very low to moderate) (62, 64, 65). Early mobilization, family participation, music, and patient education appeared to have effects in reducing the incidence of delirium (low to moderate) (62). Family voice orientation and early mobilization might reduce the delirium duration (very low to moderate) (62, 64). Early goal-directed mobilization did not seem to be able to change anxiety and depression of ICU survivors (very low to low) (31). The evidence of the effect of earplugs and eye masks on delirium incidence was conflicting. Very low to low quality of evidence indicated that earplugs and eye masks might not reduce the delirium incidence (64, 66), but very low to low quality of evidence demonstrated that earplugs could decrease the incidence of delirium (67).

Bispectral index (BIS) monitors, which were based on the processing of electroencephalographic signals, had no effect in reducing ICU LOS, duration of MV, and adverse events, such as restlessness, endotracheal tube resistance, pain, and delirium, compared with clinical assessment in the titration of sedation depth (very low to low) (68). Low quality of evidence showed that protocol-directed sedation delivered by nurses could not reduce the duration of MV, ICU mortality, hospital mortality, and ICU LOS, but the hospital LOS was 3.09 days shorter (95% CI: −5.08 to −1.10) (moderate) without an increase of adverse event (self-extubation and reintubation) (low to high), compared with usual care (non-protocol-directed sedation) (69). A mild target sedation protocol with daily sedation interruption did not appear to differ in regard to the mortality and duration of MV (moderate to high) (70). However, deep sedation increased mortality (71, 72), duration of MV, and ICU LOS in mechanically ventilated patients (very low to moderate) (72), but had no effect on the incidence of delirium, hospital LOS, and incidence of agitation-related adverse events (71, 72).

Guided imagery was involved focusing one's attention on pleasant mental images to replace stressful feelings. Very low quality of evidence indicated that the effect of guided imagery on pain, anxiety, and ICU LOS was statistically significant or non-statistically significant, and there were no statistically significant results about mortality, sleep quality, and patient satisfaction (73). Music therapy was consistently associated with a reduction in anxiety and stress and an increase in sleep quality of critically ill patients (low to moderate) (74).

## Discussion

This UR synthesized existing SRs to assess the effectiveness of various non-pharmacological interventions used to minimize PRs ICUs. The SRs included multicomponent interventions involving healthcare professionals' education, family engagement/support, specific consultations and communication, rehabilitation and mobilization, interventions related to reducing the duration of mechanical ventilation, and management of specific symptoms. The number of SRs related to PRs was limited. Multicomponent interventions involving healthcare professionals' education may be the most direct non-pharmacological intervention for minimizing PRs use in ICUs. The methodological quality of included SRs was mainly low and very low quality. All the evidence contained in the URs provides a supplement to the evidence summary for PRs guideline adaptation.

There were only a limited number of SRs regarding direct non-pharmacological interventions for minimizing PRs. The results showed that multicomponent interventions involving healthcare professionals' education could reduce PRs use. These results were consistent with the present PRs guideline of Registered Nurses' Association of Ontario (RNAO), which also recommended that healthcare organizations should establish a multicomponent program including staff education (42). However, the quality of evidence in the UR was very low, and conclusions should be taken with caution.

Most non-pharmacological interventions were related to family engagement/support, specific consultations and communication, rehabilitation and mobilization, interventions related to reducing the duration of mechanical ventilation, and management of specific symptoms. According to the hypothesis of the study, these interventions may reduce the proportion of patients physically restrained. This study showed that flexible visitation decreased delirium and anxiety symptoms incidence of critically ill patients and did not increase the rate of ICU-acquired infection. Guidelines for family-centered care also suggested that family members of critically ill patients be offered open or flexible family presence at the bedside (75). Family involvement in basic patient care and ICU diaries showed a positive influence on psychological outcomes for patients and family members, which was consistent with guidelines for family-centered care (75). This UR also showed that family support interventions and specific consultations, such as medical information support and ethics consultation, had a positive impact on ICU LOS, which might help inform future updates of guidelines for family-centered care. RNAO guideline also recommended establishing communication responsibilities and debriefing procedures for client/family/substitute decision-makers and the interprofessional team (42). In accordance with the RNAO guideline, this UR also showed that there was a positive correlation between interprofessional shared-making and nurse satisfaction, and both high-technology and low-technology AAC increased clinician-patient communication and patient satisfaction. The UR found that rehabilitation techniques, such as protocolized physical rehabilitation, significantly shortened ICU LOS. This finding is consistent with that of the prevention and management of pain, agitation/sedation, delirium, immobility, and sleep disruption guideline (PADIS), which suggests performing rehabilitation or mobilization in critically ill adults (76). In the UR, early mobilization was effective in preventing the occurrence of ICU-acquired weakness, shortening ICU LOS, and improving ventilator-free days. These results further supported the recommendations of the guideline published by the American Thoracic Society/American College of Chest Physicians (ATS/ACCP), which suggested early mobilization for acutely hospitalized adults who have been mechanically ventilated for more than 24 h (77). This study found that inspiratory muscle training could shorten the duration of MV and decrease weaning failure. There is no recommendation on inspiratory muscle training in PADIS guideline at present, and this result may provide recommendations for future updates of PADIS guidelines. Given the fact that patients who underwent MV were more likely to be restrained than patients who were not (12), shortening the duration of MV may help to reduce the use of PRs. This study found that proportional assist ventilation had efficacy in the reduction of weaning, reintubation rate, and ICU LOS compared with pressure support ventilation. Smartcare and adaptative support ventilation also had the potential to reduce the duration of weaning. This study also found that the ventilator bundle was effective in reducing the duration of MV. These findings reflected the recommendation of the ATS/ACCP guideline, which also suggested ventilator liberation protocol for mechanically ventilated patients (77). The UR also indicated that early tracheostomy could reduce the duration of MV, but might increase the number of tracheostomy procedures performed. However, the recommendation of early tracheostomy should fully consider the patient's values and preferences. In addition to the interventions to shorten the duration of MV by increasing the successful weaning rate, reducing the post-extubation respiratory failure rate is also one of the interventions to shorten the duration of MV. This UR found that high-flow nasal cannula could reduce post-extubation respiratory failure and treatment failure. The evidence of the management of MV might help inform future updates of guidelines of liberation from MV. The management of specific symptoms may be one of the important interventions to minimize PRs. To reduce and prevent delirium incidence, duration, and severity, multicomponent interventions (such as family participation, patient education, music, changes to the physical environment, and early mobilization) were appropriate, which corroborated the recommendations of PADIS guideline (76). In this UR, music therapy could reduce the anxiety and stress of critically ill patients and increase their sleep quality. The PADIS guideline also suggested offering music therapy to relieve both non-procedural and procedural pain (76), which could not be supported by this UR. PADIS guideline also suggested using noise and light reduction strategies to improve sleep (76). In this study, the strategies related to light and noise reduction were eye masks and earplugs, but there was no evidence that eye masks and earplugs could improve sleep, and there was evidence that both of them had no effect on the change of mental health state. The results of this study indicated that deep sedation increased mortality, MV duration, and ICU LOS. This finding was consistent with PADIS guideline and the guideline published by ACCP/ATS, which both suggested light sedation or minimize sedation (76, 78). The UR showed that guided imagery might have an impact on pain, anxiety, and ICU LOS and may provide recommendations for future updates of PADIS guidelines.

The quality of a UR is determined by the methodological quality and the level of evidence of the available SRs. The methodological quality of included SRs was mainly low and very low quality. Among the 47 SRs evaluated by AMSTAR 2, only six SRs were evaluated as high quality, five were rated as moderate quality, and the rest were all rated as low quality or critically low quality. In the quality assessment process, 113 SRs were excluded as not fulfilling the quality criteria. According to AMSTAR 2, SRs in which methodological quality was rated as critically low should not be relied on to provide an accurate and comprehensive summary of the available studies (24). For this reason, we encourage authors to register study protocols that include methodological aspects, in order to improve the methodological quality of SRs. Overlap of primary studies between SRs can bias the results of a UR (79). The degree of overlap across all SRs was graded as being small (CCA <5); therefore, the overall degree of overlap between all SRs within this UR was slight. The overall quality of the evidence in this UR was mainly low and very low quality, which means that we are uncertain about the study results. However, this result also defines the quality of evidence of some interventions, which could help to make decisions for clinical practitioners. Meanwhile, in our previous study (80), we have identified eight guidelines related to PRs. As evidence in rapidly evolving fields may become quickly outdated, the eight guidelines were developed between 2012 and 2019, which means existing guidelines are not sufficiently current. SRs in the UR could provide a supplement to the evidence summary for our guideline adaptation.

Several limitations to this study need to be acknowledged. Due to resource constraints, we only searched for English and Chinese SRs and MAs. Additionally, since we only included SRs and MAs in the past 5 years, there may be a loss of SRs and MAs published before 2016. With the AMSTAR 2 quality appraisal instrument, 113 SRs and MAs were rated as critically low, and those that did not meet the methodological quality criteria were excluded, which might lead to bias. Although the SRs and MAs we included did not involve pharmaceuticals, some of these interventions potentially involve pharmacological interventions. The majority of the included SRs and MAs were lack of the incidence of PRs, which might lead to down-grading because of indirectness. Most SRs and MAs were rated as critically low, which could have inadvertently led to a downgrading of the quality of the UR.

## Conclusion

The number of systematic reviews related to PRs was limited. Multicomponent interventions involving healthcare professionals' education may be the most direct non-pharmacological intervention for minimizing PRs use in intensive care units. However, the quality of evidence was very low, and conclusions should be taken with caution. Policymakers should consider incorporating non-pharmacological interventions related to family engagement/support, specific consultations and communication, rehabilitation and mobilization, interventions related to reducing the duration of mechanical ventilation, and management of specific symptoms as part of the PRs minimization bundle. All the evidence contained in the UR provides a supplement to the evidence summary for PRs guideline adaptation.

## Data Availability Statement

The original contributions presented in the study are included in the article/Supplementary Material, further inquiries can be directed to the corresponding author.

## Author Contributions

NC and JJ conceived and planned the umbrella review. NC carried out the literature search and took the lead in writing the manuscript with critical input from DC, HZ, and QZ. NC, XY, and YZ contributed to the assessment of methodological quality and screened the records and extracted the data. All authors read and approved the final manuscript.

## Funding

This work was supported by the Science Research Foundation of Chinese Nursing Association [Grant Number: ZHKY201913], Zhejiang University Academic Award for Outstanding Doctoral Candidates [Grant Number: 202059], and Chongqing Science and Health Collaborated Medical Research Project [Grant Number: 2021MSXM157]. The funders had no role in the design and conduct of the study; collection, management, analysis, and interpretation of the data; preparation, review, or approval of the manuscript; and decision to submit the manuscript for publication.

## Conflict of Interest

The authors declare that the research was conducted in the absence of any commercial or financial relationships that could be construed as a potential conflict of interest.

## Publisher's Note

All claims expressed in this article are solely those of the authors and do not necessarily represent those of their affiliated organizations, or those of the publisher, the editors and the reviewers. Any product that may be evaluated in this article, or claim that may be made by its manufacturer, is not guaranteed or endorsed by the publisher.
