# Supplementary material for: Non-Pharmacological Interventions for Minimizing Physical Restraints Use in Intensive Care Units: An Umbrella Review
Source: Front Med (Lausanne). 2022 Apr 27;9:806945. doi: 10.3389/fmed.2022.806945 (PMC9091438; doi:10.3389/fmed.2022.806945)
Supplement: Supplementary file 1 [file Data_Sheet_1.docx]

Supplementary Material

# Supplementary Figures and Tables

**Table 1 Search strategy**

| Search strategy for EMBASE |
| --- |
| #1 'physical restraint'/exp  #2 ((physical OR mechanical) NEAR/2 (constraint* OR restraint*)): ab,ti  #3 ((ankle* OR appendage* OR arm* OR body OR bodies OR foot OR feet OR hand* OR leg* OR limb* OR patient* OR wrist*) NEAR/2 (tie* OR bedrail* OR belt* OR strap* OR mitten* OR jacket*)): ab,ti  #4 'extubation'/exp  #5 'mechanical ventilator'/exp  #6 'artificial ventilation'/exp  #7 ((unplanned OR self OR accidental OR ventilat*) NEAR/2 (extubat* OR reintubat*)): ab,ti  #8 (((mechanic* OR artificial OR 'positive pressure') NEAR/2 ('ventilat* OR respirat*' OR breathing)):ab,ti) OR ((ventilat* NEAR/2 (wean* OR liberat*)):ab,ti)  #9 'aggression'/exp  #10 'restlessness'/exp  #11 'sedation'/exp  #12 'behavior control'/exp  #13 ((abusive OR challenging OR disturbed OR disruptive OR aggression OR agonistic OR restless* OR anger OR angry OR assault OR rage* OR hostil* OR threat* OR violen*) NEAR/2 (behav*): ab,ti  #14 'daily sedation interruption': ab,ti OR 'crisis intervention': ab,ti  #15 'delirium'/exp  #16 'disorders of higher cerebral function'/exp  #17 delirious*: ab,ti  #18 ((abnormal* OR change* OR dysfunction* OR decline OR deterioration OR diminish* OR disorders OR mental* OR disturbed*) NEAR/2 (attention OR cognitive OR consciousness OR perception OR neurocognitive)): ab,ti  #19 (acute NEAR/2 ('brain syndrome' OR 'cerebral insufficiency')): ab,ti  #20 'procedural pain'/exp  #21 'postoperative pain'/exp  #22 'postoperative analgesia'/exp  #23 'pain measurement'/exp  #24 ((procedural OR postoperative OR management OR measurement) NEAR/2 (discomfort OR ache*)): ab,ti  #25 'shared decision making'/exp  #26 'family separation'/exp  #27 'visiting nursing service'/exp  #28 ((open OR flexible OR restrictive OR family) NEAR/2 (visitation OR visiting)): ab,ti  #29 (('patient cent?red' OR 'family cent?red') NEAR/2 (care OR nursing)): ab,ti  #30 ((shared OR sharing) NEAR/2 ('decision-making' OR 'decision making')): ab,ti  #31 'intensive care unit'/exp  #32 'intensive care'/exp  #33 'intensive care nursing'/exp  #34 ((intensive OR critical OR acute) NEAR/2 care): ab,ti  #35 ((cardiac OR coronary OR heart OR burn OR respiratory OR surgical OR surger* OR 'high dependency' OR stepdown OR 'step down' OR 'speciali#ed weaning' OR postoperati* OR 'post operati*' OR postsurg* OR 'post surg*') NEAR/2 (unit* OR centre* OR center*)): ab,ti  #36 icu*:ab,ti OR sicu*:ab,ti OR ccu*:ab,ti OR eicu*:ab,ti OR hdu*:ab,ti OR sdu*:ab,ti OR edsdu*: ab,ti  #37 #1 OR #2 OR #3 OR #4 OR #5 OR #6 OR #7 OR #8 OR #9 OR #10 OR #11 OR #12 OR #13 OR #14 OR #15 OR #16 OR #17 OR #18 OR #19 OR #20 OR #21 OR #22 OR #23 OR #24 OR #25 OR #26 OR #27 OR #28 OR #29 OR #30  #38 #31 OR #32 OR #33 OR #34 OR #35 OR #36  #39 #37 AND #38  #40 #37 AND #38 AND ([cochrane review]/lim OR [systematic review]/lim OR [meta analysis]/lim) AND ([chinese]/lim OR [english]/lim) AND ([adult]/lim OR [young adult]/lim OR [middle aged]/lim OR [aged]/lim OR [very elderly]/lim) AND [humans]/lim AND [2016-2021]/py |

**Table 2 Excluded studies with reasons**

| **Without quality criterion (n=113)** |
| --- |
| 1. 童孜蓉, 许彬, 杨炜娟, 赵琳. 脑电双频指数监测在ICU机械通气患者中的应用效果的Meta分析. 中华神经医学杂志. 2018;17(11):1137-1142  2. Bilgili B, Montoya JC, Layon AJ et al. Utilizing Bi-Spectral Index (BIS) for the monitoring of sedated adult ICU patients: a systematic review. Minerva Anestesiol. 2017;83(3):288-301.  3. Anestis DM, Tsitsopoulos PP, Tsonidis CA, Foroglou N. The current significance of the FOUR score: A systematic review and critical analysis of the literature. J Neurol Sci. 2020;409:116600  4. Birkedal HC, Larsen MH, Steindal SA, Solberg MT. Comparison of two behavioural pain scales for the assessment of procedural pain: A systematic review. Nurs Open. 2021;8(5):2050-2060.  5. Pinheiro A, Marques R. Behavioral Pain Scale and Critical Care Pain Observation Tool for pain evaluation in orotracheally tubed critical patients. A systematic review of the literature. Rev Bras Ter Intensiva. 2019;31(4):571-581  6. De Sá Araújo Freitas AFF, Fernandes M, Marques RMD, Freitas JSR. Applicability of scales/indicators for pain monitoring in critically ill patients incapable of verbalizing: A systematic review of the literature. Revista de la Sociedad Espanola del Dolor. 2019;26(5):293-303  7. Gelinas C, Berube M, Chevrier A et al. Delirium Assessment Tools for Use in Critically Ill Adults: A Psychometric Analysis and Systematic Review. Crit Care Nurse. 2018;38(1):38-49  8. Krewulak KD, Stelfox HT, Ely EW, Fiest KM. Risk factors and outcomes among delirium subtypes in adult ICUs: A systematic review. J Crit Care. 2020;56:257-264  9. 李真, 李奇, 李尊柱 et al. ICU患者亚谵妄综合征患病率及危险因素的系统评价. 中华护理杂志. 2020;55(2):288-293  10. Wang CT, Mao Y, Zhao L, Ma B. The impact of analgosedation on mortality and delirium in critically ill patients: A systematic review and meta-analysis. Intensive Crit Care Nurs. 2019;54:7-14  11. Rood P, Huisman-de WG, Vermeulen H et al. Effect of organisational factors on the variation in incidence of delirium in intensive care unit patients: A systematic review and meta-regression analysis. Aust Crit Care. 2018;31(3):180-187  12. 潘燕彬, 江智霞, 张晶晶 et al. ICU成人患者谵妄危险因素的Meta分析. 中国护理管理. 2018;18(04):465-475  13. 邢焕民, 于思淼, 吕冬梅, 范宇莹. 重症监护病房患者术后发生谵妄危险因素的Meta分析. 现代临床护理. 2018;17(01):1-9  14. 郑春艳, 张欢欢, 杨玉金, 王苹. 重症监护病房患者发生谵妄危险因素的Meta分析. 实用临床医学(江西). 2017;18(7):74-83  15. 王晓晔, 都汶妮, 陶秀彬. 缺氧与ICU谵妄关系的Meta分析. 皖南医学院学报. 2016;35(04):391-394  16. Rosgen BK, Krewulak KD, Stelfox HT et al. The association of delirium severity with patient and health system outcomes in hospitalised patients: a systematic review. Age Ageing. 2020;49(4):549-557  17. Krewulak KD, Stelfox HT, Leigh JP, Ely EW, Fiest KM. Incidence and Prevalence of Delirium Subtypes in an Adult ICU: A Systematic Review and Meta-Analysis. Crit Care Med. 2018;46(12):2029-2035  18. Serafim RB, Soares M, Bozza FA et al. Outcomes of subsyndromal delirium in ICU: a systematic review and meta-analysis. Crit Care. 2017;21(1):179  19. Honarmand K, Rafay H, Le J et al. A Systematic Review of Risk Factors for Sleep Disruption in Critically Ill Adults. Crit Care Med. 2020;48(7):1066-1074  20. Locihova H, Axmann K, Ziakova K. Sleep-disrupting effects of nocturnal nursing interventions in intensive care unit patients: A systematic review. J Sleep Res. 2020;(e13223)  21. Horsten S, Reinke L, Absalom AR, Tulleken JE. Systematic review of the effects of intensive-care-unit noise on sleep of healthy subjects and the critically ill. Brit J Anaesth. 2018;120(3):443-452  22. Schwab KE, Ronish B, Needham DM et al. Actigraphy to Evaluate Sleep in the Intensive Care Unit A Systematic Review. Ann Am Thorac Soc. 2018;15(9):1075-1082  23. 蒋玲洁, 岳伟岗, 王盛均 et al. 非药物干预比较改善ICU患者睡眠质量效果的网状Meta分析. 中国循证医学杂志. 2020;20(4):403-411  24. Cooke M, Ritmala-Castren M, Dwan T, Mitchell M. Effectiveness of complementary and alternative medicine interventions for sleep quality in adult intensive care patients: A systematic review. Int J Nurs Stud. 2020;107(103582)  25. Weiss B, Spies C, Piazena H et al. Exposure to light and darkness and its influence on physiological measures of intensive care unit patients-a systematic literature review. Physiol Meas. 2016;37(9):R73-R87  26. Ju XX, Yang J, Liu XX. A systematic review on voiceless patients' willingness to adopt high-technology augmentative and alternative communication in intensive care units. Intensive Crit Care Nurs. 2020:102948  27. Zaga CJ, Berney S, Vogel AP. The Feasibility, Utility, and Safety of Communication Interventions With Mechanically Ventilated Intensive Care Unit Patients: A Systematic Review. Am J Speech Lang Pathol. 2019;28(3):1335-1355  28. Ten HS, Elbers PW, Girbes AR, Tuinman PR. Communicating with conscious and mechanically ventilated critically ill patients: a systematic review. Crit Care. 2016;20(1):333  29. Li Y, Li H, Zhang D. Comparison of T-piece and pressure support ventilation as spontaneous breathing trials in critically ill patients: a systematic review and meta-analysis. Crit Care. 2020;24(1):67  30. Lombardi FS, Cotoia A, Petta R et al. Prediction of extubation failure in Intensive Care Unit: systematic review of parameters investigated. Minerva Anestesiol. 2019;85(3):298-307  31. Kwong MT, Colopy GW, Weber AM, Ercole A, Bergmann JHM. The efficacy and effectiveness of machine learning for weaning in mechanically ventilated patients at the intensive care unit: a systematic review. Bio-Design and Manufacturing. 2019;2(1): 31-40.  32. Soleymani S, Sheikh S, Ahmadi EA, Zade MAH. Determine the ideal time for weaning of patients from mechanical ventilation: A literature review. Indian Journal of Public Health Research and Development. 2017;8(2):279-284  33. Pandian V, Boisen S, Mathews S, Brenner MJ. Speech and Safety in Tracheostomy Patients Receiving Mechanical Ventilation: A Systematic Review. Am J Crit Care. 2019;28(6):441-450  34. Adly A, Youssef TA, El-Begermy MM, Younis HM. Timing of tracheostomy in patients with prolonged endotracheal intubation: a systematic review. Eur Arch Otorhinolaryngol. 2018;275(3):679-690  35. Singh RK, Saran S, Baronia AK. The practice of tracheostomy decannulation-a systematic review. J Intensive Care. 2017;5:38  36. Wang J, Ren D, Liu Y et al. Effects of early mobilization on the prognosis of critically ill patients: A systematic review and meta-analysis. Int J Nurs Stud. 2020;110:103708  37. 唐光明, 杨娜, 雷云宏 et al. 早期康复对重症病人生活质量影响的Meta分析. 循证护理. 2020;6(09):869-877  38. 冯梦龙, 廖雪丽, 朱妮, 高利红, 周满红. T管与压力支持通气影响脱机患者结局的Meta分析. 海南医学. 2020;31(10):1327-1331  39. 庞雪莲, 蔡甜甜, 朱超奇. 持续与间歇声门下吸引预防呼吸机相关性肺炎效果的Meta分析. 中华急危重症护理杂志. 2020;1(02):167-174  40. 姚丽, 杨琳, 杨丽平 et al. 机械通气患者吸气肌训练效果的Meta分析. 中华护理杂志. 2020;55(01):141-147  41. Vijayaraghavan BKT, Hamed S, Jain A et al. Evidence Supporting Clinical Use of Proportional Assist Ventilation: A Systematic Review and Meta-Analysis of Clinical Trials. J Intensive Care Med. 2020;35(7):627-635  42. Mendis N, Hamilton GM, McIsaac DI et al. A Systematic review of the impact of surgical special care units on patient outcomes and health care resource utilization. Anesth Analg. 2019;128(3):533-542  43. Vagionas D, Vasileiadis I, Rovina N et al. Daily sedation interruption and mechanical ventilation weaning: A literature review. Anaesthesiology Intensive Therapy. 2019;51(5):380-389  44. 孙烯辉, 杨丽, 黄德斌 et al. 机械通气患者胸部物理治疗效果的Meta分析. 护理学报. 2019;26(17):31-36  45. 杨丽平, 张志刚, 张彩云 et al. 早期活动对机械通气患者病死率影响的累积Meta分析. 中华护理杂志. 2019;54(06):843-849  46. 简佳庆, 段凤英, 肖远超, 张思颖, 高飞. ICU拔除气管插管患者使用经鼻高流量湿化氧疗对比传统氧疗或无创正压通气临床疗效的Meta分析. 中国呼吸与危重监护杂志. 2019;18(1):42-50  47. 梁冰, 张莉, 张可, 席建宏, 颜萍. 早期活动在ICU机械通气患者获得性衰弱干预应用效果的Meta分析. 中华现代护理杂志. 2019;25(24):3091-3096  48. Yang Q, Du JL, Shao F. Mortality rate and other clinical features observed in Open vs closed format intensive care units A systematic review and meta-analysis. Medicine. 2019;98(e1626127)  49. Guilhermino MC, Inder KJ, Sundin D. Education on invasive mechanical ventilation involving intensive care nurses: a systematic review. Nurs Crit Care. 2018;23(5):245-255  50. 李丽莉, 代冰, 董新新. 患者拔除气管插管后应用经鼻高流量氧疗效果的Meta分析. 中华护理杂志. 2018;53(12):1492-1497  51. 李鑫, 王玉梅, 熊莉娟, 黄海燕. 重症监护病房机械通气患者早期活动有效性与安全性的Meta分析. 现代临床护理. 2018;17(10):34-41  52. 董英, 赵营宇, 周光霞, 刘培培, 辛霞. 国内集束化护理对ICU呼吸机相关性肺炎发生率影响的Meta分析. 全科护理. 2018;16(13):1541-1544  53. 詹梦梅, 王建宁, 周松, 黄秋霞, 李立群. 间歇声门下吸引预防ICU患者呼吸机相关性肺炎的Meta分析. 中国护理管理. 2018;18(02):206-213  54. 刘华, 谭继平, 雷微, 严若芬, 谭德彩. 我国ICU机械通气患者集束化护理干预效果的Meta分析. 中国实用护理杂志. 2018;34(20):1596-1600,封3  55. 杨丽平, 张志刚, 张彩云 et al. 机械通气患者早期主动活动效果的Meta分析. 中国护理管理. 2017;17(06):758-764  56. 蒋世双, 苟小红, 李丹, 王念, 唐泽. 早期气管切开对重型颅脑损伤患者预后影响的Meta分析. 重庆医学. 2017;46(05):654-657  57. 杨丽平, 张彩云, 张志刚 et al. 机械通气患者床头抬高不同角度效果的Meta分析. 国际护理学杂志. 2017;36(14):1873-1878,1905  58. 孙小文, 张加乐, 江婷 et al. 应用声门下分泌物引流对ICU机械通气患者综合效果的Meta分析. 中华危重病急救医学. 2017;29(7):586-591  59. Tipping CJ, Harrold M, Holland A et al. The effects of active mobilisation and rehabilitation in ICU on mortality and function: a systematic review. Intens Care Med. 2017;43(2):171-183  60. Pellegrini JA, Moraes RB, Maccari JG et al. Spontaneous Breathing Trials With T-Piece or Pressure Support Ventilation. Respir Care. 2016;61(12):1693-1703  61. Chung MS, Huang CC, Yeh SL et al. The Effectiveness of Pulmonary Rehabilitation for Critical-Care Ventilator Patients: A Systematic Review. Hu Li Za Zhi. 2016;63(3):94-104  62. 吴佼佼, 马红梅, 廖春霞, 张倩, 徐旭. 集束化护理预防ICU患者呼吸机相关性肺炎的meta分析. 职业与健康. 2016;32(24):3336-3340  63. Meng L, Wang C, Li J, Zhang J. Early vs late tracheostomy in critically ill patients: a systematic review and meta-analysis. Clin Respir J. 2016;10(6):684-692  64. 王雅婧, 王宝华, 王红阳, 李志强, 浦践一. 肥胖对ICU机械通气患者预后影响的系统评价. 慢性病学杂志. 2017;(6):606-610  65. Zayed Y, Kheiri B, Barbarawi M et al. Effects of neuromuscular electrical stimulation in critically ill patients: A systematic review and meta-analysis of randomised controlled trials. Aust Crit Care. 2020;33(2):203-210.  66. 刘苗, 罗健, 朱晓敏, 谢霖, 刘杨. 神经肌肉电刺激预防ICU获得性衰弱效果的Meta分析. 护理研究. 2020;34(17):3060-3067  67. 张桂宁, 杨丽, 郭明娟 et al. 神经肌肉电刺激对ICU机械通气病人影响的Meta分析. 护理研究. 2019;33(02):187-194  68. Yang R, Zheng Q, Zuo D, Zhang C, Gan X. Safety Assessment Criteria for Early Active Mobilization in Mechanically Ventilated ICU Subjects. Resp Care. 2021;66(2):307-315.  69. 丁楠楠, 姚丽, 张志刚 et al. ICU机械通气患者早期目标导向康复锻炼安全标准的系统评价. 中华危重病急救医学. 2019;31(1):91-97  70. Roberson AR, Starkweather A, Grossman C, Acevedo E, Salyer J. Influence of muscle strength on early mobility in critically ill adult patients: Systematic literature review. Heart Lung. 2018;47(1):1-9  71. Schwab KE, To AQ, Chang J et al. Actigraphy to Measure Physical Activity in the Intensive Care Unit: A Systematic Review. J Intensive Care Med. 2020;35(11):1323-1331.  72. Nuwi D, Irwan AMI. Effect of Active Mobilization on Patients in the Intensive Care Unit: A Systematic Review. Int J Caring Sci. 2018;11(3):1942-1953  73. Krupp A, Steege L, King B. A systematic review evaluating the role of nurses and processes for delivering early mobility interventions in the intensive care unit. Intens Crit Care Nur. 2018;47:30-38  74. de Queiroz RS, Saquetto MB, Martinez BP et al. Evaluation of the description of active mobilisation protocols for mechanically ventilated patients in the intensive care unit: A systematic review of randomized controlled trials. Heart Lung. 2018;47(3):253-260  75. Bristol AA, Schneider CE, Lin S, Brody AA. A Systematic Review of Clinical Outcomes Associated With Intrahospital Transitions. J Healthc Qual. 2020;42(4):175-187  76. 李静逸, 洪洋, 宋春梅, 吴娟. ICU护士对患者身体约束体验的Meta整合. 中华护理杂志. 2018;53(10):1249-1255  77. Kerckhoffs MC, Kant M, van Delden JJM et al. Selecting and evaluating decision-making strategies in the intensive care unit: A systematic review. J Crit Care. 2019;51:39-45  78. Au SS, Couillard P, Roze DOA et al. Outcomes of Ethics Consultations in Adult ICUs: A Systematic Review and Meta-Analysis. Crit Care Med. 2018;46(5):799-808  79. 王烁. ICU患者创伤后应激障碍危险因素与非药物干预的研究. 福建医科大学. 2017  80. Nikayin S, Rabiee A, Hashem MD et al. Anxiety symptoms in survivors of critical illness: a systematic review and meta-analysis. Gen Hosp Psychiat. 2016;43:23-29  81. 敖博, 戴安, 和世琼. 以家庭为中心的护理对ICU病人家属干预效果的系统评价. 循证护理. 2019;5(6):481-486  82. 陈俊杉, 范杰梅, 余金甜, 张爱琴. ICU患者机械通气期间真实体验研究的Meta整合. 中国实用护理杂志. 2020;36(16):1274-1281  83. Kaier K, Heister T, Motschall E et al. Impact of mechanical ventilation on the daily costs of ICU care: a systematic review and meta regression. Epidemiol Infect. 2019;147:e314  84. Wilcox ME, Vaughan K, Chong CAKY, Neumann PJ, Bell CM. Cost-Effectiveness Studies in the ICU: A Systematic Review. Crit Care Med. 2019;47(8):1011-1017  85. Costa DK, White MR, Ginier E et al. Identifying Barriers to Delivering the Awakening and Breathing Coordination, Delirium, and Early Exercise/Mobility Bundle to Minimize Adverse Outcomes for Mechanically Ventilated Patients: A Systematic Review. Chest. 2017;152(2):304-311  86. Ariffin SM, Ludin SM, Arifin SRM. Being voiceless: A review on patient communication in intensive care unit. Systematic Reviews in Pharmacy. 2020;11(12):1328-1333  87. Laurent H, Aubreton S, Richard R et al. Systematic review of early exercise in intensive care: A qualitative approach. Anaesth Crit Care Pa. 2016;35(2):133-149  88. Hunter A, Johnson L, Coustasse A. Reduction of Intensive Care Unit Length of Stay: The Case of Early Mobilization. The health care manager. 2020;39(3):109-116  89. 金玲丽, 袁莉萍, 江海娇 et al. 镇静镇痛集束护理对ICU病人谵妄影响的Meta分析. 循证护理. 2020;6(8):762-769  90. 吴雨晨, 姜变通, 丁楠楠 et al. 不同探视制度对ICU患者ICU获得性感染及相关因素影响的Meta分析. 中国感染控制杂志. 2020;19(1):20-29  91. Deemer K, Zjadewicz K, Fiest K et al. Effect of early cognitive interventions on delirium in critically ill patients: a systematic review. Can J Anesth. 2020;67(8):1016-1034  92. Janssen TL, Alberts AR, Hooft L et al. Prevention of postoperative delirium in elderly patients planned for elective surgery: systematic review and meta-analysis. Clin Interv Aging. 2019;14:1095-1117  93. Zhang J, Zhao X, Wang A. Early rehabilitation to prevent post-intensive care syndrome in critical illness patients: a Meta-analysis. Zhonghua Wei Zhong Bing Ji Jiu Yi Xue. 2019;31(8):1008-1012  94. Jagan S, Park T, Papathanassoglou E. Effects of massage on outcomes of adult intensive care unit patients: a systematic review. Nurs Crit Care. 2019;24(6):414-429  95. 陶朝辉, 罗丹, 廖力, 陈敬芳. 睡眠干预对成人危重患者谵妄预防效果的系统评价. 中国护理管理. 2019;19(12):1841-1849  96. 李佳, 丁佳蓉, 冯波, 许艳. 光照疗法预防ICU患者谵妄的系统评价. 护理学杂志. 2019;34(01):91-94  97. Thrane SE, Hsieh K, Donahue P et al. Could complementary health approaches improve the symptom experience and outcomes of critically ill adults? A systematic review of randomized controlled trials. Complement Ther Med. 2019;47(102166)  98. Groves RL. Increasing Light Exposure for the Prevention of Delirium: A Systematic Review. Dimens Crit Care Nurs. 2019;38(2):96-107  99. Kang J, Lee M, Ko H et al. Effect of nonpharmacological interventions for the prevention of delirium in the intensive care unit: A systematic review and meta-analysis. J Crit Care. 2018;48:372-384  100. 郑咏坤. 经鼻高流量预给氧应用于ICU气管插管的效果：系统评价与Meta分析. 福建医科大学. 2018.  101. 常瑞, 杨辉. 最小化镇静策略在ICU机械通气患者中应用效果的Meta分析. 中国卫生产业. 2018;15(6):174-176  102. 杨丽, 李敏, 李亚男, 田金徽. 6种强化护理干预措施对ICU住院病人谵妄影响效果的网状Meta分析. 循证护理. 2018;4(5):394-399  103. 刘培培, 李津, 王晓冉, 王圣巍. ABCDE集束化干预在ICU机械通气病人中应用效果的Meta分析. 循证护理. 2018;4(6):481-487  104. Locihova H, Axmann K, Padysakova H, Fejfar J. Effect of the use of earplugs and eye mask on the quality of sleep in intensive care patients: a systematic review. J Sleep Res. 2018;27(e126073)  105. 查丽玲, 周松, 江榕, 王建宁. 经鼻高流量氧疗在成人气管插管患者拔管后应用效果的Meta分析. 中华现代护理杂志. 2017;23(23):3011-3015  106. 姜文静, 张玲, 李娟. ABCDE集束化镇痛镇静策略在ICU机械通气患者中应用效果的Meta分析. 护士进修杂志. 2017;32(8):687-690  107. 黄华平, 陈斌, 王海燕. 经鼻导管高流量吸氧在危重患者气管插管拔除后应用效果的Meta分析. 护理管理杂志. 2017;17(6):399-401  108. Azizpour M, Moosazadeh M, Esmaeili R. Use of physical restraints in intensive care unit: A systematic review study. Acta Medica Mediterr. 2017;33(1):129-136  109. Flannery AH, Oyler DR, Weinhouse GL. The Impact of Interventions to Improve Sleep on Delirium in the ICU: A Systematic Review and Research Framework. Crit Care Med. 2016;44(12):2231-2240  110. Kiwanuka F, Shayan SJ, Tolulope AA. Barriers to patient and family-centred care in adult intensive care units: A systematic review. Nurs Open. 2019;6(3):676-684.  111. 宋春梅,吴娟.ICU护士对谵妄病人管理经验质性研究的Meta整合.护理研究,2020,34(04):613-620.  112. 陈俊杉,余金甜,刘晶涛,张爱琴.ICU护士护理谵妄患者真实体验研究的Meta整合.解放军护理杂志,2019,36(11):19-22.  113. Qi Z, Yang S, Qu J, et al. Effects of nurse-led sedation protocols on mechanically ventilated intensive care adults: A systematic review and meta-analysis. Aust Crit Care. 2021;34(3):278-286. |
| **Meeting abstracts (n=38)** |
| 1. Johnson D, Maylin E, Hair C et al. A systematic review of stroke, delirium and allied health interventions; an exercise in futility. European Stroke Journal. 2018;3(1):355  2. Kyeremanteng K, Bhardwaj K, Chaudhuri D et al. Icu delirium, clinical outcomes, and cost: Systematic review and metaanalysis. Crit Care Med. 2018;46:235  3. Fiest K, Leigh JP, Farris M et al. Incidence and prevalence of delirium subtypes in the ICU: A systematic review and meta-analysis. Crit Care Med. 2016;44(12):200  4. Rafay H, Honarmand K, Le JB et al. Systematic review of risk factors associated with disrupted sleep in critically ill patients. Am J Resp Crit Care. 2019;199:9  5. Honarmand K, Le J, Mohan S, Rochwerg B, Bosma KJ. Risk factors for sleep disruption in critically ill patients: A systematic review of the literature. Canadian Journal of Anesthesia. 2018;65:S101-S103  6. Talbot S, McGrath BA. Interventions to improve communication in mechanically ventilated patients: a review. Brit J Anaesth. 2019;122(3):e49-e50  7. Ding N, Zhigang Z, Caiyun Z et al. The four weaning index as predictors of mechanical ventilated patients: A systematic review and diagnostic meta-analysis. Intensive Care Medicine Experimental. 2019;7(Suppl 3):446  8. Turton P, Welters I. A survey of current attitudes to diaphragm ultrasound in the prediction of successful respiratory weaning. Journal of the Intensive Care Society. 2019;20(2):191  9. Spiegel R, Stocker S, Opic P et al. Can artificial intelligence help to predict ventilation days? A systematic review. Crit Care Med. 2019;47(1):540  10. Glassford N, Casamento A, Beebee B, Bellomo R. Prediction of tracheostomy in critically ill patients: A systematic review. Journal of the Intensive Care Society. 2018;19(2):148-149  11. Nkwonta C. Does timing of tracheostomy influence outcomes in the critically ill? Canadian Journal of Respiratory Therapy. 2017;53(3):49  12. Ding N, Zhigang Z, Caiyun Z et al. Effects of different early mobilization initiative time on the ICU mechanical ventilated patients: a network meta-analysis. Intensive Care Medicine Experimental. 2019;7(Suppl 3):273  13. Chaudhuri D, Granton D, Wang D et al. High flow nasal cannula use compared to conventional oxygen therapy in the peri-intubation period: A systematic review and meta-analysis. Intensive Care Medicine Experimental. 2019;7(Suppl 3):273  14. Higgins S, Erdogan M, Coles J, Green R. Early mobilization of trauma patients admitted to intensive care units: A systematic review and meta-analysis. Can J Emerg Med. 2019;21:S84  15. Bartholomew A, Simms K, Seenan C. Does neurally adjusted ventilatory assist improve outcomes for critically ill adults during weaning from mechanical ventilation? A systematic review. Physiotherapy. 2019;105:e50-e51  16. Roque VL, Jesse Lomanta JM, Velasquez JR, Maghuyop N. Proportional assist ventilation as a mode of weaning in adult patients requiring mechanical ventilation: A meta-analysis. Respirology. 2018;23:256-257  17. Chaudhuri D, Herritt B, Kyeremanteng K. Cost analysis of early vs late trachestomy in intensive care settings. Canadian Journal of Anesthesia. 2018;65:S33  18. Sethi J, Angl A, Ali M, Mohananey D. Role of high flow oxygen therapy in adult patients after extubation: A systematic review and meta-analysis. Chest. 2017;152(4):A223  19. Tirupakuzhi Vijayaraghavan BK, Hamed S, Jain A et al. Evidence supporting the clinical uses of proportional assist ventilation: A systematic review and meta-analysis. Intensive Care Medicine Experimental. 2017;5(Suppl 2):261  20. Thamjamrassri T, Watcharotayangul J, Numthavaj P, Kongsareepong S. High flow nasal cannula prevent reintubation in post-extubated critically ill patients: A systematic review and meta-analysis. Crit Care. 2017;21(Suppl 1):16  21. Neuschwander A, Vincent L, Vibol C et al. Comparative performance of different automated weaning modes: A network meta-analysis. Ann Intensive Care. 2017;7(1):195  22. Aldawood B, Al-Rajhi A, Shahin J. High-flow nasal cannula in post-extubation patients: A meta-analysis. Crit Care Med. 2016;44(12):322  23. Pan L, Guo Y. Effect of early versus late tracheotomy in critically ill patients: A systematic review and meta-analysis. Chest. 2016;149(4):A165  24. Hakim R, Watanabe Tejada LC, Sukhal S, Tulaimat A. A systematic review of the criteria for endotracheal intubation for mechanical ventilation in randomized trials. Am J Resp Crit Care. 2018;197:A5134  25. Keller JM, Claar D, Chu DC et al. Mechanical ventilation training during graduate medical education. Am J Resp Crit Care. 2019;199(9): A4781  26. Zayed Y, Rashdan L, Chintalapati S et al. Effects of neuromuscular electrical stimulation in critically ill patients: A meta-analysis of randomized controlled trials. Am J Resp Crit Care. 2019;199(9):A4116  27. Chang J, Schwab K, To A et al. The use of actigraphy to evaluate physical activity in the intensive care unit: A systematic review. Am J Resp Crit Care. 2018;197:A1834  28. Medrinal C, Combret Y, Hilfker R et al. Consequences of ICU acquired weakness: A systematic review and meta-analysis. Ann Intensive Care. 2020;10(Suppl 1):2  29. Saunders R, Davis JA, Bosma KJ. PRS30 PRAGMATIC META-ANALYSIS OF PROPORTIONAL ASSIST VENTILATION+ VERSUS PRESSURE SUPPORT VENTILATION AND ITS IMPACT ON COST EFFECTIVENESS. Value Health. 2019;22:S878  30. Kareemi H, Chaudhuri D, Herritt B et al. Automated versus nonautomated ventilation weaning in the icu: A systematic review and cost analysis. Crit Care Med. 2018;46:572  31. Danielis M, Mattiussi E, Vidoni M, Venuti L, Palese A. Patients' experience regarding night time and sleep deprivation in ICU: Results from a meta synthesis. Intensive Care Medicine Experimental. 2018;6(Suppl 2):0719  32. Grosso M, Domingos R, Serápio F, Alves K, Castro C. Most used pain assessment scales in the unconscious critical patient: A systematic review. Ann Med. 2019;51:S208-S209  33. Denny N, Ashby N. The use of the confusion assessment method (CAM-ICU) to reduce incidence of delirium in intensive care and improve outcomes: A systematic review. Journal of the Intensive Care Society. 2018;19(2):59  34. Yuchen W, Nannan D, Biantong J, Zhigang Z. A meta-analysis of the effect of threshold inspiratory muscle training on respiratory muscle function. Intensive Care Medicine Experimental. 2019;7(Suppl 3):000611  35. Yuchen W, Biantong J, Nannan D, Zhigang Z. Effects of different visiting policies on ICU syndrome: A Systematic review and Meta-analysis. Intensive Care Medicine Experimental. 2019;7(Suppl 3):000606  36. Wei S, Xiaocong H, Wenhao X. Advances in management of delirium in critically ill adults: A systematic review and meta-analysis. Shock. 2019;51(6):81  37. AlQahtani RM, Newman A, Moragno J, Fox-Robichaud AE. Noise control in acute care units: A systematic review and meta-analysis of randomized controlled trials. Am J Resp Crit Care. 2017;195:A1074  38. Bibas L, Peretz-Larochelle M, Goldfarb MJ, Luk A, Englesakis M, Detsky ME, et al. Interventions to improve surrogate decision-making in intensive care units: A systematic review and meta-analysis of randomized controlled trials. Can J Anesth, 2019. 66(1): p. S69-S70. |
| **Not related (n=23)** |
| 1. Oczkowski SJ, Chung HO, Hanvey L, Mbuagbaw L, You JJ. Communication tools for end-of-life decision-making in the intensive care unit: a systematic review and meta-analysis. Crit Care. 2016;20:97  2. McIntyre M, Doeltgen S, Dalton N, Koppa M, Chimunda T. Post-extubation dysphagia incidence in critically ill patients: A systematic review and meta-analysis. Aust Crit Care. 2021;34(1):67-75.  3. McIntyre M, Chimunda T, Koppa M et al. Risk Factors for Postextubation Dysphagia: A Systematic Review and Meta-analysis. Laryngoscope. 2020;10.1002/lary.29311  4. Sánchez SL, Goñi BI, Ruiz GP, Díaz AJ, Leal CC. Acquired neuromuscular dysfunction in the intensive care unit. Enferm Intensiva. 2018;29(3):128-137  5. Guo L, Wang W, Zhao N et al. Mechanical ventilation strategies for intensive care unit patients without acute lung injury or acute respiratory distress syndrome: a systematic review and network meta-analysis. Crit Care. 2016;20(1):226  6. Borsellino B, Schultz MJ, Gama DAM, Robba C, Bilotta F. Mechanical ventilation in neurocritical care patients: a systematic literature review. Expert Rev Respir Med. 2016;10(10):1123-32  7. Yang T, Li Z, Jiang L, Wang Y, Xi X. Risk factors for intensive care unit-acquired weakness: A systematic review and meta-analysis. Acta Neurol Scand. 2018;138(2):104-114  8. Manias E, Bucknall T, Wickramasinghe N et al. Patient and family engagement in communicating with electronic medical records in hospitals: A systematic review. Int J Med Inform. 2020;134(104036)  9. Delaney RK, Sisco-Taylor B, Fagerlin A, Weir P, Ozanne EM. A systematic review of intensive outpatient care programs for high-need, high-cost patients. Transl Behav Med. 2020;10(5):1187-1199  10. Schofield Robinson OJ, Lewis SR, Smith AF, McPeake J, Alderson P. Follow‐up services for improving long‐term outcomes in intensive care unit (ICU) survivors. Cochrane Database Syst Rev. 2018;11(11):CD012701.  11. Honarmand K, Lalli RS, Priestap F et al. Natural History of Cognitive Impairment in Critical Illness Survivors. A Systematic Review. Am J Respir Crit Care Med. 2020;202(2):193-201  12. 姚丽, 丁楠楠, 杨丽平 et al. 重症患者转出ICU后认知损害危险因素的Meta分析. 中国护理管理. 2018;18(12):1634-1643  13. Efstathiou N, Vanderspank-Wright B, Vandyk A et al. Terminal withdrawal of mechanical ventilation in adult intensive care units: A systematic review and narrative synthesis of perceptions, experiences and practices. Palliat Med. 2020;34(9):1140-1164  14. Hosie A, Siddiqi N, Featherstone I et al. Inclusion, characteristics and outcomes of people requiring palliative care in studies of non-pharmacological interventions for delirium: A systematic review. Palliat Med. 2019;33(8):878-899  15. Pisani L, Hill NS, Pacilli A, Polastri M, Nava S. Management of Dyspnea in the Terminally Ill. Chest. 2018;154(4):925-934  16. Chen C, Michaels J, Meeker MA. End-of-Life Care in the Intensive Care Unit: A Systematic Review of Family Perceptions. J Pain Symptom Manag. 2018;56(6):e127  17. Owen L. Advance care planning: what do patients want? Eur Geriatr Med. 2018;9:S323  18. Pignatiello G, Hickman RL, Hetland B. End-of-Life Decision Support in the ICU: Where Are We Now? Western J Nurs Res. 2018;40(1):84-120  19. Lord L, Clark-Carter D, Grove A. The effectiveness of communication-skills training interventions in end-of-life noncancer care in acute hospital-based services: A systematic review. Palliat Support Care. 2016;14(4):433-444  20. Dupuis S, Brindamour D, Karzon S et al. A systematic review of interventions to facilitate extubation in patients difficult-to-wean due to delirium, agitation, or anxiety and a meta-analysis of the effect of dexmedetomidine. Can J Anaesth. 2019;66(3):318-327  21. Casamento AJ, Bebee B, Glassford NJ, Bellomo R. Prediction of tracheostomy in critically ill trauma patients: a systematic review. Crit Care Resusc. 2018;20(4):258-267.  22. 栾玲芹,虎磐,刘超,李东美,高玲,王国旗,蔡玉琰,毛智,周飞虎. 间歇声门下吸引预防呼吸机相关性肺炎的Meta分析和试验序贯分析.中华医院感染学杂志,2017,27(18):4131-4135.  23. Nedergaard HK, Jensen HI, Toft P. Interventions to reduce cognitive impairments following critical illness: a topical systematic review. Acta Anaesthesiol Scand. 2017;61(2):135-148. |
| **Not a systematic review (n=15)** |
| 1. Viderman D, Brotfain E, Bilotta F, Zhumadilov A. Risk Factors and Mechanisms of Postoperative Delirium After Intracranial Neurosurgical Procedures. Asian J Anesthesiol. 2020;58(1):5-13.  2. Cosentino C, Fama M, Foà C et al. Unplanned Extubations in Intensive Care Unit: evidences for risk factors. A literature review. Acta Biomed. 2017;88(5S):55-65  3. Grimm J. Sleep Deprivation in the Intensive Care Patient. Crit Care Nurse. 2020;40(2):e16-e24  4. Richards KC, Wang Y, Jun J, Ye L. A Systematic Review of Sleep Measurement in Critically Ill Patients. Front Neurol. 2020;11:542529.  5. Miller EK, Beavers LG, Mori B et al. Assessing the Clinical Competence of Health Care Professionals Who Perform Airway Suctioning in Adults. Resp Care. 2019;64(7):844-854  6. Stieff KV, Lim F, Chen L. Factors influencing weaning older adults from mechanical ventilation: An integrative review. Critical Care Nursing Quarterly. 2017;40(2):165-177  7. Guia M, Ciobanu LD, Sreedharan JK et al. The role of non-invasive ventilation in weaning and decannulating critically ill patients with tracheostomy: A narrative review of the literature. Pulmonology. 2021;27(1):43-51.  8. Waterfield D, Barnason S. Use of PADIS Assessment Tools by Critical Care Nurses: An Integrative Review. West J Nurs Res. 2021;43(9):843-858.  9. Sedighie L, Bolourchifard F, Rassouli M, Sanee N. Evaluating the implementation of pain management templates in adult intensive care units: A systematic review. Anaesthesia, Pain and Intensive Care. 2020;24(2):151-167  10. Gosselin É, Richard-Lalonde M. Role of Family Members in Pain Management in Adult Critical Care. AACN Adv Crit Care. 2019;30(4):398-410  11. Azevedo-Santos IF, DeSantana JM. Pain measurement techniques: Spotlight on mechanically ventilated patients. J Pain Res. 2018;11:2969-2980  12. 刘苗, 罗健, 黄海燕 et al. 非药物干预预防ICU获得性谵妄相关系统评价的再评价. 护理学杂志. 2020;35(01):77-82  13. Mitchell M, Bernie C, Newall F, Williams K. Simulation-based education for teaching aggression management skills to health care providers in the acute health care setting: a systematic review protocol. yst Rev. 2020;9(1):208.  14. Da Cruz Pessoa LS, de Oliva Menezes TM, Pereira Gomes N et al. NURSING CARE FOR ELDERLY PATIENTS WITH DELIRIUM IN INTENSIVE CARE UNITS. Journal of Nursing UFPE. 2019;13:372-378  15. Zegers M, Hesselink G, Geense W, Vincent C, Wollersheim H. Evidence-based interventions to reduce adverse events in hospitals: a systematic review of systematic reviews. BMJ Open. 2016;6(9):e012555. |
| **Non-ICU (n=10)** |
| 1. MohammadiGorji S, Bosch SJ, Valipoor S, De Portu G. Investigating the Impact of Healthcare Environmental Design on Staff Security: A Systematic Review. HERD. 2021;14(1):251-272.  2. Gaynes BN, Brown CL, Lux LJ et al. Preventing and De-escalating Aggressive Behavior Among Adult Psychiatric Patients: A Systematic Review of the Evidence. Psychiatr Serv. 2017;68(8):819-831  3. Lim E, Wynaden D, Heslop K. Recovery-focussed care: How it can be utilized to reduce aggression in the acute mental health setting. Int J Ment Health Nu. 2017;26(5):445-460  4. McFeeters S, Pront L, Cuthbertson L, King L. Massage, a complementary therapy effectively promoting the health and well-being of older people in residential care settings: a review of the literature. Int J Older People N. 2016;11(4):266-283  5. Watt JA, Goodarzi Z, Veroniki AA et al. Comparative Efficacy of Interventions for Aggressive and Agitated Behaviors in Dementia: A Systematic Review and Network Meta-analysis. Ann Intern Med. 2019;171(9):633-642  6. Bridges J, Collins P, Flatley M, Hope J, Young A. Older people's experiences in acute care settings: Systematic review and synthesis of qualitative studies. Int J Nurs Stud. 2020;102:103469.  7. Shrestha P, Fick DM. Family caregiver's experience of caring for an older adult with delirium: A systematic review. Int J Older People Nurs. 2020;15(4):e12321.  8. Brookes C, Galvin R. Diagnostic accuracy of the 4AT rapid assessment test for delirium: A systematic review and metaanalysis. Age Ageing. 2018;47:260  9. Ekeozor CU, Jeyaruban D, Lasserson D. Where should patients with or at risk of delirium be treated in an acute care system? Comparing the rates of delirium in patients receiving usual care versus alternative care: a systematic review and meta-analysis. Int J Clin Pract. 2021; 75:e13859.  10. Oh ES, Fong TG, Hshieh TT, Inouye SK. Delirium in older persons: Advances in diagnosis and treatment. JAMA. 2017;318(12):1161-1174 |
| **Without outcomes (n=33)** |
| 1. Burke D, Gorman E, Stokes D, Lennon O. An evaluation of neuromuscular electrical stimulation in critical care using the ICF framework: a systematic review and meta-analysis. Clin Respir J. 2016;10(4):407-20  2. AbuNurah HY, Russell DW, Lowman JD. The validity of surface EMG of extra-diaphragmatic muscles in assessing respiratory responses during mechanical ventilation: A systematic review. Pulmonology. 2020;26(6):378-385.  3. 胡燕, 李冬英, 胡晓莹, 肖伽, 李瑛. 早期活动对机械通气病人ICU获得性肌无力影响的累计Meta分析及试验序贯分析. 护理研究. 2020;34(18):3190-3196  4. Anekwe DE, Biswas S, Bussieres A, Spahija J. Early rehabilitation reduces the likelihood of developing intensive care unit-acquired weakness: a systematic review and meta-analysis. Physiotherapy. 2020;107:1-10  5. 赵贵美, 焦琳琳, 杨桂华. 早期循序渐进运动对ICU患者获得性衰弱影响的Meta分析. 中华护理杂志. 2017;52(02):177-181  6. Strametz R, Bergold MN, Weberschock T. Laryngeal mask airway versus endotracheal tube for percutaneous dilatational tracheostomy in critically ill adults. Cochrane Db Syst Rev. 2018;11(11):CD009901.  7. 王金娥, 顾芳臣, 林征. 两种声门下吸引方式对机械通气患者呼吸机相关性肺炎及气管黏膜损伤影响的meta分析. 南京医科大学学报(自然科学版). 2020;40(11):1645-1653  8. Pettenuzzo T, Aoyama H, Englesakis M, Tomlinson G, Fan E. Effect of Neurally Adjusted Ventilatory Assist on Patient-Ventilator Interaction in Mechanically Ventilated Adults: A Systematic Review and Meta-Analysis. Crit Care Med. 2019;47(7):e602-e609.  9. Chen J, Yu J, Zhang A. Delirium risk prediction models for intensive care unit patients: A systematic review. Intensive Crit Care Nurs. 2020;60:102880.  10. Coombs MA, Statton S, Endacott CV, Endacott R. Factors influencing family member perspectives on safety in the intensive care unit: a systematic review. Int J Qual Health Care. 2020;32(9):625-638.  11. Tylee MJ, Rubenfeld GD, Wijeysundera D, Sklar MC, Hussain S, Adhikari NKJ. Anesthesiologist to Patient Communication: A Systematic Review. JAMA Netw Open. 2020;3(11):e2023503.  12. Ahn JW, Jang HY, Son YJ. Critical care nurses' communication challenges during handovers: A systematic review and qualitative meta-synthesis. J Nurs Manag. 2021;29(4):623-634.  13. Deschamps J, Andersen SK, Webber J, et al. Brain natriuretic peptide to predict successful liberation from mechanical ventilation in critically ill patients: a systematic review and meta-analysis. Crit Care. 2020;24(1):213.  14. Sato R, Hasegawa D, Hamahata NT, et al. The predictive value of airway occlusion pressure at 100 msec (P0.1) on successful weaning from mechanical ventilation: A systematic review and meta-analysis. J Crit Care. 2021;63:124-132.  15. Zambon M, Greco M, Bocchino S, Cabrini L, Beccaria PF, Zangrillo A. Assessment of diaphragmatic dysfunction in the critically ill patient with ultrasound: a systematic review. Intensive Care Med. 2017;43(1):29-38.  16. Medrinal C, Combret Y, Hilfiker R, et al. ICU outcomes can be predicted by noninvasive muscle evaluation: a meta-analysis. Eur Respir J. 2020;56(4):1902482.  17. Kuriyama A, Jackson JL, Kamei J. Performance of the cuff leak test in adults in predicting post-extubation airway complications: a systematic review and meta-analysis. Crit Care. 2020;24(1):640.  18. Ghauri SK, Javaeed A, Mustafa KJ, Khan AS. Predictors of prolonged mechanical ventilation in patients admitted to intensive care units: A systematic review. Int J Health Sci (Qassim). 2019;13(6):31-38.  19. Jordan J, Rose L, Dainty KN, Noyes J, Blackwood B. Factors that impact on the use of mechanical ventilation weaning protocols in critically ill adults and children: a qualitative evidence-synthesis. Cochrane Database Syst Rev. 2016;10(10):CD011812.  20. Danielis M, Povoli A, Mattiussi E, Palese A. Understanding patients' experiences of being mechanically ventilated in the Intensive Care Unit: Findings from a meta-synthesis and meta-summary. J Clin Nurs. 2020;29(13-14):2107-2124.  21. Carruthers H, Gomersall T, Astin F. The work undertaken by mechanically ventilated patients in Intensive Care: A qualitative meta-ethnography of survivors' experiences. Int J Nurs Stud. 2018;86:60-73.  22. Parry SM, Knight LD, Connolly B, et al. Factors influencing physical activity and rehabilitation in survivors of critical illness: a systematic review of quantitative and qualitative studies. Intensive Care Med. 2017;43(4):531-542.  23. Ding N, Zhang Z, Zhang C, et al. What is the optimum time for initiation of early mobilization in mechanically ventilated patients? A network meta-analysis. PLoS One. 2019;14(10):e0223151.  24. Chen TJ, Chung YW, Chang HR, et al. Diagnostic accuracy of the CAM-ICU and ICDSC in detecting intensive care unit delirium: A bivariate meta-analysis. Int J Nurs Stud. 2021;113:103782.  25. Ho MH, Montgomery A, Traynor V, et al. Diagnostic Performance of Delirium Assessment Tools in Critically Ill Patients: A Systematic Review and Meta-Analysis. Worldviews Evid Based Nurs. 2020;17(4):301-310.  26. Patel MB, Bednarik J, Lee P, et al. Delirium Monitoring in Neurocritically Ill Patients: A Systematic Review. Crit Care Med. 2018;46(11):1832-1841  27. Jeong E, Park J, Lee J. Diagnostic test accuracy of the Nursing Delirium Screening Scale: A systematic review and meta-analysis. J Adv Nurs. 2020;76(10):2510-2521.  28. van Velthuijsen EL, Zwakhalen SM, Warnier RM, Mulder WJ, Verhey FR, Kempen GI. Psychometric properties and feasibility of instruments for the detection of delirium in older hospitalized patients: a systematic review. Int J Geriatr Psychiatry. 2016;31(9):974-989.  29. Ho MH, Chen KH, Montayre J, et al. Diagnostic test accuracy meta-analysis of PRE-DELIRIC (PREdiction of DELIRium in ICu patients): A delirium prediction model in intensive care practice. Intensive Crit Care Nurs. 2020;57:102784.  30. Chen X, Lao Y, Zhang Y, Qiao L, Zhuang Y. Risk predictive models for delirium in the intensive care unit: a systematic review and meta-analysis. Ann Palliat Med. 2021;10(2):1467.  31. Gaete Ortega D, Papathanassoglou E, Norris CM. The lived experience of delirium in intensive care unit patients: A meta-ethnography. Aust Crit Care. 2020;33(2):193-202.  32. Mattiussi E, Danielis M, Venuti L, Vidoni M, Palese A. Sleep deprivation determinants as perceived by intensive care unit patients: Findings from a systematic review, meta-summary and meta-synthesis. Intensive Crit Care Nurs. 2019;53:43-53.  33. Ai ZP, Gao XL, Zhao XL. Factors associated with unplanned extubation in the Intensive Care Unit for adult patients: A systematic review and meta-analysis. Intensive Crit Care Nurs (2018) 47: 62-68. |
| **Repeated systematic reviews (n=4)** |
| 1. Rood P, Huisman-de Waal G, Vermeulen H et al. Exploring the role of organizational factors to variations in incidence of delirium in ICU patients. Intensive Care Medicine Experimental. 2017;5(2):0652  2. Aitken LM, Bucknall T, Kent B et al. Sedation protocols to reduce duration of mechanical ventilation in the ICU: a Cochrane Systematic Review. J Adv Nurs. 2016;72(2):261-272  3. Liu M, Luo J, Zhou J, Zhu X. Intervention effect of neuromuscular electrical stimulation on ICU acquired weakness: A meta-analysis. Int J Nurs Sci. 2020;7(2):228-237.  4. Leitch J, Gill R, Poser K, McKeown S, Galvin I. Humanization of critical care-psychological effects on healthcare professionals & relatives: A systematic review. Can J Anesth. 2019;66(1):S62-S63 |
| **Non-English or Chinese (n=1)** |
| 1. García-Sánchez A, Barbero E, Pintado B et al. Diaphragmatic dysfunction evaluated by ultrasound as a predictor of extubation failure: systematic review and meta-analysis. Open Respiratory Archives. 2020;2(4):267-277 |

**Table 3 AMSTAR 2 quality appraisal scores**

| **Item No** | 1 | **2** | 3 | **4** | 5 | 6 | **7** | 8 | **9** | 10 | **11** | 12 | **13** | 14 | **15** | 16 | **Overall rating** |
| --- | --- | --- | --- | --- | --- | --- | --- | --- | --- | --- | --- | --- | --- | --- | --- | --- | --- |
| Franks et al 2021 | Y | N | N | PY | Y | Y | Y | PY | Y | Y | Y | Y | N | Y | Y | Y | Critically low |
| Nassar et al 2018 | Y | Y | N | PY | Y | Y | N | PY | PY | N | Y | Y | Y | Y | Y | Y | Low |
| Galvin et al 2018 | Y | Y | N | PY | Y | Y | Y | Y | Y | N | NM | NM | Y | Y | NM | Y | Moderate |
| Lee et al 2019 | Y | Y | N | PY | Y | Y | Y | Y | Y | N | Y | N | N | Y | Y | Y | Low |
| Bibas et al 2019 | Y | Y | N | PY | Y | Y | N | Y | Y | N | Y | N | Y | Y | Y | Y | Low |
| Umbrello et al 2019 | Y | Y | N | PY | Y | Y | Y | PY | Y | N | NM | NM | N | N | NM | Y | Low |
| Geense et al 2019 | Y | Y | N | PY | Y | Y | N | PY | Y | N | Y | N | N | N | Y | Y | Critically low |
| Kynoch et al 2016 | Y | Y | N | PY | Y | Y | PY | PY | PY | N | NM | NM | N | Y | NM | Y | Low |
| Goldfarb et al 2017 | Y | N | N | PY | Y | Y | N | PY | PY | N | Y | N | Y | N | Y | Y | Critically low |
| Carruthers et al 2017 | Y | Y | Y | PY | Y | Y | N | Y | Y | N | NM | NM | Y | Y | NM | Y | Low |
| Michalsen et al 2019 | Y | PY | N | PY | Y | Y | N | PY | Y | N | NM | NM | Y | Y | NM | Y | Low |
| Ai et al 2018 | Y | N | N | PY | Y | Y | N | PY | Y | N | Y | N | Y | N | Y | Y | Critically low |
| Ou-Yang et al 2020 | Y | Y | N | PY | Y | Y | N | Y | Y | N | Y | N | Y | Y | Y | Y | Low |
| Kataoka et al 2018 | Y | Y | N | PY | Y | Y | Y | Y | Y | N | Y | N | N | Y | Y | Y | Low |
| Neuschwander et al 2021 | Y | Y | N | PY | Y | Y | N | PY | Y | N | Y | Y | N | N | Y | Y | Critically low |
| Hirzallah et al 2019 | Y | N | N | PY | Y | Y | Y | Y | PY | N | Y | N | N | Y | Y | Y | Critically low |
| Pileggi et al 2018 | Y | N | N | PY | Y | Y | Y | Y | Y | N | Y | Y | Y | Y | Y | Y | Low |
| Rose et al 2017 | Y | Y | Y | Y | Y | Y | Y | Y | Y | Y | NM | NM | Y | Y | NM | Y | High |
| Wang et al 2019 | Y | N | N | PY | Y | Y | N | PY | PY | N | Y | N | Y | Y | Y | Y | Critically low |
| McCredie et al 2017 | Y | Y | N | PY | Y | Y | Y | Y | Y | N | Y | Y | N | Y | Y | Y | Low |
| de Franca et al 2020 | Y | N | N | PY | Y | Y | N | Y | Y | N | Y | N | Y | Y | Y | Y | Critically low |
| Granton et al 2020 | Y | Y | N | PY | Y | Y | N | Y | Y | N | Y | N | Y | Y | Y | Y | Low |
| Chaudhuri et al 2020 | Y | Y | N | PY | Y | Y | N | Y | Y | N | Y | N | Y | Y | Y | Y | Low |
| Zhu et al 2019 | Y | N | N | PY | Y | Y | Y | Y | Y | N | Y | Y | Y | Y | Y | Y | Low |
| Huang et al 2018 | Y | Y | N | PY | Y | Y | Y | Y | Y | N | Y | N | N | Y | Y | Y | Low |
| Lewis et al 2021 | Y | Y | Y | Y | Y | Y | Y | Y | Y | Y | Y | Y | Y | Y | Y | Y | High |
| Zhao et al 2017 | Y | Y | N | PY | Y | Y | Y | Y | Y | N | Y | N | Y | Y | Y | Y | Moderate |
| Waldauf et al 2020 | Y | Y | N | PY | Y | Y | N | Y | Y | N | Y | Y | N | Y | Y | Y | Critically low |
| Nydahl et al 2017 | Y | Y | N | PY | Y | Y | N | PY | PY | N | Y | N | N | Y | Y | Y | Critically low |
| Zang et al 2020 | Y | N | N | PY | Y | Y | Y | PY | Y | N | Y | N | N | Y | Y | Y | Critically low |
| Zhang et al 2019 | Y | N | N | PY | Y | Y | N | PY | Y | N | Y | N | N | N | Y | Y | Critically low |
| Doiron et al 2018 | Y | Y | N | Y | Y | Y | Y | Y | Y | Y | Y | Y | Y | Y | Y | Y | High |
| Fuke et al 2018 | Y | Y | N | PY | Y | Y | Y | PY | Y | N | Y | Y | Y | Y | Y | Y | Moderate |
| Worraphan et al 2020 | Y | Y | N | PY | Y | Y | N | PY | Y | N | Y | N | Y | N | Y | Y | Low |
| Wu et al 2019 | Y | N | N | PY | Y | Y | N | PY | Y | N | Y | N | Y | N | Y | Y | Critically low |
| Vorona et al 2018 | Y | N | N | PY | Y | Y | N | PY | PY | N | Y | Y | Y | Y | Y | Y | Critically low |
| Liang et al 2021 | Y | Y | N | PY | Y | Y | N | PY | Y | N | Y | Y | N | N | Y | Y | Critically low |
| Bannon et al 2019 | Y | Y | N | PY | Y | Y | Y | Y | Y | N | Y | Y | Y | Y | Y | Y | Moderate |
| León-Salas et al 2020 | Y | Y | N | PY | Y | Y | N | PY | Y | N | Y | Y | N | N | Y | Y | Critically low |
| Herling et al 2018 | Y | Y | N | Y | Y | Y | Y | Y | Y | Y | Y | Y | Y | Y | Y | Y | High |
| Lu et al 2019 | Y | N | N | PY | Y | Y | Y | Y | Y | N | Y | N | N | Y | Y | Y | Critically low |
| Litton et al 2016 | Y | N | N | PY | Y | Y | N | PY | PY | N | Y | Y | Y | N | Y | Y | Critically low |
| Shetty et al 2018 | Y | Y | N | Y | Y | Y | Y | Y | Y | Y | Y | Y | Y | Y | Y | Y | High |
| Aitken et al 2018 | Y | Y | N | Y | Y | Y | Y | Y | Y | Y | Y | Y | Y | Y | Y | Y | High |
| Nassar et al 2016 | Y | Y | N | PY | Y | Y | Y | PY | PY | N | Y | Y | Y | N | Y | Y | Moderate |
| Stephens et al 2018 | Y | Y | N | PY | Y | Y | N | PY | PY | N | Y | N | N | Y | Y | Y | Critically low |
| Long et al 2020 | Y | Y | N | PY | Y | Y | Y | PY | Y | N | Y | N | N | Y | Y | Y | Critically low |
| Hadjibalassi et al 2018 | Y | PY | N | PY | Y | Y | N | PY | PY | N | NM | NM | Y | Y | NM | Y | Low |

Abbreviation: AMSTAR 2: A MeaSurement Tool to Assess systematic Reviews; Y: Yes; N: No; PY: Partial Yes; NM: No meta-analysis conducted

**Table 4 Citation matrix**

- Multicomponent interventions involving healthcare professionals’ education (not calculated)
- Family engagement/support

|  | Nassar et al 2018 | Galvin et al 2018 | Kynoch et al 2016 | Geense et al 2019 | Lee et al 2019 |
| --- | --- | --- | --- | --- | --- |
| Baharoon et al., 2017 | × |  |  |  |  |
| Chapman et al., 2016 | × |  |  |  |  |
| Eghbali-Babadi et al., 2017 | × |  |  |  |  |
| Fumagalli et al., 2013 | × |  |  |  |  |
| Fumagalli et al., 2006 | × |  |  |  |  |
| Giannini et al., 2013 | × | × |  |  |  |
| Malacarne et al., 2011 | × |  |  |  |  |
| Mitchell et al., 2017 | × |  |  |  |  |
| Novaes et al., 2001 | × |  |  |  |  |
| Ramsey et al., 1999 | × |  | × |  |  |
| Roland et al., 2001 | × |  |  |  |  |
| Rosa et al., 2017 | × |  |  |  |  |
| Schnell et al., 2013 | × |  |  |  |  |
| Soares et al., 2017 | × |  |  |  |  |
| Whitcomb et al., 2010 | × |  |  |  |  |
| Henneman et al., 1992 | × |  | × |  |  |
| Robinson et al., 1998 |  | × |  |  |  |
| Kloos et al., 2008 |  | × | × |  |  |
| Garrouste et al., 2010 |  | × |  |  |  |
| Skoog et al., 2016 |  | × |  |  |  |
| Quenot et al., 2012 |  | × |  |  |  |
| Holl et al., 1992 |  | × |  |  |  |
| Orlen et al., 1993 |  | × |  |  |  |
| Rodriguez et al., 2003 |  | × |  |  |  |
| Prichard et al., 2015 |  | × |  |  |  |
| Leske et al., 2017 |  | × |  |  |  |
| Pasquale et al., 2010 |  | × |  |  |  |
| Azoulay et al., 2002 |  |  | × |  |  |
| Kirchhoff et al., 2008 |  |  | × |  |  |
| Chien et al., 2006 |  |  | × |  |  |
| Medland et al., 1998 |  |  | × |  |  |
| Laurette et al., 2007 |  |  | × |  |  |
| Moreau et al., 2004 |  |  | × |  |  |
| Sabo et al., 1989 |  |  | × |  |  |
| Steel et al., 2008 |  |  | × |  |  |
| Appleyard et al., 2000 |  |  | × |  |  |
| Chavez et al., 1987 |  |  | × |  |  |
| Johnson et al., 1995 |  |  | × |  |  |
| Ågren et al., 2015 |  |  |  | × |  |
| Arthur et al., 2000 |  |  |  | × |  |
| Batterham et al., 2014 |  |  |  | × |  |
| Brummel et al., 2014 |  |  |  | × |  |
| Chen et al., 2011 |  |  |  | × |  |
| Connolly et al., 2015 |  |  |  | × |  |
| Cox et al., 2018 |  |  |  | × |  |
| Cuthbertson et al., 2009 |  |  |  | × |  |
| Demircelik et al., 2016 |  |  |  | × |  |
| Denehy et al., 2013 |  |  |  | × |  |
| Douglas et al., 2007 |  |  |  | × |  |
| Elliott et al., 2011 |  |  |  | × |  |
| Fleischer et al., 2014 |  |  |  | × |  |
| Garrouste-Orgeas et al., 2012 |  |  |  | × |  |
| Giraud et al., 2016 |  |  |  | × |  |
| Hodgson et al., 2016 |  |  |  | × |  |
| Jackson et al., 2012 |  |  |  | × |  |
| Jensen et al., 2016 |  |  |  | × |  |
| Jónasdóttir et al., 2018 |  |  |  | × |  |
| Jones et al., 2010 |  |  |  | × |  |
| Jones et al., 2003 |  |  |  | × |  |
| Kayambu et al., 2015 |  |  |  | × |  |
| Knowles et al., 2009 |  |  |  | × |  |
| McDowell et al., 2017 |  |  |  | × |  |
| McWilliams et al., 2016 |  |  |  | × |  |
| Morris et al., 2016 |  |  |  | × |  |
| Moss et al., 2016 |  |  |  | × |  |
| Schaller et al., 2016 |  |  |  | × |  |
| Schmidt et al., 2016 |  |  |  | × |  |
| Shelly et al., 2017 |  |  |  | × |  |
| Vitacca et al., 2016 |  |  |  | × |  |
| Battle et al., 2018 |  |  |  | × |  |
| Cox et al., 2018 |  |  |  | × |  |
| Demoule et al., 2017 |  |  |  | × |  |
| Sosnowski et al., 2018 |  |  |  | × |  |
| Wright et al., 2018 |  |  |  | × |  |
| Lautrette et al., 2007 |  |  |  |  | × |
| Schneiderman et al., 2003 |  |  |  |  | × |
| White et al., 2018 |  |  |  |  | × |
| Curtis et al., 2016 |  |  |  |  | × |
| Carson et al., 2016 |  |  |  |  | × |
| Garrouste-Orgeas et al., 2016 |  |  |  |  | × |

- Specific consultations and communication

|  | Michalsen et al 2019 | Goldfarb et al 2017 | Bibas et al 2019 | Carruthers et al 2017 |
| --- | --- | --- | --- | --- |
| Van den Bulcke et al., 2016 | × |  |  |  |
| Karanikola et al., 2014 | × |  |  |  |
| Jensen et al., 2013 | × |  |  |  |
| Baggs et al., 1995 | × |  |  |  |
| Lautrette et al., 2007 |  | × | × |  |
| Schneiderman et al., 2003 |  | × | × |  |
| Curtis et al., 2016 |  | × | × |  |
| Curtis et al., 2011 |  | × | × |  |
| Connors et al., 1995 |  | × | × |  |
| Schneiderman et al., 2000 |  | × | × |  |
| Azoulay et al., 2002 |  | × | × |  |
| Wilson et al., 2015 |  | × | × |  |
| Jones et al., 2010 |  | × |  |  |
| Moreau et al., 2004 |  | × |  |  |
| Steel et al., 2008 |  | × |  |  |
| Dowdy et al., 1998 |  | × |  |  |
| Chen et al., 2014 |  | × |  |  |
| Campbell et al., 2003 |  | × |  |  |
| Campbell et al., 2004 |  | × |  |  |
| Norton et al., 2007 |  | × |  |  |
| Curtis et al., 2008 |  | × |  |  |
| Mosenthal et al., 2008 |  | × |  |  |
| McCormick et al., 2010 |  | × |  |  |
| Lamba et al., 2012 |  | × |  |  |
| Braus et al., 2016 |  | × |  |  |
| Lilly et al., 2000 |  | × |  |  |
| Ahrens et al., 2003 |  | × |  |  |
| Burns et al., 2003 |  | × |  |  |
| Lilly et al., 2003 |  | × |  |  |
| Daly et al., 2010 |  | × |  |  |
| Jacobowski et al., 2010 |  | × |  |  |
| Shelton et al., 2010 |  | × |  |  |
| Quenot et al., 2010 |  | × |  |  |
| McCannon et al., 2012 |  | × |  |  |
| Moore et al., 2012 |  | × |  |  |
| Black et al., 2013 |  | × |  |  |
| Huffines et al., 2013 |  | × |  |  |
| Still et al., 2014 |  | × |  |  |
| Spinello, 2015 |  | × |  |  |
| Pagnamenta et al., 2016 |  | × |  |  |
| Jones et al., 2012 |  | × |  |  |
| Bishop et al., 2013 |  | × |  |  |
| Fumagalli et al., 2006 |  | × |  |  |
| Novaes et al., 2001 |  | × |  |  |
| Schnell et al., 2013 |  | × |  |  |
| Roland et al., 2001 |  | × |  |  |
| Chapman et al., 2016 |  | × |  |  |
| Mitchell et al., 2009 |  | × |  |  |
| Topolovec-Vranic et al., 2010 |  | × |  |  |
| Jongerden et al., 2013 |  | × |  |  |
| White et al., 2018 |  |  | × |  |
| Carson et al., 2016 |  |  | × |  |
| Torke et al., 2016 |  |  | × |  |
| Andereck et al., 2014 |  |  | × |  |
| Cheung et al., 2010 |  |  | × |  |
| El-Soussi et al., 2014 |  |  |  | × |
| Happ et al., 2004 |  |  |  | × |
| Happ et al., 2005 |  |  |  | × |
| Happ et al., 2014 |  |  |  | × |
| Happ et al., 2015 |  |  |  | × |
| Maringelli et al., 2013 |  |  |  | × |
| Miglietta et al., 2004 |  |  |  | × |
| Nilsen et al., 2014 |  |  |  | × |
| Otuzoglu et al., 2014 |  |  |  | × |
| Rodriguez et al., 2010 |  |  |  | × |
| Rodriguez et al., 2012 |  |  |  | × |
| Rodriguez et al., 2016 |  |  |  | × |

- Rehabilitation and mobilization
- Rehabilitation techniques

|  | Waldauf et al 2020 | Nydahl et al 2017 |
| --- | --- | --- |
| Nava, 1998 | × |  |
| Zanotti et al., 2003 | × |  |
| Burtin et al., 2009 | × |  |
| Gerovasili et al., 2009 | × |  |
| Schweickert et al., 2009 | × | × |
| Routsi et al., 2010 | × |  |
| Chen et al., 2011 | × |  |
| Chen et al., 2012 | × |  |
| Dantas et al., 2012 | × |  |
| Abu-Khaber et al., 2013 | × |  |
| Denehy et al., 2013 | × |  |
| Parry et al., 2014 | × |  |
| Dong et al., 2014 | × |  |
| Kho et al., 2014 | × |  |
| Brummel et al., 2014 | × | × |
| Kayambu et al., 2015 | × | × |
| Koçan Kurtoğlu et al., 2015 | × |  |
| Goll et al., 2015 | × |  |
| Fischer et al., 2016 | × |  |
| Hodgson et al., 2016 | × |  |
| Schaller et al., 2016 | × |  |
| Morris et al., 2016 | × |  |
| Coutinho et al., 2016 | × |  |
| Dong et al., 2016 | × |  |
| Frazzitta et al., 2016 | × |  |
| Moss et al., 2016 | × | × |
| Maffei et al., 2017 | × |  |
| Shen et al., 2017 | × |  |
| Machado et al., 2017 | × |  |
| França et al., 2017 | × |  |
| Dall’Acqua et al., 2017 | × |  |
| Eggmann et al., 2018 | × |  |
| Fontes Cerqueira et al., 2018 | × |  |
| Wright et al., 2018 | × |  |
| Fossat et al., 2018 | × |  |
| McWilliams et al., 2018 | × |  |
| Winkelman et al., 2018 | × |  |
| Hickmann et al., 2018 | × |  |
| Valdez et al., 2018 | × |  |
| Koutsioumpa et al., 2018 | × |  |
| Amundadottir et al., 2019 | × |  |
| Kho et al., 2019 | × |  |
| Shaolin et al., 2019 | × |  |
| Stiller et al., 2004 |  | × |
| Zafiropoulos et al., 2004 |  | × |
| Bailey et al., 2007 |  | × |
| Morris et al., 2008 |  | × |
| Skinner et al., 2009 |  | × |
| Bourdin et al., 2010 |  | × |
| Hildreth et al., 2010 |  | × |
| Needham et al., 2010a |  | × |
| Pohlman et al., 2010 |  | × |
| Zanni et al., 2010 |  | × |
| Garzon-Serrano et al., 2011 |  | × |
| Nordon-Craft et al., 2011 |  | × |
| Perme et al., 2011 |  | × |
| Berney et al., 2012 |  | × |
| Genc et al., 2012 |  | × |
| Kho et al., 2012 |  | × |
| Leditschke et al., 2012 |  | × |
| Winkelman et al., 2012 |  | × |
| Berney et al., 2013 |  | × |
| Clark et al., 2013 |  | × |
| Damluji et al., 2013 |  | × |
| Davis et al., 2013 |  | × |
| Hanekom et al., 2013 |  | × |
| Medrinal et al., 2013 |  | × |
| Olkowski et al., 2013 |  | × |
| Perme et al., 2013 |  | × |
| Abrams et al., 2014 |  | × |
| Dinglas et al., 2014 |  | × |
| Nydahl et al., 2014 |  | × |
| Roberts et al., 2014 |  | × |
| Sricharoenchai et al., 2014 |  | × |
| Wang et al., 2014 |  | × |
| Dafoe et al., 2015 |  | × |
| Fields et al., 2015 |  | × |
| Lee et al., 2015 |  | × |
| Lima et al., 2015 |  | × |
| Pires Neto et al., 2015 |  | × |
| Piva et al., 2015 |  | × |
| Skinner et al., 2015 |  | × |
| Hodgson et al., 2015 |  | × |
| Lee et al., 2016 |  | × |
| McGarrigle et al., 2016 |  | × |
| Toonstra et al., 2016 |  | × |
| Umei et al, 2016 |  | × |

- Early mobilization

|  | Zang et al 2020 | Zhang et al 2019 | Doiron et al 2018 | Fuke et al 2018 | Worraphan et al 2020 |
| --- | --- | --- | --- | --- | --- |
| Moss et al., 2016 | × | × |  |  |  |
| Burtin et al., 2009 | × | × |  |  | × |
| Fossat et al., 2018 | × | × |  |  |  |
| Gruther et al., 2017 | × |  |  |  |  |
| Hodgson et al., 2016 | × | × |  | × | × |
| Hu et al., 2014 | × |  |  |  |  |
| Huang et al., 2016 | × |  |  |  |  |
| Kayambu et al., 2019 | × | × |  |  |  |
| Lin et al., 2016 | × |  |  |  |  |
| Morris et al., 2016 | × | × | × | × |  |
| Patel et al., 2014 | × |  |  |  |  |
| Schaller et al., 2016 | × | × |  |  |  |
| Schweickert et al., 2009 | × | × | × | × | × |
| Wang et al., 2018 | × |  |  |  |  |
| Yang et al., 2018 | × |  |  |  |  |
| Kho et al., 2019 |  | × |  |  |  |
| Sarfati et al., 2018 |  | × |  |  |  |
| McWilliams et al., 2018 |  | × |  |  |  |
| Hickmann et al., 2018 |  | × |  |  |  |
| Eggmann et al., 2018 |  | × |  |  |  |
| Maffei et al., 2017 |  | × |  |  | × |
| Machado et al., 2017 |  | × |  |  |  |
| Dong et al., 2016 |  | × |  |  | × |
| Coutinho et al., 2016 |  | × |  |  |  |
| Dong et al., 2014 |  | × |  |  | × |
| Brummel et al., 2014 |  | × |  | × |  |
| Denehy et al., 2013 |  | × |  |  |  |
| Dantas et al., 2012 |  | × |  |  |  |
| Chang et al., 2011 |  | × |  |  | × |
| Nava et al., 1998 |  | × |  |  |  |
| Kayambu et al., 2015 |  |  | × | × |  |
| Patman et al., 2001; |  |  | × |  |  |
| Jones et al., 2015 |  |  |  | × |  |
| Hu et al., 2014 |  |  |  |  |  |
| Ke et al., 2016 |  |  |  |  |  |
| Morris et al., 2008 |  |  |  |  |  |
| Moss et al., 2015 |  |  |  |  |  |
| Schweicket et al., 2009 |  |  |  |  |  |
| Shao et al., 2015 |  |  |  |  |  |
| Yu et al., 2013 |  |  |  |  |  |
| Yu et al., 2016 |  |  |  |  |  |
| Zhang et al., 2017 |  |  |  |  |  |
| Caruso et al., 2005 |  |  |  |  | × |
| Cader et al., 2010 |  |  |  |  | × |
| Martin et al., 2011 |  |  |  |  | × |
| Condessa et al., 2013 |  |  |  |  | × |
| Dixit et al., 2014 |  |  |  |  | × |
| Mohamed et al., 2014 |  |  |  |  | × |
| Shimizu et al., 2014 |  |  |  |  | × |
| Tonella et al., 2017 |  |  |  |  | × |
| Chen et al., 2012 |  |  |  |  | × |
| Franca et al., 2017 |  |  |  |  | × |
| Sandoval et al., 2018 |  |  |  |  | × |

- Inspiratory Muscle Training

|  | Wu et al 2019 | Vorona et al 2018 | Worraphan et al 2020 |
| --- | --- | --- | --- |
| Caruso et al., 2005 | × | × | × |
| Cader et al., 2010 | × | × | × |
| Martin et al., 2011 | × | × | × |
| Condessa et al., 2013 | × | × | × |
| Dixit et al., 2014 | × | × | × |
| Elbouhy et al., 2014 | × | × |  |
| Ibrahiem et al., 2014 | × | × |  |
| Mohamed et al., 2014 | × | × | × |
| Pascotini et al., 2014 | × | × |  |
| Shimizu et al., 2014 | × | × | × |
| Bissett et al., 2016 | × | × |  |
| Huang et al., 2017 | × |  |  |
| Tonella et al., 2017 | × | × | × |
| Sandoval et al., 2018 | × |  |  |
| Chang et al., 2011 |  | × | × |
| Holliday et al., 1990 |  | × |  |
| Melo et al., 2017 |  | × |  |
| Nava et al., 1998 |  | × |  |
| Özyürek et al., 2014 |  | × |  |
| Porta et al., 2005 |  | × |  |
| Saad et al., 2014 |  | × |  |
| Shrestha et al., 2014 |  | × |  |
| Yosef-Brauner et al. 2015 |  | × |  |
| Aldrich et al., 1989 |  | × |  |
| Barros et al., 2015 |  | × |  |
| Chiang et al., 2006 |  | × |  |
| Martin AD et al., 2002 |  | × |  |
| Martin UJ et al., 2005 |  | × |  |
| Sprague et al., 2003 |  | × |  |
| Supinski et al., 2017 |  | × |  |
| Burtin et al., 2009 |  |  | × |
| Hodgson et al., 2016 |  |  | × |
| Schweickert et al., 2009 |  |  | × |
| Maffei et al., 2017 |  |  | × |
| Dong et al., 2016 |  |  | × |
| Dong et al., 2014 |  |  | × |
| Chen et al., 2012 |  |  | × |
| Franca et al., 2017 |  |  | × |
| Sandoval et al., 2018 |  |  | × |

- Interventions related to reducing the duration of mechanical ventilation
- Weaning modes or protocols

|  | Ou-Yang et al 2020 | Kataoka et al 2018 | Neuschwander et al 2021 | Hirzallah et al 2019 |
| --- | --- | --- | --- | --- |
| Xirouchaki et al., 2008 | × | × |  |  |
| Sasikumar et al., 2013 | × |  |  |  |
| Elganady et al., 2014 | × |  |  |  |
| Teixeira et al., 2015 | × |  |  |  |
| Bosma et al., 2016 | × | × | × |  |
| Botha et al., 2018 | × | × |  |  |
| Salama et al., 2018 | × |  |  |  |
| Colombo et al., 2008 |  | × |  |  |
| Spahija et al., 2010 |  | × |  |  |
| Patroniti et al., 2012 |  | × |  |  |
| Elganadyet al., 2014 |  | × |  |  |
| Doorduin et al., 2015 |  | × |  |  |
| Schmidt et al., 2015 |  | × |  |  |
| Carteaux et al., 2016 |  | × |  |  |
| Demoule et al., 2016 |  | × |  |  |
| Di Mussi et al., 2016 |  | × |  |  |
| Kuo et al., 2016 |  | × |  |  |
| Costa et al., 2017 |  | × |  |  |
| Ferreira et al., 2017 |  | × |  |  |
| Agarwal et al., 2013 |  |  | × |  |
| Aghadavoudi et al., 2012 |  |  | × |  |
| Arnal et al., 2018 |  |  | × |  |
| Burns et al., 2013 |  |  | × |  |
| Celli et al., 2014 |  |  | × |  |
| Dongelmans et al., 2009 |  |  | × |  |
| Fot et al., 2017 |  |  | × |  |
| Gruber et al., 2008 |  |  | × |  |
| Hendrix et al., 2006 |  |  | × |  |
| Kirakli et al., 2015 |  |  | × |  |
| Kirakli et al., 2010 |  |  | × |  |
| Lellouche et al., 2006 |  |  | × |  |
| Lellouche et al., 2013 |  |  | × |  |
| Mohamed et al., 2014 |  |  | × |  |
| Moradian et al., 2017 |  |  | × |  |
| Petter et al., 2003 |  |  | × |  |
| Rose et al., 2008 |  |  | × |  |
| Roth et al., 2001 |  |  | × |  |
| Schadler et al., 2012 |  |  | × |  |
| Stahl et al., 2009 |  |  | × |  |
| Taniguchi et al., 2009 |  |  | × |  |
| Taniguchi et al., 2015 |  |  | × |  |
| Zhu et al., 2015 |  |  | × |  |
| Danckers et al., 2013 |  |  |  | × |
| Roh et al., 2012 |  |  |  | × |
| Tonnelier et al., 2005 |  |  |  | × |

- Ventilator bundle or cough augmentation techniques

|  | Pileggi et al 2018 | Rose et al 2017 |
| --- | --- | --- |
| Bloos et al., 2009 | × |  |
| Arroliga et al., 2012 | × |  |
| Cachecho et al., 2012 | × |  |
| Ding et al., 2013 | × |  |
| Hawe et al., 2009 | × |  |
| Stone et al., 2011 | × |  |
| Parisi et al., 2016 | × |  |
| Crunden et al., 2005 | × |  |
| Marra et al., 2009 | × |  |
| Dubose et al., 2010 | × |  |
| Morris et al., 2011 | × |  |
| Pérez-Granda et al., 2014 | × |  |
| DeLuca et al., 2017 | × |  |
| Crowe et al., 2006 |  | × |
| Gonçalves et al., 2012 |  | × |
| Niranjan et al., 1998 |  | × |

- Early tracheostomy

|  | Wang et al 2019 | McCredie et al 2017 | de Franca et al 2020 |
| --- | --- | --- | --- |
| Dunham et al., 1984 | × |  |  |
| Rodriguez et al., 1990 | × |  |  |
| Sugerman et al., 1997 | × | × |  |
| Saffle et al., 2002 | × |  |  |
| Bouderka et al., 2004 | × |  |  |
| Rumbak et al., 2004 | × |  |  |
| Barquist et al., 2006 | × | × |  |
| Blot et al., 2008 | × | × |  |
| Saboori et al., 2009 | × |  |  |
| Terragni et al., 2010 | × | × |  |
| Trouillet et al., 2011 | × |  |  |
| Bylappa et al., 2011 | × |  |  |
| Zheng et al., 2012 | × |  |  |
| Koch et al., 2012 | × |  |  |
| Young et al., 2013 | × | × |  |
| Bösel et al., 2013 | × | × |  |
| Diaz-Prieto et al., 2014 | × |  |  |
| Mohamed et al., 2014 | × | × |  |
| Dunham et al., 2014 |  | × | × |
| Fayed et al., 2012 |  | × |  |
| Alali et al., 2014 |  |  | × |
| Ahmed et al., 2007 |  |  | × |
| Huang et al., 2013 |  |  | × |
| Khalili et al., 2017 |  |  | × |
| Rizk et al., 2011 |  |  | × |
| Shibahashi et al., 2017 |  |  | × |
| Siddiqui et al., 2015 |  |  | × |

- High-flow nasal cannula

|  | Granton et al 2020 | Chaudhuri et al 2020 | Zhu et al 2019 | Huang et al 2018 | Lewis et al 2021 | Zhao et al 2017 |
| --- | --- | --- | --- | --- | --- | --- |
| Theerawit et al., 2017 | × |  |  |  |  |  |
| Rittayamai et al., 2014 | × |  | × |  | × |  |
| Jing et al., 2019 | × |  |  |  | × |  |
| Song et al., 2017 | × |  | × |  | × |  |
| Maggiore et al., 2014 | × |  | × | × |  | × |
| Hernández et al., 2016a | × |  | × | × | × | × |
| Hernández et al., 2016b | × |  |  | × | × | × |
| Fernandez et al., 2017 | × |  | × |  | × |  |
| Simon et al., 2016 |  | × |  |  |  |  |
| Frat et al., 2019 |  | × |  |  |  |  |
| Vourc’h et al., 2015 |  | × |  |  |  |  |
| Mir et al., 2017 |  | × |  |  |  |  |
| Guitton et al., 2019 |  | × |  |  |  |  |
| Sebastian et al., 2014 |  | × |  |  |  |  |
| Jaber et al., 2016 |  | × |  |  |  |  |
| Ng et al., 2018 |  | × |  |  |  |  |
| Lodenius et al., 2018 |  | × |  |  |  |  |
| Vourc’h et al., 2019 |  | × |  |  |  |  |
| Parke et al., 2013 |  |  | × | × | × | × |
| Corley et al., 2015 |  |  | × | × |  | × |
| Tiruvoipati et al., 2010 |  |  | × |  |  |  |
| Di et al., 2018 |  |  | × |  |  |  |
| Futier et al., 2016 |  |  | × | × | × |  |
| Stephan et al., 2015 |  |  |  | × | × | × |
| Azoulay et al., 2018 |  |  |  |  | × |  |
| Brainard et al., 2017 |  |  |  |  | × |  |
| Chanques et al., 2013 |  |  |  |  | × |  |
| Cong et al., 2019 |  |  |  |  | × |  |
| Corley et al., 2014 |  |  |  |  | × |  |
| Cuquemelle et al., 2012 |  |  |  |  | × |  |
| Frat et al., 2015 |  |  |  |  | × | × |
| Grieco et al., 2020 |  |  |  |  | × |  |
| Hu et al., 2020 |  |  |  |  | × |  |
| Lee et al., 2018 |  |  |  |  | × |  |
| Lemiale et al., 2015 |  |  |  |  | × | × |
| Longhini et al., 2019 |  |  |  |  | × |  |
| Mauri et al., 2017a |  |  |  |  | × |  |
| Mauri et al., 2017b |  |  |  |  | × |  |
| Parke et al., 2011 |  |  |  |  | × | × |
| Schwabbauer et al., 2014 |  |  |  |  | × |  |
| Shebl et al., 2018 |  |  |  |  | × |  |
| Vargas et al., 2015 |  |  |  |  | × |  |
| Vourc'h et al., 2020 |  |  |  |  | × |  |
| Yu et al., 2017 |  |  |  |  | × |  |
| Zochios et al., 2018 |  |  |  |  | × |  |
| Bell et al., 2015 |  |  |  |  |  | × |
| Jones et al., 2015 |  |  |  |  |  | × |

- Management of specific symptoms (delirium, agitation, pain, and sleep disturbances)
- Delirium

|  | Liang et al 2021 | Bannon et al 2019 | León-Salas et al 2020 | Herling et al 2018 | Lu et al 2019 | Litton et al 2016 | Geense et al 2019 |
| --- | --- | --- | --- | --- | --- | --- | --- |
| Balas et al., 2014 | × |  |  |  |  |  |  |
| Jacob et al., 2017 | × |  |  |  |  |  |  |
| Black et al., 2011 | × |  |  |  |  |  |  |
| Lee et al., 2013 | × |  |  |  |  |  |  |
| Colombo et al., 2012 | × |  |  |  |  |  |  |
| Rosa et al., 2017 | × |  |  |  |  |  |  |
| Huang et al., 2014 | × |  |  |  |  |  |  |
| Chen et al., 2018 | × |  |  |  |  |  |  |
| Zhang et al., 2015 | × |  |  |  |  |  |  |
| Zhao et al., 2018 | × |  |  |  |  |  |  |
| Lee et al., 2014 | × |  |  |  |  |  |  |
| Kram et al., 2015 | × |  |  |  |  |  |  |
| Ma et al., 2015 | × |  |  |  |  |  |  |
| Simons et al., 2016 | × | × |  | × |  |  |  |
| Chevillon et al., 2015 | × |  |  |  |  |  |  |
| Smith et al., 2016 | × |  |  |  |  |  |  |
| Guo et al., 2016a | × |  |  |  |  |  |  |
| Bryczkowski et al., 2014 | × |  |  |  |  |  |  |
| Karadas et al., 2016 | × | × |  |  |  |  |  |
| Fraser et al., 2015 | × |  |  |  |  |  |  |
| Schweickert et al., 2009 | × | × |  |  |  |  |  |
| Guo et al., 2016b | × |  | × |  |  |  |  |
| Zaal et al., 2013 | × |  |  |  |  |  |  |
| Wang et al., 2016 | × |  |  |  |  |  |  |
| Bounds et al., 2016 | × |  |  |  |  |  |  |
| Rivosecchi et al., 2016 | × |  |  |  |  |  |  |
| Martinez et al., 2017 | × |  |  |  |  |  |  |
| Moon et al., 2015 | × | × |  | × |  |  |  |
| Zhang et al., 2017 | × |  |  |  |  |  |  |
| Huang et al., 2017 | × |  |  |  |  |  |  |
| Dou et al., 2018 | × |  |  |  |  |  |  |
| Gan et al., 2017 | × |  |  |  |  |  |  |
| Patel et al., 2014 | × |  |  |  |  | × |  |
| Van Rompaey et al., 2012 | × | × |  | × |  | × |  |
| Ono et al., 2011 |  | × |  |  | × |  |  |
| Taguchi et al., 2007 |  | × |  |  | × |  |  |
| Potharajaroen et al., 2018 |  | × |  |  | × |  |  |
| Álvarez et al., 2017 |  | × |  |  |  |  |  |
| Morris et al., 2016 |  | × |  |  |  |  | × |
| Finotto et al., 2006 |  | × |  |  |  |  |  |
| Girard et al., 2008 |  | × |  |  |  |  |  |
| Mehta et al., 2012 |  | × |  | × |  |  |  |
| Munro et al., 2017 |  | × |  |  |  |  |  |
| Giraud et al., 2016 |  | × |  |  |  |  | × |
| Karadas et al., 2016 |  | × |  |  |  |  |  |
| Abizanda et al., 2011 |  |  | × |  |  |  |  |
| Avendaño-Céspedes et al., 2016 |  |  | × |  |  |  |  |
| Chen et al., 2017 |  |  | × |  |  |  |  |
| Freter et al., 2017 |  |  | × |  |  |  |  |
| Hempenius et al., 2013a |  |  | × |  |  |  |  |
| Hempenius et al., 2013b |  |  | × |  |  |  |  |
| Jeffs et al., 2013 |  |  | × |  |  |  |  |
| Lundström et al., 2007 |  |  | × |  |  |  |  |
| Marcantonio et al., 2001 |  |  | × |  |  |  |  |
| Martínez et al., 2012 |  |  | × |  |  |  |  |
| Abdelgalel et al., 2016 |  |  |  | × |  |  |  |
| Brummel et al., 2014 |  |  |  | × |  |  |  |
| Nassar et al., 2014 |  |  |  | × |  |  |  |
| Page et al., 2013 |  |  |  | × |  |  |  |
| Pandharipande et al., 2007 |  |  |  | × |  |  |  |
| Shehabi et al., 2013 |  |  |  | × |  |  |  |
| Strøm et al., 2010 |  |  |  | × |  |  |  |
| Van Den Boogard et al., 2018 |  |  |  | × |  |  |  |
| Le Guen et al., 2014 |  |  |  |  | × |  |  |
| Yang et al., 2015 |  |  |  |  | × |  |  |
| Su et al., 2016 |  |  |  |  | × |  |  |
| Wu et al., 2016 |  |  |  |  | × |  |  |
| Li et al., 2017 |  |  |  |  | × |  |  |
| Lu et al., 2017 |  |  |  |  | × |  |  |
| Wang et al., 2018 |  |  |  |  | × |  |  |
| Sultan et al., 2010 |  |  |  |  | × |  |  |
| de Jonghe et al., 2014 |  |  |  |  | × |  |  |
| Abbasi et al., 2018 |  |  |  |  | × |  |  |
| Chlan et al., 2013 |  |  |  |  |  | × |  |
| Foreman et al., 2015 |  |  |  |  |  | × |  |
| Hu et al., 2015 |  |  |  |  |  | × |  |
| Jones et al., 2012 |  |  |  |  |  | × |  |
| Kamdar et al., 2013 |  |  |  |  |  | × |  |
| Le Guen et al., 2013 |  |  |  |  |  | × |  |
| Scotto et al., 2009 |  |  |  |  |  | × |  |
| Ågren et al., 2015 |  |  |  |  |  |  | × |
| Arthur et al., 2000 |  |  |  |  |  |  | × |
| Batterham et al., 2014 |  |  |  |  |  |  | × |
| Brummel et al., 2014 |  |  |  |  |  |  | × |
| Chen et al., 2011 |  |  |  |  |  |  | × |
| Connolly et al., 2015 |  |  |  |  |  |  | × |
| Cox et al., 2018a |  |  |  |  |  |  | × |
| Cuthbertson et al., 2009 |  |  |  |  |  |  | × |
| Demircelik et al., 2016 |  |  |  |  |  |  | × |
| Denehy et al., 2013 |  |  |  |  |  |  | × |
| Douglas et al., 2007 |  |  |  |  |  |  | × |
| Elliott et al., 2011 |  |  |  |  |  |  | × |
| Fleischer et al., 2014 |  |  |  |  |  |  | × |
| Garrouste-Orgeas et al., 2012 |  |  |  |  |  |  | × |
| Hodgson et al., 2016 |  |  |  |  |  |  | × |
| Jackson et al., 2012 |  |  |  |  |  |  | × |
| Jensen et al., 2016 |  |  |  |  |  |  | × |
| Jónasdóttir et al., 2018 |  |  |  |  |  |  | × |
| Jones et al., 2010 |  |  |  |  |  |  | × |
| Jones et al., 2003 |  |  |  |  |  |  | × |
| Kayambu et al., 2015 |  |  |  |  |  |  | × |
| Knowles et al., 2009 |  |  |  |  |  |  | × |
| McDowell et al., 2017 |  |  |  |  |  |  | × |
| McWilliams et al., 2016 |  |  |  |  |  |  | × |
| Moss et al., 2016 |  |  |  |  |  |  | × |
| Schaller et al., 2016 |  |  |  |  |  |  | × |
| Schmidt et al., 2016 |  |  |  |  |  |  | × |
| Shelly et al., 2017 |  |  |  |  |  |  | × |
| Vitacca et al., 2016 |  |  |  |  |  |  | × |
| Battle et al., 2018 |  |  |  |  |  |  | × |
| Cox et al., 2018b |  |  |  |  |  |  | × |
| Demoule et al., 2017 |  |  |  |  |  |  | × |
| Sosnowski et al., 2018 |  |  |  |  |  |  | × |
| Wright et al., 2018 |  |  |  |  |  |  | × |

- Agitation

|  | Aitken et al 2018 | Nassar et al 2016 | Stephens et al 2018 | Long et al 2020 | Shetty et al 2018 |
| --- | --- | --- | --- | --- | --- |
| Brook et al., 1999 | × |  |  |  |  |
| Bucknall et al., 2008 | × |  |  |  |  |
| Curley et al., 2015 | × |  |  |  |  |
| Mansouri et al., 2013 | × |  |  |  |  |
| Mehta et al., 2008 |  | × |  |  |  |
| de Wit et al., 2008 |  | × |  |  |  |
| Anifantaki et al., 2009 |  | × |  |  |  |
| Strom et al., 2010 |  | × |  |  |  |
| Yiliaz et al., 2010 |  | × |  |  |  |
| Mehta et al., 2013 |  | × |  |  |  |
| Nassar et al., 2014 |  | × |  | × |  |
| Shehabi et al., 2013a |  |  | × | × |  |
| Shehabi et al., 2013b |  |  | × | × |  |
| Shehabi et al., 2012 |  |  | × | × |  |
| van den Boogaard et al., 2012 |  |  | × | × |  |
| Shehabi et al., 2013c |  |  | × |  |  |
| Samarin et al., 2014 |  |  | × |  |  |
| Tanaka et al., 2014 |  |  | × |  |  |
| Balzer et al., 2015 |  |  | × | × |  |
| Stephens et al., 2017 |  |  | × | × |  |
| Pandharipande et al., 2007 |  |  |  | × |  |
| Samuelson et al., 2008 |  |  |  | × |  |
| Girard et al., 2008 |  |  |  | × |  |
| Treggiari et al., 2009 |  |  |  | × |  |
| Strøm et al., 2010 |  |  |  | × |  |
| Hager et al., 2013 |  |  |  | × |  |
| Dale et al., 2014 |  |  |  | × |  |
| Skrupky et al., 2015 |  |  |  | × |  |
| Kawazoe et al., 2017 |  |  |  | × |  |
| Kaplan et al., 2018 |  |  |  | × |  |
| De Jonghe et al., 2018 |  |  |  | × |  |
| Inaba et al., 2007 |  |  |  |  | × |
| Li et al., 2009 |  |  |  |  | × |
| Weatherburn et al., 2007 |  |  |  |  | × |
| Zhao et al., 2011 |  |  |  |  | × |

- Pain (Not calculated)
- Sleep disturbances (Not calculated)

**Table 5 General characteristics of systematic reviews included in the umbrella review**

| Author/year | Objective | Outcomes | Effect size  (95% CI) | N/n | Quality  (GDARE) | Conclusions |
| --- | --- | --- | --- | --- | --- | --- |
| Multicomponent interventions involving education of healthcare professionals | | | | | | |
| Franks et al  2021 | To investigate the association between PR use and PTSD symptoms in ICU survivors | PTSD | OR 6.04  (2.21, 16.33) | 98  (1 observational study) | VERY LOW | PR use may be associated with PTSD in ICU survivors and is associated with delirium and longer duration of mechanical ventilation. Nurse education is likely effective in reducing rates of PR among ICU patients. |
|  | To examine the cognitive and physical outcomes associated with PR use | Delirium | OR 11.54  (6.66, 20.01) | 2895  (15 observational studies) | VERY LOW |  |
|  |  | Duration of MV | MD 3.35  (1.95, 4.75) | 1011  (2 observational studies) | VERY LOW |  |
|  | To assess interventions that may be effective in reducing restraint use | PR use reduction | OR 0.48  (0.32, 0.73) | 44229  (6 observational studies) | VERY LOW |  |
| Family engagement/support | | | | | | |
| Nassar et al  2018 | To synthesize data on outcomes related to patients, family members, and ICU professionals by comparing flexible versus restrictive visiting policies in ICUs. | Delirium | OR 0.39  (0.22, 0.69) | 354  (1 RCT, 1 observational study) | LOW | Flexible ICU visiting hours have the potential to reduce delirium and anxiety symptoms among patients and to improve family members’ satisfaction. However, they may be associated with an increased risk of burnout among ICU professionals. These conclusions are based on few studies, with small samples and moderate risk of bias. |
|  |  | Depressive symptoms | MD -1.91  (-4.35, 0.52) | 285  (1 RCT, 1 observational study) | VERY LOW |  |
|  |  | Anxiety symptoms | MD -2.20  (-3.80, -0.61) | 285  (1 RCT, 1 observational study) | VERY LOW |  |
|  |  | ICU mortality | OR 0.71  (0.38, 1.36) | 60509  (3 observational studies) | VERY LOW |  |
|  |  | ICU-acquired infection | OR 0.98  (0.68, 1.42) | 1042  (1 RCT, 2 observational studies) | LOW |  |
|  |  | ICU LOS | MD -0.26  (-0.57, 0.05) | 26576  (3 observational studies) | VERY LOW |  |
| Galvin et al  2018 | To systematically review and evaluate the effects of humanized care (①liberal visitation, ②witnessed resuscitation, ③diaries, ④family involvement in basic patient care) of the critically ill on empathy among healthcare professionals, anxiety among relatives, and burnout and compassion fatigue in both groups | ①Burnout  (ICU professionals) | No consistent trend identified | | VERY LOW* | There is insufficient evidence to make any quantitative assessment of the effect of humanizing interventions on any of these psychologic outcomes. There is a trend towards reduced anxiety among family members who participated in basic patient care, liberal visitation, and diary keeping. There are conflicting effects of liberal visitation on burnout among healthcare professionals. |
|  |  | ①Anxiety  (Family members) | Trend towards reduction of anxiety among relatives but not consistently significant | | LOW* |  |
|  |  | ②Anxiety  (Family members) | Consistent trend showing no significant difference in anxiety levels | | LOW* |  |
|  |  | ③Anxiety  (Family members) | No significant trend towards reduction in anxiety among relatives | | LOW* |  |
|  |  | ④Burnout  (ICU professionals) | Only one potentially eligible study was identified, this showed a significant decrease in burnout. However, it involved a multifaceted intervention with several other components | | VERY LOW* |  |
|  |  | ④Anxiety  (Family members) | Consistent trend towards reduction in anxiety among relatives | | LOW* |  |
| Lee et al  2019 | To elucidate the impact of protocolized family support intervention (Medical information and emotional support) on ICU LOS | ICU LOS | MD -0.89  (-1.50, -0.27) | 3477  (7 RCTs) | HIGH | Protocolized family support intervention for enhanced communication and shared decision-making with the family reduced ICU LOS in critically ill patients without impacting mortality. |
|  |  | Hospital LOS | MD -3.78  (-5.26, -2.29) | 1562  (4 RCTs) | HIGH |  |
|  |  | Mortality in ICU | OR 0.76  (0.45, 1.29) | 267  (2 RCTs) | LOW |  |
|  |  | Mortality in hospital | OR 1.13  (0.85, 1.49) | 2375  (4 RCTs) | LOW |  |
|  | Therapeutic goal of comfort care setting | ICU LOS | MD -1.26  (-2.21, -0.31) | 1534  (3 RCTs) | HIGH |  |
|  | Therapeutic goal of curative care setting | ICU LOS | MD -0.61  (-1.42, 0.19) | 1943  (4 RCTs) | MODERATE |  |
|  | Measurement from post-intervention | ICU LOS | MD -0.89  (-1.55, -0.22) | 2881  (4 RCTs) | HIGH |  |
|  | Measurement from baseline | ICU LOS | MD -0.77  (-2.96, 1.43) | 596  (3 RCTs) | MODERATE |  |
|  | Timing of intervention (≤72 h) | ICU LOS | MD -1.07  (-2.12, -0.02) | 1633  (4 RCTs) | HIGH |  |
|  | Timing of intervention (>72 h) | ICU LOS | MD -0.69  (-1.53, 0.14) | 1676  (2 RCTs) | MODERATE |  |
| Geense et al  2019 | To assess the effectiveness of nonpharmacologic interventions (①EGDM, ②diaries, ③information and education, ④earplugs and eye mask) on adverse long-term outcomes among ICU survivors. | ④Sleep quality | Mdn D 3  [Range 5 to 11] | 64  (1 RCT) | LOW | There is thin evidence that diaries and exercise programs have a positive effective on mental outcomes. Despite outcomes favoring the intervention group, other commonly used nonpharmacologic interventions in daily ICU practice are not supported by conclusive evidence from this meta-analysis. |
|  |  | ①Depression | MD 0.30  (-4.19, 4.79) | 50  (1RCT) | LOW |  |
|  |  | ②Depression | SMD 0.68  (0.14, 1.21) | 88  (2 RCTs) | VERY LOW |  |
|  |  | ③Depression | MD 0.30  (0.08, 0.52) | 100  (1 RCT) | LOW |  |
|  |  | ④Depression | Mdn D -3.5  [Range 2 to 9] | 64  (1 RCT) | LOW |  |
|  |  | ①Anxiety | MD 0.30  (-4.19, 4.79) | 50  (1RCT) | LOW |  |
|  |  | ②Anxiety | SMD 0.44  (0.01, 0.87) | 88  (2 RCTs) | VERY LOW |  |
|  |  | ③Anxiety | MD 0.40  (0.14, 0.66) | 100  (1 RCT) | LOW |  |
|  |  | ④Anxiety | Mdn D -1  [Range 6 to 11] | 64  (1 RCT) | LOW |  |
|  |  | ②PTSD | Mdn D -2.5  [Range 13 to 84] | 352  (1 RCT) | MODERATE |  |
|  |  | ④PTSD | Mdn D -5  [Range 5 to 27] | 64  (1 RCT) | LOW |  |
|  |  | ①Global recognition | MD 0.3  (-0.7, 1.3) | 300  (1 RCT) | MODERATE |  |
| Kynoch et al  2016 | To establish recent best practice (①support group, ②family support coordinators, ③family maintained progress journal or diary, ④ communication interventions, ⑤open or more flexible visiting hours, ⑥information provided to families, ⑦ environmental changes)in addressing the needs of family members with a relative or loved one admitted to an adult critical care unit. | ①Stress | In Sabo et al (1989), there was no statistically significant difference between the two groups for mean stress scores. | | VERY LOW | Future intervention studies focusing on family needs could include the use of technology such as DVDs (Digital Versatile Discs) and SMS (Short Message Service) for informing families and interventions specifically designed to improve family comfort. Communication interventions help promote family involvement in their loved one’s care and facilitate their decision-making capacity, as well as improving clinician and family interaction, family comprehension of their loved one’s condition and also reduce the development of post-traumatic stress-related symptoms. Family satisfaction may be increased with the provision of comfortable physical environments with noise reduction measures. |
|  |  | ①Satisfaction  (Family members) | In Steel et al (2008), mean satisfaction for the control and the intervention group was 4.50 (SD=0.20) and 4.55 (SD=0.17), respectively. The difference between the two groups was not statistically significant (P=0.35). | | VERY LOW |  |
|  |  | ②Comfort  (Family members) | In Appleyard et al (2000), the results indicate a statistically significant difference in the CCFNI need for comfort (P<0.05) reported by families before and after initiation of the program. | | VERY LOW |  |
|  |  | ②Satisfaction  (Family members) | In Yousefi et al (2012), the mean satisfaction score for the intervention group was significantly higher (P<0.001) after the intervention than before. In Shelton et al (2010), there was an improvement in family satisfaction with ICU team members care and communication, mostly from physician communication (P=0.003), respiratory therapists care (P=0.004), social workers’ communication (P=0.006) and nursing care although this was not statistically significant (P=0.05). In addition, satisfaction with the ICU team’s consideration of family needs (P=0.001) increased significantly. In Moore et al (2012), FSC significantly increased family satisfaction with physician care and communication and the ICU teams’ consideration of family needs in the ICU environment. | | VERY LOW |  |
|  |  | ③Anxiety  (Family members) | In Kloos et al (2008), the mean anxiety levels were reduced from pre-test to post-test; however, this was not related to the use of the family-maintained progress journal. | | VERY LOW |  |
|  |  | ③PTSD  (Family members) | In Jones et al (2012), a diary for the patient and families may facilitate recollection of the ICU experience and initiate discussions between family members and the patient about their feelings and emotions, potentially reducing the symptoms of PTSS. In Garrouste-Orgeas et al (2012), after 12 months, there was a significant difference in the IES-R scores of the intervention and control groups that the use of a diary can influence the long-term psychological impact of an ICU admission for relatives. | | VERY LOW |  |
|  |  | ④Satisfaction  (Family members) | In Medland et al (1998), the study did show a significant reduction of phone calls from family members in the intervention group versus the control group without compromising family members’ satisfaction of care or their need for information. In Moreau et al (2004), there were no significant differences found between the junior and senior physician groups with respect to satisfaction, anxiety and depression. In Jacobowski et al (2010), while satisfaction did not improve with interactive family rounds, family participation in rounds potentially could improve some aspects of family satisfaction such as support during decision making and frequency of communication with clinicians. | | LOW |  |
|  |  | ⑤Satisfaction  (Family members) | In Ramsey et al (1999), the study found that visitors and nurses were generally happy with a change in visitation policy, from 15 minutes at specified hours five times a day to 15-minute visits at specified hours seven times a day, with families satisfied with the flexibility and close proximity to the patient. In Baharoon et al (2014), the authors did not observe a significant difference between the two visitation policies and adequate allocation of time for discussions, direct communication with clinicians and family socio-cultural characteristics may impact on the overall satisfaction of families. | | VERY LOW |  |
|  |  | ⑥Satisfaction  (Family members) | In Azoulay et al (2002), satisfaction did not significantly differ between the FIL and the control group. However, among family representatives with good comprehension, the FIL was associated with significantly better satisfaction. In Chien et al (2006), the needs-based education program group reported significantly higher levels of satisfaction of family needs. In Henneman et al (1992), comparison of restricted visiting hours group and open visiting hours/information booklet group showed statistically significant increases (P<0.05) in family satisfaction as well as family knowledge of ICU (P<0.05). In Kirchhoff et al (2008), compared with the usual care group, the intervention group was significantly more satisfied with the information they received (P=0.05) and understood better what was to happen (P=0.03). In Kodali et al (2008), there was no significant difference with family satisfaction after the introduction of the communication pathway. In Wysham et al (2014), there was little change to family satisfaction after the implementation of communication tool. | | VERY LOW |  |
|  |  | ⑥Stress  (Family members) | In Chavez et al (1987), there were no statistically significant changes found between relatives in both the education program and the control groups. | | VERY LOW |  |
|  |  | ⑥Anxiety  (Family members) | In Chien et al (2006), the needs-based education program group reported significantly lower levels of state anxiety than the control group at the post-test. In Johnson et al (1995), the mean difference in the decrease of anxiety scores for the telephone intervention group was statistically significant (t = 4.98, P<0.05). | | VERY LOW |  |
|  |  | ⑥Knowledge  (Family members) | In McCannon et al (2012), the decisional video group had statistically significant better knowledge than the control group. | | VERY LOW |  |
|  |  | ⑦Satisfaction  (Family members) | In Jongerden et al (2013), satisfaction with the subscale of ‘‘care’’ increased (P=0.007) although satisfaction with ‘‘decision making’’ was not significantly changed (P=0.12). | | VERY LOW |  |
| Specific consultations and communication | | | | | | |
| Bibas et al  2019 | To perform a SR and MA of RCTs to determine the association of targeted interventions (health care professional-led interventions, ethics consultation and media tools) of improving surrogate decision-making with patient- and family-centered outcomes and resource use. | All-Cause Mortality | RR 1.03  (0.98, 1.08) | 2746  (10 RCTs) | LOW | Systematic interventions aimed at improving surrogate decision-making for critically ill adults may reduce ICU LOS among patients who die in the ICU, without influencing overall mortality. |
|  |  | ICU LOS  (All patients) | MD -0.79  (-2.33, 0.76) | 883  (6 RCTs) | LOW |  |
|  |  | ICU LOS  (Patients who died) | MD -2.11  (-4.16, -0.07) | 829  (9 RCTs) | MODERATE |  |
|  |  | Depression  (Family members) | RoM -0.11  (-0.29, 0.08) | 1365  (5 RCTs) | LOW |  |
|  |  | Anxiety  (Family members) | RoM -0.08  (-0.25, 0.08) | 1367  (5 RCTs) | LOW |  |
|  |  | PTSD  (Family members) | RoM -0.04  (-0.21, 0.13) | 1337  (4 RCTs) | LOW |  |
| Goldfarb et al  2017 | To determine whether PFCC interventions (①ethics consultation, ② communication facilitation)in the ICU setting improved mortality, ICU LOS, or had other beneficial effects. | ①②Mortality | OR 1.07  (0.95, 1.21) | 5650  (5 RCTs) | MODERATE | PFCC-focused interventions are associated with patient and family satisfaction, improved mental health status, and decreased resource utilization in ICUs including decreased LOS but not with changes in mortality rates. |
|  |  | ①ICU LOS | MD -1.21  (-2.25, -0.16) | 3164  (3 RCTs) | HIGH |  |
| Carruthers et al  2017 | To assess the effectiveness of AAC strategies to enable people who are temporarily voiceless due to medical intervention, to communicate | PRs use | In Happ et al (2015), there were no statistically significant differences between intervention and control quarters in proportion of ICU days with PRs (*p* = 0.44). | | MODERATE | There is preliminary, but inconsistent evidence that AAC strategies are effective in improving patient satisfaction with communication and reducing difficulties in communication. A lack of comparable studies precluded the identification of the most effective AAC strategy. |
|  |  | Pain levels | In Happ et al (2015), there were no statistically significant differences between intervention and control quarters in proportion of ICU days with pain score documentation (*p* = 0.97). | | MODERATE |  |
|  |  | Heavy sedation | In Happ et al (2015), there were no statistically significant differences between intervention and control quarters in proportion of ICU days with heavy sedation (*p* = 0.73) | | MODERATE |  |
|  |  | ICU LOS | In Happ et al (2015), there were no statistically significant differences between intervention and control quarters in ICU LOS (*p* = 0.77) | | MODERATE |  |
|  |  | Hospital LOS | In Happ et al (2015), there were no statistically significant differences between intervention and control quarters in hospital LOS (*p* = 0.20) | | MODERATE |  |
|  |  | Patient satisfaction  (High-technology) | The Satisfaction with Communication Method was used in Rodriguez et al. (2016) and statistically significant improvements were found in the intervention group (0.59, p ≤ 0.001) compared to control. | | VERY LOW |  |
|  |  | Patient satisfaction  (Low-technology) | In El-Soussi et al (2014), 40% in the study group were very satisfied compared to 6.66% in the control which was a statistically significant  increase p< 0.001. | | LOW |  |
|  |  | Ease or difficulties in communication  (High-technology) | In Happ et al (2004), Ease of Communication Scale (ECS) measurements showed significantly less difficulty with communication after device use (t > 2.62; P = .047); In Happ et al (2005), postintervention ECS scores (19.8 ± 9.7) among the study group were only slightly lower than ECS scores among the nonintervention, historic control group (22.5 ± 11.3). In Rodriguez et al (2010), Lower proportions of patients reported communication difficulties when using a high technology AAC strategy with the risk ratio of 0.22 indicating a very large effect Happ et al. (2014) reported decreased reports of high difficulty in communication in the third phase when the intervention included high technology AAC strategies and speech language pathologist consultation (F = 7.67, p < 0.01). In Maringelli et al (2013), significant improvements in communication were noted in the following areas; expressing fundamental needs (z = −3.48), expressing needs and desires (z = −3.54), answering questions from hospital staff (z = −3.46) and communication/interaction with family (z = −3.5). In Rodriguez et al (2016), No statistical significance was discovered between control and intervention groups (−0.06, p = 0.14) despite the trend of improved ease of communication over repeated measures. | | VERY LOW |  |
|  |  | Ease or difficulties in communication  (Low-technology) | In Otuzoglu et al (2014), 42.2% were appropriate compared to 20.0% in the control which was a statistically significant increase p< 0.05. | | LOW |  |
|  |  | Communication success  (High-technology) | In Happ et al (2014), the intervention groups exhibited a significantly greater percentage of successful communication exchanges about pain than did the control group (OR 4.18); | | VERY LOW |  |
|  |  | Communication success  (Low-technology) | In Happ et al (2014), the intervention groups exhibited a significantly greater percentage of successful communication exchanges about pain than did the control group (OR 3.87); | | VERY LOW |  |
| Michalsen et al  2019 | To examine existing evidence regarding interprofessional shared decision-making, describe its principles and provide ICU clinicians with recommendations regarding its implementation. | Satisfaction with decision-making | The correlation between the total collaboration score and satisfaction scores for the decision-making process related to level of aggressiveness of care (for nurses, r = 0.70, p < 0.001; for residents, r = 0.50, p < 0.001). | | VERY LOW* | Clinicians should consider an interprofessional shared decision-making model that allows for the exchange of information, deliberation, and joint attainment of important treatment decisions. |
|  |  | Frequency of moral distress  (ICU professionals) | The correlation between nurse-physician collaboration and frequency of moral distress (r = -0.169, p < 0.0001) | | VERY LOW* |  |
|  |  | Severity of moral distress  (ICU professionals) | The correlation between nurse-physician collaboration and severity of moral distress (r = -0.215, p < 0.0001). | | VERY LOW* |  |
|  |  | Job satisfaction  (ICU professionals) | The correlation between nurse-physician collaboration and work satisfaction (r = 0.276, p < 0.001). | | VERY LOW* |  |
|  |  | Intention to resign  (ICU professionals) | The correlation between nurse-physician collaboration and intention to resign (r = -0.155, p < 0.001). | | VERY LOW* |  |
| Interventions related to reducing the duration of mechanical ventilation-Weaning modes or protocols | | | | | | |
| Ou-Yang et al  2020 | To compare proportional assist ventilation and pressure support ventilation as weaning methods to facilitate liberation from mechanical ventilation. | Weaning success | RR 1.16  (1.07, 1.26) | 634  (7 RCTs) | MODERATE* | Proportional assist ventilation is superior to pressure support ventilation in terms of weaning success, and the statistical power is confirmed using trial sequential analysis. |
|  |  | Reintubation | RR 0.49  (0.28, 0.87) | 484  (6 RCTs) | MODERATE* |  |
|  |  | Mortality | RR 0.66  (0.42, 1.06) | 470  (5 RCTs) | MODERATE* |  |
|  |  | ICU LOS | MD -1.58  (-2.68, -0.47) | 276  (5 RCTs) | MODERATE* |  |
|  |  | Duration of weaning | MD -0.01  (-1.3, 1.28) | 122  (3 RCTs) | LOW* |  |
|  |  | Duration of MV | MD -40.26  (-66.67, -13.48) | 133  (3 RCTs) | LOW* |  |
| Kataoka et al  2018 | To examine whether proportional modes improve patient-ventilator interaction and whether they have an impact on the weaning success and length of MV in mechanically ventilated patients, in comparison with pressure support ventilation. | Asynchrony index > 10% | RR 0.15  (0.04, 0.58) | 261  (7 RCTs) | VERY LOW | The use of proportional modes was associated with a reduction in the incidence with asynchrony index > 10%, weaning failure and duration of MV, compared with pressure support ventilation. However, reduced weaning failure and duration of MV were found with only proportional assist ventilation. |
|  |  | Asynchrony index > 10%  (proportional assist ventilation) | RR 0.06  (0.01, 0.46) | 110  (2 RCTs) | LOW |  |
|  |  | Asynchrony index > 10%  (neutrally adjusted ventilatory assist) | RR 0.20  (0.05, 0.92) | 151  (5 RCTs) | VERY LOW |  |
|  |  | Weaning failure  (proportional assist ventilation) | RR 0.44  (0.28, 0.75) | 317  (3 RCTs) | LOW |  |
|  |  | Duration of MV  (proportional assist ventilation) | MD -2.55  (-3.86, -0.65) | 318  (3 RCTs) | LOW |  |
|  |  | ICU LOS | MD -1.41  (-3.90, 1.09) | 528  (6 RCTs) | LOW |  |
|  |  | Hospital LOS | MD -0.26  (-3.90, 3.37) | 320  (5 RCTs) | LOW |  |
|  |  | Duration of weaning | MD-1.21  (-4.32, 1.91) | 220  (3 RCTs) | LOW |  |
|  |  | Reintubation | RR 0.39  (0.17, 0.90) | 158  (3 RCTs) | LOW |  |
|  |  | Use of NIV after extubation | RR 0.64  (0.47, 0.89) | 178  (2 RCTs) | LOW |  |
| Neuschwander et al  2021 | To compare the major automated modes for MV weaning in critically ill and post-operative adult patients. | Duration of weaning  (Adaptative support ventilation) | MD -0.36  (-0.64, -0.09) | 484  (5 RCTs) | MODERATE | Compared to standard weaning practice, all automated modes significantly reduced the duration of MV weaning in critically ill adult patients. |
|  |  | Duration of weaning  (Proportional Assist Ventilation) | MD -0.84  (-1.37, -0.31) | 50  (1 RCT) | MODERATE |  |
|  |  | Duration of weaning  (Smartcare) | MD -0.19  (-0.35, -0.03) | 800  (7 RCTs) | HIGH |  |
| Hirzallah et al  2019 | To synthesize the current best evidence for the effectiveness of weaning protocols led by nurses compared with usual physician-led care. | Duration of MV | MD -1.69  (-3.23, -0.16) | 532  (1RCT, 2 observational studies) | VERY LOW | The use of nurse-led weaning protocols for mechanically ventilated adult patients has a positive impact on weaning outcomes and patient safety. |
|  |  | ICU LOS | MD -2.04  (-2.57, -1.52) | 532  (1RCT, 2 observational studies) | LOW |  |
|  |  | Hospital LOS | MD -2.9  (-4.24, -1.56) | 532  (1RCT, 1 observational study) | VERY LOW |  |
| Interventions related to reducing the duration of mechanical ventilation-Ventilator bundle or cough augmentation techniques | | | | | | |
| Pileggi et al  2018 | To assess the effectiveness of the ventilator bundle in the reduction of mortality in ICU patients. | Mortality | OR 0.90  (0.84, 0.97) | 11816  (13 observational studies) | VERY-LOW | Simple interventions in common clinical practice applied in a coordinated way as a part of a bundle care are effective in reducing mortality in ventilated ICU patients. |
|  |  | ICU LOS | SMD -0.16  (-0.21, -0.12) | 6555  (7 observational studies) | VERY-LOW |  |
|  |  | Hospital LOS | SMD -0.12  (-0.18, -0.06) | 4272  (3 observational studies) | VERY-LOW |  |
|  |  | Duration of MV | SMD -0.18  (-0.23, -0.13) | 6956  (6 observational studies) | VERY-LOW |  |
| Rose et al  2017 | To determine extubation success using cough augmentation techniques compared to no cough augmentation for critically-ill adults | Extubation success | RR 1.58  (1.13, 2.20) | 75  (1 RCT) | VERY-LOW* | Very low-quality evidence from single trial findings suggests that cough-promoting techniques might increase successful removal of the breathing tube and decrease the time spent on mechanical ventilation, while not causing harm. The limited participant numbers made it difficult to determine the likelihood of harms. |
|  |  | Duration of MV | MD -6.1  (-8.4, -3.8) | 75  (1 RCT) | VERY-LOW* |  |
|  |  | Adverse events | RR 3.4  (0.1. 81.3) | 75  (1 RCT) | VERY-LOW* |  |
| Interventions related to reducing the duration of mechanical ventilation-Early tracheostomy | | | | | | |
| Wang et al  2019 | To assess tracheotomy timing for critically ill ventilated patients and determine the outcomes’ reliability | Duration of MV | SMD -0.91  (-1.45 to -0.38) | 2017  (12 RCTs) | LOW | Early tracheotomy seems to be associated with a lower incidence of ventilator-associated pneumonia, shorter duration of MV, shorter duration of sedation, and shorter ICU stay. However, the apparent benefits revealed in traditional meta-analysis contrast with the post- trial sequential analysis results. |
|  |  | Duration of sedation | SMD -1.41  (-2.09, -0.73) | 2116  (7 RCTs) | LOW |  |
|  |  | ICU LOS | SMD -1.08  (-1.61, -0.56) | 2720  (11 RCTs) | LOW |  |
|  |  | ICU mortality | OR 0.85  (0.68, 1.06) | 1606  (9 RCTs) | LOW |  |
|  |  | Hospital mortality | OR 0.85,  (0.69, 1.04) | 1588  (5 RCTs) | MODERATE |  |
| McCredie et al  2017 | To systematically review all RCTs and quasi-randomized controlled trials comparing early tracheostomy to late tracheostomy or prolonged intubation in acutely brain-injured patients to determine effects on long-term, all-cause mortality. | Duration of MV | MD -2.72  (-4.15, -1.29) | 412  (8 RCTs) | LOW | Early tracheostomy in acutely brain-injured patients may reduce long-term mortality, duration of mechanical ventilation, and ICU length of stay. However, waiting longer leads to fewer tracheostomy  procedures and similar short-term mortality. |
|  |  | ICU LOS | MD -2.55  (-4.15, -1.29) | 326  (6 RCTs) | LOW |  |
|  |  | Hospital mortality | RR 1.17  (0.46, 2.94) | 112  (4 RCTs) | VERY LOW |  |
|  |  | ICU mortality | RR 0.46  (0.24, 0.89) | 197  (3 RCTs) | LOW |  |
|  |  | Long-term mortality | RR 0.57  (0.36, 0.90) | 135  (3 RCTs) | LOW |  |
|  |  | Number of tracheostomy procedures performed | RR 1.58  (1.24, 2.02) | 377  (7 RCTs) | VERY LOW |  |
|  |  | Laryngotracheal complications | RR 2.54  (0.46, 13.88) | 222  (4 RCTs) | VERY LOW |  |
| de Franca et al  2020 | To elucidate the impact of early tracheostomy on hospitalization  outcomes in patients with traumatic brain injury. | Duration of MV | MD -4.15  (-6.30, -1.99) | 1202  (1 RCT, 3 observational studies) | VERY LOW | Early tracheostomy in severe traumatic brain injury patients contributes to a lower exposure to secondary insults and nosocomial adverse events, increasing the opportunity of patient’s early rehabilitation and discharge. |
|  |  | ICU LOS | MD -5.87  (-8.74, -3.00) | 1368  (5 observational studies) | VERY LOW |  |
|  |  | Hospital LOS | MD -6.68  (-8.03, -5.32) | 1312  (4 observational studies) | VERY LOW |  |
|  |  | Mortality | RD 0.03  (-0.02, 0.07) | 4606  (1 RCT, 6 observational studies) | VERY LOW |  |
| Interventions related to reducing the duration of mechanical ventilation-High-flow nasal cannula | | | | | | |
| Granton et al  2020 | To determine the safety and efficacy of HFNC after extubation in critically ill adults. | Reintubation - COT Comparator | RR 0.46  (0.30, 0.70) | 847  (4 RCTs) | MODERATE* | High-flow nasal cannula reduces reintubation compared with conventional oxygen therapy, but not compared with noninvasive ventilation after extubation. |
|  |  | Reintubation - NIV Comparator | RR 1.16  (0.86, 1.57) | 730  (3 RCTs) | LOW* |  |
|  |  | Mortality - COT Comparator | RR 0.93  (0.57, 1.52) | 787  (3 RCTs) | MODERATE* |  |
|  |  | Mortality - NIV Comparator | RR 1.12  (0.82, 1.53) | 642  (2 RCTs) | MODERATE* |  |
|  |  | Need for NIV - COT Comparator | RR 0.64  (0.34, 1.22) | 320  (3 RCTs) | MODERATE* |  |
|  |  | ICU LOS - COT Comparator | MD -0.05  (-0.83, 0.73) | 787  (3 RCTs) | HIGH* |  |
|  |  | ICU LOS - NIV Comparator | MD -0.99  (-1.68, -0.30) | 642  (2 RCTs) | MODERATE* |  |
|  |  | Hospital LOS - COT Comparator | MD -0.98  (-2.16, 0.21) | 682  (2 RCTs) | MODERATE* |  |
|  |  | Hospital LOS - NIV Comparator | MD -3.00  (-6.24, 0.24) | 600  (1 RCT) | MODERATE* |  |
|  |  | Comfort - COT Comparator | MD -2.25  (-5.68, 1.18) | 94  (2 RCTs) | LOW* |  |
|  |  | Comfort - NIV Comparator | MD -1.60  (-2.88, -0.32) | 42  (1 RCT) | MODERATE* |  |
|  |  | Post-Extubation Respiratory Failure - COT Comparator | RR 0.52  (0.30, 0.91) | 787  (3 RCTs) | VERY LOW* |  |
|  |  | Post-Extubation Respiratory Failure - NIV Comparator | RR 0.82  (0.48, 1.41) | 730  (3 RCTs) | VERY LOW* |  |
| Chaudhuri et al  2020 | To examine the benefits of high-flow nasal cannula in the peri-intubation period | Peri-intubation hypoxia (assessed with: Lowest SpO_2_ below 80%) | RR 0.98  (0.68, 1.42) | 884  (7 RCTs) | MODERATE* | Moderate-to-low certainty evidence proved that the use of high-flow nasal cannula likely has no effect on severe desaturation, serious complications, apneic time, oxygenation, ICU length of stay, or overall survival when used in the peri-intubation period when compared with conventional oxygen therapy. |
|  |  | 28 Day Mortality | RR 0.91  (0.72, 1.12) | 665  (4 RCTs) | MODERATE* |  |
|  |  | Peri-intubation complications (assessed with: severe immediate complications including severe hypoxia, significant hypotension and pressor use, and cardiac arrest) | RR 0.87  (0.71, 1.06) | 884  (7 RCTs) | LOW* |  |
|  |  | ICU LOS | MD -1.15  (-2.45, 0.16) | 665  (4 RCTs) | MODERATE* |  |
|  |  | Apneic time (assessed with: seconds) | MD 10.3  (-11, 31.7) | 610  (7 RCTs) | LOW* |  |
|  |  | PaO_2_ post intubation (assessed with: mm Hg) | MD 27  (-13.2, 67.2) | 329  (6 RCTs) | MODERATE* |  |
|  |  | PaO_2_ after pre-oxygenation (assessed with: mm Hg) | MD 3.6  (-3.5, 10.7) | 786  (7 RCTs) | MODERATE* |  |
|  |  | PaCO_2_ post intubation (assessed with: mm Hg) | MD -0.6  (-3.4, 2.2) | 208  (4 RCTs) | LOW* |  |
| Zhu et al  2019 | To robustly quantify the benefits of high-flow nasal cannula for patients after planned extubation by investigating postextubation respiratory failure and other outcomes. | Postextubation respiratory failure | RR 0.62  (0.42, 0.92) | 1067  (5 RCTs) | HIGH* | Compared with conventional oxygen therapy, high-flow nasal cannula may significantly reduce postextubation respiratory failure and respiratory rates, increase PaO_2_, and be safely administered in patients after  planned extubation. |
|  |  | PaO_2_ (mmHg) | MD 0.30  (0.04, 0.56) | 497  (5 RCTs) | HIGH* |  |
|  |  | Respiratory rates (breaths per minute) | MD -0.70  (-1.16, -0.25) | 311  (5 RCTs) | HIGH* |  |
|  |  | Reintubation | RR 0.58  (0.30, 1.11) | 1562  (7 RCTs) | HIGH* |  |
| Huang et al  2018 | To evaluate the effect of high-flow nasal cannula on reintubation in adult patients. | Reintubation - COT Comparator | RR 0.58  (0.21, 1.60) | 1347  (5 RCTs) | LOW* | High-flow nasal cannula may be a potential alternative respiratory support to conventional oxygen therapy and noninvasive ventilation, with the latter often associating with patient intolerance and requiring a monitored setting. |
|  |  | Reintubation - NIV Comparator | RR 1.11  (0.88, 1.40) | 1434  (2 RCTs) | HIGH* |  |
| Lewis et al  2021 | To assess the effectiveness of HFNC compared to standard oxygen therapy, or NIV or NIPPV, for respiratory support in adults in the ICU. | Treatment failure - COT Comparator | RR 0.62  (0.45, 0.86) | 3044  (15 RCTs) | LOW* | HFNC may lead to less treatment failure when compared to standard oxygen therapy, but probably makes little or no difference to treatment failure when compared to NIV or NIPPV. However, the evidence was often of low or very low certainty. |
|  |  | In-hospital mortality- COT Comparator | RR 0.96  (0.82, 1.11) | 2673  (11 RCTs) | MODERATE* |  |
|  |  | Respiratory infection (pneumonia) - COT Comparator | RR 0.72  (0.48, 1.09) | 1057  (4 RCTs) | MODERATE* |  |
|  |  | Nasal mucosa or skin trauma- COT Comparator | RR 3.66  (0.43, 31.48) | 617  (2 RCTs) | VERY LOW* |  |
|  |  | ICU LOS- COT Comparator | MD 0.12  (-0.03, 0.27) | 1014  (7 RCTs) | LOW* |  |
|  |  | Respiratory effects: PaO_2_/FiO_2_ ratio up to 24 hours after initiation of therapy - COT Comparator | MD 10.34  (-17.31, 38.00) | 600  (5 RCTs) | VERY LOW* |  |
|  |  | Comfort (short-term effect) - COT Comparator | MD 0.31  (-0.61, 1.22) | 662  (4 RCTs) | VERY LOW* |  |
|  |  | Comfort (long-term effect) - COT Comparator | MD 0.59  (-2.29, 3.47) | 445  (2 RCTs) | VERY LOW* |  |
|  |  | Treatment failure - NIV or NIPPV Comparator | RR 0.98  (0.78, 1.22) | 1758  (5 RCTs) | LOW* |  |
|  |  | In-hospital mortality - NIV or NIPPV Comparator | RR 0.92  (0.64, 1.31) | 1758  (5 RCTs) | LOW* |  |
|  |  | Respiratory infection (pneumonia) - NIV or NIPPV Comparator | RR 0.51  (0.17, 1.52) | 1750  (3 RCTs) | VERY LOW* |  |
|  |  | Barotrauma (pneumothorax) - NIV or NIPPV Comparator | RR 1.15  (0.42, 3.14) | 830  (1 RCTs) | LOW* |  |
|  |  | ICU LOS -NIV or NIPPV Comparator | MD -0.72  (-2.85, 1.42) | 246  (2 RCTs) | LOW* |  |
|  |  | Respiratory effects: PaO_2_/FiO_2_ ratio up to 24 hours after initiation of therapy -NIV or NIPPV Comparator | MD -58.1  (-71.68, 44.51) | 1086  (3 RCTs) | LOW* |  |
|  |  | Comfort (short-term effect) - NIV or NIPPV Comparator | MD 1.33  (0.74, 1.92) | 258  (2 RCTs) | VERY LOW* |  |
|  |  | Comfort (long-term effect) - NIV or NIPPV Comparator | 1 study (304 participants) reported little or no difference between types of respiratory support, with comfort rated as 'poor', 'acceptable' or 'good'. | | VERY LOW* |  |
| Zhao et al  2017 | To investigate whether HFNC was superior to either COT or NIV in adult acute respiratory failure patients. | Intubation rate - COT Comparator | OR 0.52  (0.34, 0.79) | 1854  (8 RCTs) | MODERATE* | Compared to COT, HFNC reduced the rate of intubation, mechanical ventilation and the escalation of respiratory support. When compared to NIV, HFNC showed no better outcomes. |
|  |  | MV rate - COT Comparator | OR 0.56  (0.33, 0.97) | 1914  (9 RCTs) | LOW* |  |
|  |  | Escalation rate - COT Comparator | OR 0.45  (0.31, 0.67) | 1914  (9 RCTs) | MODERATE* |  |
|  |  | Mortality - COT Comparator | OR 1.01  (0.67, 1.53) | 1497  (5 RCTs) | LOW* |  |
|  |  | Intubation rate - NIV Comparator | OR 0.96  (0.66, 1.39) | 1651  (3 RCTs) | VERY LOW* |  |
|  |  | Escalation rate - NIV Comparator | OR 1.00  (0.77, 1.28) | 1651  (3 RCTs) | LOW* |  |
|  |  | Mortality - NIV Comparator | OR 0.85  (0.43, 1.68) | 1651  (3 RCTs) | VERY LOW* |  |
| Rehabilitation and mobilization-Rehabilitation techniques | | | | | | |
| Waldauf et al  2020 | To assess the impact of rehabilitation in ICU on clinical outcomes. | End of study  mortality | RR 0.94  (0.79, 1.12) | 3199  (38 RCTs) | LOW* to MODERATE* | Rehabilitation interventions in critically ill patients do not influence mortality and are safe. Protocolized physical rehabilitation significantly shortens time spent on mechanical ventilation and in ICU, but this does not consistently translate into long-term functional benefit. Stable patients with lower Acute Physiology and Chronic Health Evaluation II at admission (<20) and prone to protracted ICU stay may benefit most from rehabilitation interventions. |
|  |  | ICU LOS  (supine cycling) | MD 1.2  (-1.7, 4.1) | 380  (8 RCTs) | VERY-LOW* |  |
|  |  | ICU LOS  (neuromuscular electrical stimulation) | MD -1.2  (-3.7, 1.3) | 358  (7 RCTs) | VERY-LOW* |  |
|  |  | LCU LOS  (Protocolized physical therapy) | MD -2.0  (-3.6, -0.3) | 1616  (16 RCTs) | LOW* |  |
|  |  | Long-term functional outcome | Conflicting results and various outcomes reported, low treatment effect even in positive studies. | | VERY-LOW* |  |
| Nydahl et al  2017 | To synthesize safety data regarding patient mobilization and rehabilitation in the ICU, including falls, removal of endotracheal tubes, removal or dysfunction of intravascular catheters, removal of other catheters/tubes, cardiac arrest, hemodynamic changes, and desaturation | Fall | In 27 included studies, the number of mobilization/rehabilitation sessions was 16342 and reported events was 11 (0.07%). | | LOW | Patient mobilization and physical rehabilitation in the ICU appears safe, with a low incidence of potential safety events, and only rare events having any consequences for patient management. |
|  |  | Endotracheal tube removal | In 28 included studies, the number of mobilization/rehabilitation sessions was 17148 and reported events was 2 (0.01%). | | LOW |  |
|  |  | Intravascular catheter event | In 31 included studies, the number of mobilization/rehabilitation sessions was 16397 and reported events was 35 (0.2%). | | LOW |  |
|  |  | Other catheter or tube removal | In 25 included studies, the number of mobilization/rehabilitation sessions was 15761 and reported events was 15 (0.09%). | | LOW |  |
|  |  | Cardiac arrest | In 26 included studies, the number of mobilization/rehabilitation sessions was 14438 and reported events was 4 (0.03%). | | LOW |  |
|  |  | Hemodynamic changes | In 33 included studies, the number of mobilization/rehabilitation sessions was 18083 and reported events was 126 (0.7%). | | LOW |  |
|  |  | Desaturation | In 33 included studies, the number of mobilization/rehabilitation sessions was 16487 and reported events was 78 (0.5%). | | LOW |  |
| Rehabilitation and mobilization-Early mobilization | | | | | | |
| Zang et al  2020 | To assess if early mobilization and rehabilitation in the ICU could reduce ICU-acquired weakness, improve functional recovery, improve muscle strength, shorten the length of ICU and hospital stays, and reduce the mortality rate | ICU-acquired weakness | RR 0.49  (0.26, 0.91) | 892  (7 RCTs) | LOW | Early mobilization was effective in preventing the occurrence of ICU-acquired weakness, shortening the length of ICU and hospital stay, and improving the functional mobility. However, it had no effect on the ICU mortality rate and ventilator-free days. |
|  |  | ICU mortality rate | RR 1.21  (0.97, 1.76) | 923  (7 RCTs) | MODERATE |  |
|  |  | ICU LOS | MD -1.82  (-2.88, -0.76) | 1345  (11 RCTs) | LOW |  |
| Zhang et al  2019 | To assess the evidence available regarding the effect of early mobilization on critically ill patients in the ICU. | Duration of MV | SMD -0.33  (-0.66, 0) | 1501  (17 RCTs) | LOW | Early mobilization appears to decrease the incidence of ICU-AW, improve the functional capacity, and increase the number of ventilator-free days and the discharged-to-home rate for patients with a critical illness in the ICU setting. |
|  |  | Ventilator-free days | SMD 0.17  (0.02, 0.31) | 745  (6 RCTs) | HIGH |  |
|  |  | 28-day mortality | RR 1.23  (0.81, 1.85) | 481  (3 RCTs) | MODERATE |  |
|  |  | ICU mortality | RR 1.12  (0.82, 1.52) | 940  (8 RCTs) | MODERATE |  |
|  |  | Hospital mortality | RR 1.10  (0.89, 1.37) | 711  (8 RCTs) | MODERATE |  |
|  |  | Adverse events | RR 1.35  (0.86, 2.12) | 1009  (8 RCTs) | HIGH |  |
| Doiron et al  2018 | To assess the effects of early intervention (mobilization or active exercise) on improving physical function or performance, muscle strength and health-related quality of life. | Adverse events | One study reported that in the intervention group 1/49 (2%) experienced oxygen desaturation < 80% and 1/49 (2%) had accidental dislodgement of the radial catheter. This study also found cessation of therapy due to patient instability occurred in 19/498 (4%) of the intervention sessions. In another study 5/101 (5%) of the intervention group and 5/109 (4.6%) of the control group had postoperative pulmonary complications. These were deemed to be unrelated to intervention. A third study found 1/150 in the intervention group had an episode of asymptomatic bradycardia, but completed the exercise session. | | LOW* | There is insufficient evidence on the effect of early mobilization of critically ill people in the ICU on physical function or performance, adverse events, muscle strength and health-related quality of life at this time. |
| Fuke et al  2018 | To examine the effectiveness of early rehabilitation for the prevention of post intensive care syndrome, characterised by an impaired physical, cognitive or mental health status, among survivors of critical illness. | Incidence of ICU-acquired weakness | OR 0.42  (0.22, 0.82) | 154  (2 RCTs) | LOW* | Early rehabilitation improved only short-term physical-related outcomes in patients with critical illness. |
|  |  | Delirium-free days | SMD -0.02  (-0.23, 0.2) | 196  (3 RCTs) | LOW* |  |
|  |  | Incidence of HADS/HAS | OR 0.79  (0.29, 2.12) | 92  (2 RCTs) | VERY-LOW* |  |
|  |  | Health related score | SMD 0.11  (-0.86, 1.09) | 63  (2 RCTs) | VERY-LOW* |  |
|  |  | SF-36 PF | SMD 2.41  (-0.75, 5.58) | 191  (2 RCTs) | LOW* |  |
| Worraphan et al  2020 | To compare the effectiveness and rank order of physical therapy interventions, including conventional physical therapy (CPT), inspiratory muscle training (IMT), and early mobilization (EM) on MV duration and weaning duration | Duration of MV  (EM vs CPT) | MD -2.00  (-3.57, -0.44) | 411  (7 RCTs) | LOW | IMT and CPT is the most appropriate therapeutic approach for the weaning duration, whereas EM is the most effective treatment to reduce the MV duration. IMT or EM was found to be superior to conventional physical therapy. |
|  |  | Duration of MV  (IMT+CPT vs CPT) | MD -2.12  (-6.96, 2.72) | 165  (3 RCTs) | MODERATE |  |
|  |  | Duration of MV  (IMT vs CPT) | MD -0.88  (-4.82, 3.06) | 38  (2 RCTs) | LOW |  |
|  |  | Duration of weaning  (EM+CPT vs CPT) | MD -0.75  (-2.39, 0.89) | 67  (1 RCT) | LOW |  |
|  |  | Duration of weaning  (EM vs CPT) | MD -0.78  (-1.70, 0.14) | 40  (1 RCT) | MODERATE |  |
|  |  | Duration of weaning  (IMT+CPT vs CPT) | MD -2.59  (-4.64, -0.53) | 333  (6 RCTs) | VERY-LOW |  |
|  |  | Duration of weaning  (IMT vs CPT) | MD -0.57  (-1.47, 0.33) | 38  (2 RCTs) | LOW |  |
| Rehabilitation and mobilization-Inspiratory Muscle Training | | | | | | |
| Wu et al  2019 | To evaluate the effect of threshold inspiratory muscle training (TIMT) on respiratory muscle strength and clinical outcomes for patients with MV. | Weaning failure | RR 0.66  (0.48, 0.92) | 435  (9 RCTs) | MODERATE* | TIMT could improve the massive inspiratory pressure and respiratory muscle strength of patients with MV, shorten the weaning time, the mechanical ventilation time and the ICU LOS, and then reduce the incidence of weaning failure. |
|  |  | Reintubation | RR 0.99  (0.56, 1.73) | 423  (7 RCTs) | VERY-LOW* |  |
|  |  | Mortality | RR 1.05  (0.53, 2.06) | 321  (6 RCTs) | MODERATE* |  |
|  |  | Duration of weaning | MD -1.01  (-1.65, -0.37) | 455  (9 RCTs) | LOW* |  |
|  |  | Duration of MV | MD -2.24  (-4.33, -0.15) | 412  (10 RCTs) | LOW* |  |
|  |  | ICU LOS | MD -3.41  (-6.06, -0.76) | 229  (4 RCTs) | MODERATE* |  |
| Vorona et al  2018 | To describe the range and tolerability of published methods for IMT and determine whether IMT improves respiratory muscle strength and clinical outcomes in critically ill patients. | Duration of MV | MD -4.07  (-7.35, -0.80) | 325  (9 RCTs) | VERY-LOW* | IMT is feasible and well tolerated in critically ill patients and improves both inspiratory and expiratory muscle strength. |
|  |  | Duration of weaning | MD -2.30  (-3.94, -0.67) | 257  (8 RCTs) | VERY-LOW* |  |
|  |  | ICU LOS | MD -3.05  (-7.07, 0.96) | 28  (2 RCTs) | VERY-LOW* |  |
|  |  | Mortality | RR 0.67  (0.20, 2.20) | 197  (3 RCTs) | LOW* |  |
| Management of specific symptoms-Delirium | | | | | | |
| Liang et al  2021 | To determine the effects of nonpharmacological interventions on preventing delirium and improving critically ill patients’ clinical, psychological, and family outcomes. | Delirium incidence | OR 0.43  (0.33, 0.55) | 6427  (6 RCTs, 22 observational studies) | LOW* | Healthcare professionals are recommended to apply early mobilisation, family participation, or multicomponent interventions in clinical practice to prevent delirium. |
|  |  | Delirium duration-days | MD -1.43  (-1.94, -0.92) | 2082  (3 RCTs, 8 observational studies) | LOW* |  |
|  |  | ICU LOS | MD -1.24  (-2.05, -0.43) | 4239  (3 RCTs, 15 observational studies) | LOW* |  |
|  |  | Mortality | OR 0.77  (0.55, 1.09) | 1283  (4 observational studies) | LOW* |  |
|  |  | Delirium incidence  (Multicomponent interventions) | OR 0.48  (0.34, 0.69) | 3172  (2 RCTs, 11 observational studies) | MODERATE* |  |
|  |  | Delirium duration-days  (Multicomponent interventions) | MD -1.47  (-2.20, -0.75) | 1666  (1 RCT, 6 observational studies) | LOW* |  |
|  |  | ICU LOS  (Multicomponent interventions) | MD -1.01  (-1.77, -0.25) | 2036  (1 RCT, 8 observational studies) | LOW* |  |
|  |  | Mortality  (Multicomponent interventions) | OR 0.51  (0.26, 0.97) | 419  (2 observational studies) | LOW* |  |
|  |  | Delirium incidence  (Early mobilisation) | OR 0.33  (0.24, 0.46) | 859  (2 RCTs, 3 observational studies) | MODERATE* |  |
|  |  | Delirium duration-days  (Early mobilisation) | MD -1.24  (-1.43, -1.04) | 416  (2 RCTs, 2 observational studies | MODERATE* |  |
|  |  | ICU LOS  (Early mobilisation) | MD -1.02  (-2.88, 0.84) | 282  (1 RCT, 1 observational study) | VERY-LOW* |  |
|  |  | Delirium incidence  (Family participation) | OR 0.25  (0.18, 0.34) | 997  (1 RCT, 3 observational studies) | MODERATE* |  |
|  |  | ICU LOS  (Family participation) | MD -2.31  (-4.14, -0.48) | 833  (3 observational studies) | VERY-LOW* |  |
|  |  | Delirium incidence  (Music) | OR 0.47  (0.28, 0.79) | 311  (1 RCT, 1 observational study) | LOW* |  |
|  |  | Delirium incidence  (Patient education) | OR 0.45  (0.24, 0.83) | 224  (2 observational studies) | LOW* |  |
|  |  | ICU LOS  (Patient education) | MD -5.30  (-13.11, 2.50) | 224  (2 observational studies) | VERY-LOW* |  |
|  |  | Delirium incidence  (Physical environment) | OR 1.16  (0.88, 1.53) | 864  (2 observational studies) | LOW* |  |
|  |  | Delirium duration-days  (Physical environment) | MD 0.15  (-0.49, 0.79) | 864  (2 observational studies | VERY-LOW* |  |
|  |  | Mortality  (Physical environment) | OR 0.92  (0.61, 1.39) | 864  (2 observational studies) | VERY-LOW* |  |
| Bannon et al  2019 | To evaluate the effect of non-pharmacological interventions versus standard care on incidence and duration of delirium in critically ill patients. | Incidence of delirium  (Bright light therapy) | RR 0.45  (0.10, 2.13) | 829  (4 RCTs) | VERY-LOW* | Current evidence does not support the use of non-pharmacological interventions in reducing incidence and duration of delirium in critically ill patients. |
|  |  | Incidence of delirium  (Multicomponent intensive occupational therapy) | RR 0.15  (0.02, 1.03) | 130  (1 RCT) | VERY-LOW* |  |
|  |  | Incidence of delirium  (Earplugs) | RR 1.05  (0.43, 2.54) | 136  (1 RCT) | VERY-LOW* |  |
|  |  | Incidence of delirium  (Multicomponent (orientation  and cognitive stimulation)) | RR 0.91  (0.18, 4.73) | 48  (1 RCT) | VERY-LOW* |  |
|  |  | Incidence of delirium  (Protocolised sedation) | RR 0.98  (0.77, 1.23) | 423  (1 RCT) | VERY-LOW* |  |
|  |  | Incidence of delirium  (Multicomponent (risk factor targeting)) | RR 0.60  (0.27, 1.35) | 123  (1 RCT) | VERY-LOW* |  |
|  |  | Incidence of delirium  (Structured mirrors) | RR 1.10  (0.51, 2.40) | 223  (1 RCT) | VERY-LOW* |  |
|  |  | Incidence of delirium  (Range of motion exercises) | RR 0.40  (0.10, 1.67) | 94  (1 RCT) | VERY-LOW* |  |
|  |  | Duration of delirium-days  (Multicomponent physical therapy) | MD -0.65  (-2.73, 1.44) | 404  (2 RCTs) | LOW* |  |
|  |  | Duration of delirium-days  (Multicomponent intensive occupational therapy) | MD -0.17  (-0.91 to 0.57) | 130  (1 RCT) | VERY-LOW* |  |
|  |  | Duration of delirium-days  (Awakening and breathing) | MD -0.34  (-1.43 to 0.75) | 335  (1 RCT) | VERY-LOW* |  |
|  |  | Duration of delirium-days  (Bright light therapy) | MD -0.34  (-0.84 to 0.16) | 734  (1 RCT) | VERY-LOW* |  |
|  |  | Duration of delirium-days  (Structured mirrors) | MD 0.00  (-1.21, 1.21) | 223  (1 RCT) | VERY-LOW* |  |
|  |  | Duration of delirium-days  (Family voice orientation) | MD -1.30  (-2.41, -0.19) | 20  (1 RCT) | VERY-LOW* |  |
|  |  | Duration of delirium-days  (Range of motion) | MD 7.00  (− 24.41, 38.41) | 94  (1 RCT) | VERY-LOW* |  |
|  |  | Hospital mortality  (Multicomponent risk factors) | RR 0.32  (0.08, 1.31) | 123  (1 RCT) | VERY-LOW* |  |
|  |  | Hospital mortality  (Protocolised sedation) | RR 0.98  (0.66, 1.43) | 423  (1 RCT) | VERY-LOW* |  |
|  |  | Hospital mortality  (Physical rehabilitation) | RR 0.72  (0.27, 1.92) | 104  (1 RCT) | VERY-LOW* |  |
|  |  | Hospital mortality  (Bright light therapy) | RR 0.96  (0.64, 1.44) | 734  (1 RCT) | VERY-LOW* |  |
|  |  | Quality of life | SF-36 physical functioning and mental health scale scores not significantly different for a rehabilitation intervention at discharge, 2, 4 and 6 months. The EQ-5D Visual Analogue Scale and Index at 12 weeks were not significantly different for a structured mirrors intervention. | | VERY-LOW* |  |
|  |  | Adverse events | Protocolised sedation intervention, increase in self extubation in intervention group (6% difference; 95% CI 0.6 to 11.8). Early physical and occupational therapy intervention, desaturation in 1/498 therapy sessions; one accidental radial arterial line removal; ventilator dysynchrony in 4% of intervention group. Standardised rehabilitation intervention reported a similar number of adverse events in both groups. | | VERY-LOW* |  |
| León-Salas et al  2020 | To comprehensively assess the effects of multicomponent interventions for prevention of delirium in hospitalized older people. | Incidence of delirium | RR 0.30  (0.10, 0.88) | 160  (1 RCT) | LOW | This meta-analysis suggests that multicomponent interventions to prevent delirium are effective in decreasing its incidence, duration, and severity; as well as the incidence of pressure ulcers in hospitalized older people. Therefore, multicomponent interventions hold great promise to impact in the most important and prevalent conditions affecting fragile older people during hospitalization. |
|  |  | Duration of delirium-days | MD -1.33  (-1.83, -0.83) | 17  (1 RCT) | LOW |  |
|  |  | Severity of delirium | SMD -1.39  (-2.20, -0.58) | 31  (1 RCT) | LOW |  |
| Herling et al  2018 | To assess existing evidence for the effect of preventive interventions on ICU delirium, in-hospital mortality, the number of delirium- and coma-free days, ventilator-free days, length of stay in the ICU and cognitive impairment. | Hospital mortality  (Physical and cognitive therapy) | RR 0.94  (0.40, 2.20) | 65  (1 RCT) | VERY-LOW* | There is insufficient evidence to determine the effects of physical and cognitive intervention on delirium. The effects of sedation, environmental, and preventive nursing interventions are unclear and warrant further investigation in large multicentre studies. |
|  |  | Delirium- and coma-  free days | M -2.8  (-10.1, 4,6) | 65  (1 RCT) | VERY-LOW* |  |
|  |  | Ventilator-free days | Mdn D -2.1  (0.0, 29.2) | 65  (1 RCT) | VERY-LOW* |  |
|  |  | ICU LOS | MD 1.23  (-0.68, 3.14) | 65  (1 RCT) | VERY-LOW* |  |
|  |  | Cognitive functioning-Global functioning | M 0.97  (-0.19, 2.13) | 30  (1 RCT) | VERY-LOW* |  |
|  |  | Cognitive functioning-executive functioning | M -8.76  (-19.06, 1.54) | 30  (1 RCT) | VERY-LOW* |  |
|  |  | Adverse events | One patient experienced acute back pain accompanied by hypotensive urgency during physical therapy. | | VERY-LOW* |  |
| Lu et al  2019 | To clarify the efficacy of sleep and circadian interventions on preventing postoperative delirium. | Incidence of postoperative delirium  (earplugs and eye masks) | RR 0.15  (0.01, 2.73) | 41  (1 RCT) | LOW | In summary, currently only limited evidence suggests strategies targeted at sleep and circadian health as a useful way to prevent postoperative delirium. |
|  |  | Incidence of postoperative delirium  (timed bright light) | RR 0.24  (0.09, 0.66) | 94  (3 RCTs) | LOW |  |
| Litton et al  2016 | To assess the efficacy of earplugs as an ICU strategy for reducing delirium. | Incidence of delirium  (RCTs) | RR 0.58  (0.40, 0.84) | 194  (3 RCTs) | LOW | Placement of earplugs in patients admitted to the ICU, either in isolation or as part of a bundle of sleep hygiene improvement, is associated with a significant reduction in risk of delirium. |
|  |  | Incidence of delirium  (observational studies) | RR 0.57  (0.33, 0.97) | 638  (2 observational studies) | VERY-LOW |  |
|  |  | Hospital  mortality  (RCTs) | RR 0.85  (0.33, 2.17) | 435  (3 RCTs) | MODERATE |  |
|  |  | Hospital  Mortality  (observational studies) | RR 0.77  (0.53, 1.11) | 300  (1 observational study) | VERY-LOW |  |
| Management of specific symptoms-Agitation | | | | | | |
| Shetty et al  2018 | To assess the effects of BIS monitoring compared with clinical sedation assessment for mechanically ventilated adult participants in the ICU | ICU LOS | Mdn D 4  [Range 4 to 18] | 50  (1 RCT) | LOW* | The effects of BIS monitoring compared with CA of sedation in critically ill adults who were on a ventilator were insufficient. |
|  |  | Duration of MV | MD -0.02  (-0.13, 0.09) | 155  (2 RCTs) | LOW* |  |
|  |  | AE-restlessness | RR 1.11  (0.90. 1.37) | 105  (1 RCT) | VERY LOW* |  |
|  |  | AE-endotracheal tube resistance | RR 0.96  (0.75, 1.22) |  |  |  |
|  |  | AE-pain | RR 0.99  (0.89, 1.10) |  |  |  |
|  |  | AE-delirium | RR 3  (0.28, 32.04) |  |  |  |
| Aitken et al  2018 | To assess the effects of protocol-directed sedation management compared to usual care on the duration of mechanical ventilation, ICU and hospital mortality and other patient outcomes in mechanically ventilated ICU adults and children. | Duration of  MV | The mean duration of MV across control groups ranged from 93 to 228 hours; The mean duration of MV in the intervention groups was 28.15 hours shorter (95%CI -69.15 to 12.84) | | LOW | All of these included studies compared the use of protocol-directed sedation delivered by nurses to usual care (that is, non-protocol-directed sedation). There was no difference in the length of time mechanical ventilation was needed or in ICU or hospital deaths between people who received protocol-directed sedation and those people managed with usual care. There was a significant reduction in the number of days people treated with protocol-directed sedation spent in hospital, when compared to those managed with usual care. There was no difference between the two groups in the number of people who accidentally removed their breathing tube or required their tube to be reinserted after accidentally removing it. |
|  |  | ICU mortality | RR 0.77  (0.39, 1.50) | 513  (2 RCTs) | LOW* |  |
|  |  | Hospital mortality | RR 0.90  (0.72, 1.13) | 3082  (3 RCTs) | LOW* |  |
|  |  | ICU LOS | The mean length of hospital stay across control groups ranged from 6.0 days to 14.0 days; The mean length of hospital stay in the intervention groups was 1.70 days shorter (95% CI -3.71 to 0.31). | | LOW* |  |
|  |  | Hospital LOS | The mean length of hospital stay across control groups ranged from 19.2 days to 23.4 days; The mean length of hospital stay in the intervention groups was 3.09 days shorter (95% CI -5.08 to -1.10) | | MODERATE* |  |
|  |  | Adverse event-self extubation | RR 0.88  (0.55, 1.42) | 2761  (2 RCTs) | HIGH* |  |
|  |  | Adverse event-reintubation | RR 0.65  (0.35, 1.24) | 321  (1 RCT) | LOW* |  |
|  |  | Incidence of tracheostomy | RR 0.67  (0.35, 1.30) | 3082  (3 RCTs) | LOW* |  |
| Nassar et al  2016 | To systematically review studies that compared a mild target sedation protocol with daily sedation interruption and to perform a meta-analysis with the data presented in these studies. | Mortality | OR 0.81  (0.60, 1.10) | 892  (7 RCTs) | HIGH | Sedation protocols and daily sedation interruption do not appear to differ in regard to the majority of analyzed outcomes. The only differences found were small and had a high degree of heterogeneity. |
|  |  | Duration of MV | MD -1.52  (-3.60, 0.56) | 769  (6 RCTs) | MODERATE |  |
| Stephens et al  2018 | To define and quantify the impact of deep sedation within 48 hours of initiation of MV on the mortality, hospital and ICU LOS, MV duration, delirium and tracheostomy frequency. | Mortality | RR 0.34  (0.21, 0.54) | 4521  (2 RCTs, 7 observational studies) | VERY-LOW | Deep sedation in mechanically ventilated patients, as evaluated in a small number of qualifying heterogeneous randomized controlled trials and observational studies, was associated with increased mortality and lengths of stay. |
|  |  | Delirium incidence | OR 0.50  (0.22, 1.16) | 3861  (2 RCTs, 4 observational studies) | VERY-LOW |  |
|  |  | Tracheostomy | OR 0.58  (0.29, 1.16) | 673  (2 RCTs, 2 observational studies) | LOW |  |
|  |  | Duration of MV | MD -2.07  (-3.60, -0.53) | 4521  (2 RCTs, 7 observational studies) | VERY-LOW |  |
|  |  | ICU LOS | MD -2.98  (-5.38, -0.58) | 4016  (2 RCTs, 5 observational studies) | VERY-LOW |  |
|  |  | Hospital LOS | MD -5.92  (-13.84, 2.00) | 4016  (2 RCTs, 5 observational studies) | VERY-LOW |  |
| Long et al  2020 | To determine the association between different depths of sedation and the risk of delirium in adult mechanically ventilated patients. | Incidence of delirium | OR 1.00  (0.64, 1.58) | 8001  (10 RCTs, 8 observational studies) | MODERATE* | It is inconclusive whether significantly different sedation depths would change the risk of delirium in adult mechanically ventilated patients. |
|  |  | Incidence of agitation-related adverse events | OR 0.61  (0.45, 0.84) | 2282  (7 RCTs, 2 observational studies) | HIGH* |  |
|  |  | Mortality | OR 1.82  (1.23, 2,69) | 7804  (9 RCTs, 7 observational studies) | MODERATE* |  |
| Management of specific symptoms-Pain and sleep disturbances | | | | | | |
| Hadjibalassi et al  2018 | To review evidence on the effects of Guided imagery on physiological and psychological outcomes of adult critically ill patients. | Pain | Pain levels were quantified by either visual analogue or numeric rating scales. 6 studies that addressed pain levels, 3 reported statistically significantly lower pain intensity and 3 non-statistically significant trends for lower pain. | | VERY-LOW | On the basis of these results, and of the absence of reported side-effects, we conclude that guided imagery is a promising patient-centered approach for the improvement of a number of patients’ outcomes that merits further investigation in critical care. |
|  |  | Anxiety | A total of seven studies addressed anxiety/tension levels, either through NRS/VAS ratings or through POMS. All seven studies reported decreases in anxiety compared to the control group, but in only 3 studies the decrease in anxiety was statistically significant. | | VERY-LOW |  |
|  |  | Mortality | No difference between the intervention and control group was noted. | | LOW |  |
|  |  | ICU LOS | Overall, ICU LOS was explored in three studies addressed, with two reporting statistically significant decreases. | | VERY-LOW |  |
|  |  | Sleep quality | The effects of GI on sleep were explored in three studies with no statistically significant results | | VERY-LOW |  |
|  |  | Patient satisfaction | A non-statistically significant increase in patients’ satisfaction with care in the guided imagery compared to the standard care group. | | VERY-LOW |  |
| Umbrello et al  2019 | To assess the current evidence on the effectiveness of music therapy in reducing stress and anxiety in critically ill, adult patients | Anxiety | In Chlan et al (1998), Statistically significant difference in post-test state anxiety between groups: mean value 10.16 (M) vs. 16.15 (C), P<0.001; In Wong et al (2001), music  therapy was more effective than a rest period in reducing state anxiety (C 49.67±4.82 vs. M 38.67±5.23, P<0.01); In Lee et al (2005), Subjects in the music group had reduced state anxiety scores over time: 15.4±4.6 vs. 13.8±2.8, P=0.048; state anxiety in the control group was similar to baseline value; In Cooke et al (2010), No significant reduction of anxiety (M: 1.9 (1.4; 2.4) vs. 1.9 (1.2; 2.5) and C: 2.4 (1.8; 2.9) vs. 2.2 (1.5; 2.8); both P>0.05) in both groups; In Han et al (2010), Significant differences in C-STAI; In Korhan et al (2011), M: significantly lower respiratory rates, and systolic and diastolic blood pressure, than C, no differences in heart rate and oxygen saturation; In Chlan et al (2013), The intervention decreased anxiety exposure over time more effectively than usual care or placebo (noise-cancelling headphones); In Lee et al (2017), Significant better values for all post-test measures and for pre–post differences for music as compared to control (C-STAI M 57.2±7.64 vs. 51.5±5.1, C 57.2±5.8 vs. 56.2±5.6, p<0.001; VAS -A STAI M 57.2±9.2 vs. 49.6±8.1, C 58.0±8.2 vs. 56.1±9.2, P<0.001) | | MODERATE | Despite significant heterogeneity in trial designs, timing and features of the intervention, music therapy is consistently associated with a reduction in anxiety and stress of critically ill patients. |
|  |  | Stress and agitation | In Chlan et al (1998), reduction of heart and respiratory rate over time in both groups, with greater reduction in Music group; In Wong et al (2001), reduction of blood pressure and respiratory rate over time in both groups, with greater reduction in Music group; In Lee et al (2005), respiratory rate, heart rate, systolic and diastolic blood pressure were all significantly lower than baseline in the music group, while only diastolic blood pressure was reduced after the intervention in the control group; In Dijkstra et al (2010), no differences in blood pressure, heart or respiratory rate between groups; In Han et al (2010), significant reduction in stress response (heart respiratory rate) over time in music group while a significant increase in heart rate and respiratory rate over time in control, no significant change over time in headphone; In Su et al (2012), music group had significantly lower heart rates than control; In Lee et al (2017), significant better values for all post-test measures and for pre-post differences for music as compared to control except for diastolic blood pressure. | | MODERATE |  |
|  |  | Sedation | In Dijkstra et al (2010), Significantly higher level of sedation in music group (Ramsay M 3.8±0.8 vs. 4.3±0.7; C 4.6±0.9 vs. 4.0±1.4; P=0.015); In Chlan et al (2013), patients in M group had reduced sedation intensity by 0.18 points/day (P=0.05) and reduced frequency (0.21 points/day) vs. C and reduced sedation frequency (0.18 points/day) vs. P (P=0.04). | | LOW |  |
|  |  | Sleep quality | In Su et al (2012), improved polysomnography quality of sleep in music group (shorter N2 and longer N3 sleep), and improved self-reported sleep quality. | | LOW |  |

*Results from authors of systematic reviews

AAC: Augmentative and Alternative Communication

AE: Adverse events

BIS: Bispectral index

C: Control group

CA: Clinical assessment

CCFNI: Critical care family needs inventory

CI: Confidence interval

C-STAI: The Chinese version of the Spielberger State-Trait Anxiety Inventory Scale

COT: Conventional oxygen therapy

CPT: Conventional physical therapy

EGDM: early goal directed mobilization

EM: Early mobilization

FIL: family information leaflets

FSC: Family Support Co-Ordinator

HFNC: High-flow nasal cannula

ICU: Intensive care unit

ICU LOS: Intensive care unit length of stay

ICU-AW: Intensive care unit acquired weakness

IMT: Inspiratory muscle training

IES-R: Impacts of Events Scale-revised

M: Music intervention group

Mdn D: Median difference

MV: Mechanical ventilation

N: Number of participants

n: number of studies

NIPPV: non-invasive positive pressure ventilation

NIV: noninvasive ventilation

NRS: non-randomised studies

OR: Odds ratio

PA: physical activity

PaCO_2_: partial pressure of carbon dioxide

PMV: prolonged mechanical ventilation

PTSD: post-traumatic stress disorder

PTSS: Post-traumatic Stress Syndrome-14

PR: Physical restraint

RoM: Ratio of means

RR: Risk ratio

SBT: Spontaneous breathing trial

SF-36 PF: 36-Item Short Form Health Survey Physical Function scale
